# A Meta-Analysis of the Effects of High-LET Ionizing Radiations in Human Gene Expression

**DOI:** 10.3390/life11020115

**Published:** 2021-02-03

**Authors:** Theodora-Dafni Michalettou, Ioannis Michalopoulos, Sylvain V. Costes, Christine E. Hellweg, Megumi Hada, Alexandros G. Georgakilas

**Affiliations:** 1 DNA Damage Laboratory, Physics Department, School of Applied Mathematical and Physical Sciences, National Technical University of Athens, 157 80 Athens, Greece; daphnelettos@biol.uoa.gr; 2 Centre of Systems Biology, Biomedical Research Foundation, Academy of Athens, 115 27 Athens, Greece; imichalop@bioacademy.gr; 3 NASA Ames Research Center, Space Biosciences, Moffett Field, CA 94035, USA; sylvain.v.costes@nasa.gov; 4 Radiation Biology Department, Institute of Aerospace Medicine, German Aerospace Center (DLR), Linder Hoehe, 51147 Cologne, Germany; christine.hellweg@dlr.de; 5 Radiation Institute for Science & Engineering, Prairie View A&M University, Prairie View, TX 77446, USA

**Keywords:** microarrays, high-LET, space radiation, differential gene expression, meta-analysis, computational radiobiology, DNA damage response, functional enrichment analysis

## Abstract

The use of high linear energy transfer (LET) ionizing radiation (IR) is progressively being incorporated in radiation therapy due to its precise dose localization and high relative biological effectiveness. At the same time, these benefits of particle radiation become a high risk for astronauts in the case of inevitable cosmic radiation exposure. Nonetheless, DNA Damage Response (DDR) activated via complex DNA damage in healthy tissue, occurring from such types of radiation, may be instrumental in the induction of various chronic and late effects. An approach to elucidating the possible underlying mechanisms is studying alterations in gene expression. To this end, we identified differentially expressed genes (DEGs) in high Z and high energy (HZE) particle-, γ-ray- and X-ray-exposed healthy human tissues, utilizing microarray data available in public repositories. Differential gene expression analysis (DGEA) was conducted using the R programming language. Consequently, four separate meta-analyses were conducted, after DEG lists were grouped depending on radiation type, radiation dose and time of collection post-irradiation. To highlight the biological background of each meta-analysis group, functional enrichment analysis and biological network construction were conducted. For HZE particle exposure at 8–24 h post-irradiation, the most interesting finding is the variety of DNA repair mechanisms that were downregulated, a fact that is probably correlated with complex DNA damage formation. Simultaneously, after X-ray exposure during the same hours after irradiation, DNA repair mechanisms continue to take place. Finally, in a further comparison of low- and high-LET radiation effects, the most prominent result is that autophagy mechanisms seem to persist and that adaptive immune induction seems to be present. Such bioinformatics approaches may aid in obtaining an overview of the cellular response to high-LET particles. Understanding these response mechanisms can consequently aid in the development of countermeasures for future space missions and ameliorate heavy ion treatments.

## 1. Introduction

Human exposure to ionizing radiation can occur via interaction with various sources, such as the radioactivity emitted from naturally unstable atoms, cosmic radiation and other artificial sources. Regarding high linear energy transfer (LET) radiation exposure in particular, radiotherapy using particle radiation is starting to be incorporated in the treatment of specific tumors due to its improved physical properties and high relative biological effectiveness (RBE). In addition, in contrast to this clinical, local application of such types of radiation (mostly energetic carbon ions), astronauts experience chronic whole-body exposure to cosmic radiation during space flights. Such exposure to galactic cosmic radiation (GCR), which is made up of high-energy protons, relativistic helium ions and high-Z (charges greater than 2) and high energy particles (HZE particles) [1], is a major risk factor during long-term space missions. For example, during a mission to Mars, an astronaut could accumulate significant doses of radiation, at approximately 1 Sv [2]. Radiation carcinogenesis and non-cancer late effects such as cataract formation and degenerative alterations of the central nervous and cardiovascular systems are in the focus of space radiation risk assessments [3]. Space radiation exposure for long periods might also affect the immune system, which is already weakened by microgravity [4] and this could endanger the health of astronauts and, consequently, the success of the mission. Finally, there are still large uncertainties regarding effects related to the reproductive system and embryonic/fetal development concerning the safety of being pregnant after or during a long-term interplanetary mission [5].

In general IR-induced biological effects can be described by a variety of mechanisms and the local or generalized oxidative stress generated by the production of free radicals in the irradiated area or throughout the entirety of the body through systemic effects. This ongoing cell challenge can lead to genomic instability and cancer formation [6,7], while, at the same time, triggering various DNA damage response (DDR) mechanisms, considered as the major component of radiation response at the cellular level [8]. This group of mechanisms can be defined as the sum of functions that orchestrate DNA damage detection and the transduction of the appropriate signals. More specifically, the tumor suppressor protein p53 is a key factor in DDR mechanisms through signaling responses, which include cell cycle checkpoints, DNA repair and apoptosis activation [9]. The cell cycle transition from the G1 to S phase is vital for controlling cell proliferation, while its dysregulation promotes oncogenesis [10]. A G1 phase arrest provides the required time for DNA repair mechanisms to take action. If the repair fails, p53 levels decrease and cyclic-dependent kinase (CDK) activity restarts, leading to entry into the S phase while apoptotic mechanism activation is possible [11]. Erroneously repaired DNA damage can lead to mutations, while unrepaired damage can result in apoptosis or cell aging [12]. Moreover, p21 protein encoded by the *CDKN1A* gene, as the key gene activated by p53, is responsible for inhibiting cell proliferation in response to DNA damage [13]. This protein is also closely associated with DNA repair by contributing to the execution of apoptosis. Although considered to play a key role as a “guardian of the genome”, it may alternatively act as a mediator of genomic instability, cellular aging and carcinogenesis [14]. The IR response is also known to include systemic effects regarding inflammatory “danger” signal stimulation and innate immune response induction [15]. The chronic inflammatory response that may occur promotes cancer formation through increased DNA damage and inhibition of its repair pathways [16]. Finally, incorrect activation of the transcription factor nuclear factor kappa B (NF-κB) has been associated with a number of inflammatory diseases while persistent inhibition of NF-κB leads to improper growth of immune cells or delayed cell growth [17]. Overall deregulation of DDR mechanisms can cause several human diseases and conditions associated with cancer predisposition, accelerated aging and developmental abnormalities [18].

In the case of high-LET radiation exposure, persistent stress develops mainly due to the induction of complex DNA damage where oxidative and thermal stress are not limited to the target cell but spread to neighboring cells. DNA damage clusters are considered of great biological significance, as they are extremely repair-resistant and, in many cases, cannot be repaired, causing an overwhelming outcome on the cell or organism [19]. The two main categories of DNA damage are double-strand breaks (DSBs) and non-double-stranded oxidative lesions that occur in close proximity (10–20 bp) [20]. Moreover, bi-stranded clustered DNA lesions (in opposite strands) present remarkable complexity, as they become difficult to repair, thus increasing the probability of mutagenic or even fatal consequences to the cell [21]. In addition, DSBs have been shown to move into euchromatic regions of the nucleus in *Drosophila* [22] and yeast [23]. In mammalian cells, there is a growing body of work suggesting that DSBs also move into nuclear repair domains, leading to DSB clusters along HZE tracks or after exposure to high doses of low-LET radiations [24,25,26,27]. Finally, new sequences may appear in the irradiated areas, participating in complex rearrangements and resulting in visible chromosomal alterations, which usually result in toxicity [24,28] or carcinogenesis if cell death does not occur [29,30]. Therefore, complex DNA damage induction and DSB clustering into repair domains increase the frequency of inadequately repaired damage, which increases cytotoxicity and mutagenicity. Although multiple pathways are possible for the induction and persistence of genomic instability, DSBs and other forms of complex lesions are thought to be mainly involved in the development of chromosomal abnormalities [31].

High-LET particle exposure certainly has a negative effect on the immune system, as some of its components are considered the body’s most radiosensitive elements [32]. More specifically, it has been shown that lymphoid cells and tissues are markedly affected by high-LET radiation at relatively low doses and that some aberrations persist long after exposure, such as thymus and spleen atrophy and leucocyte population depletion [33]. In addition, immune balance alteration in the form of diminished natural killer and T cell functions and increased inflammatory plasma cytokine levels have been recorded [34]. However, the appropriate combination of radiotherapy and immunotherapy has delivered new perspectives regarding the treatment of metastatic and advanced cancers [35]. Furthermore, cranial radiotherapy used to prevent the progression of malignancy in the brain can cause progressive and possibly irreversible effects on cognitive function, including learning, memory, processing speed, attention and executive function [36]. Such treatments also result in other behavioral disorders which negatively affect anxiety, mood and depression [37,38]. For the above reasons, the mechanisms by which exposure to cosmic rays can disrupt the central nervous system (CNS) are troubling space agencies (e.g., the National Aeronautics and Space Administration (NASA)), as the neurocognitive complications that may occur jeopardize the success of the mission, the safety of astronauts and their quality of life after completion of the mission [39].

Epidemiological data from treated breast cancer patients suggest that radiation-related cardiovascular disease may occur even at doses <2 Gy targeted to the heart region [40]. However, data from cancer patients need to be carefully interpreted before being extrapolated to astronauts, who are a healthy group of people, and the exposure situation (tumor-targeted/partial vs. whole-body, fractionated vs. chronic, high vs. low dose) and types of radiation they receive may differ. Cosmic radiation, especially heavy ions (HZE particles), can significantly increase the risk of cardiovascular disease. HZE particles alter DNA methylation and the expression of genes associated with cardiovascular function and pathology, resulting in degenerative tissue changes and accelerated onset of atherosclerosis. For example, we recently showed, by looking at various gene expression datasets in NASA’s GeneLab Space Omics database [41], that HZE affects the cardiovascular system by the activation of FYN Proto-Oncogene, Src Family Tyrosine Kinase kinase through reactive oxygen species (ROS) [42]. At the same time, HZE exposure can increase the pro-inflammatory response and inhibit angiogenesis [43].

The association between exposure to high-LET particles and the development of metabolic disorders should also be investigated. In mice flown on the International Space Station, it was shown that lipotoxic pathways were activated during spaceflight lasting weeks to months, with increased lipid metabolic pathways detected by RNA sequencing analysis and Oil Red O (ORO) staining in the liver, for two different strains of mice flown in different missions. More recently, a multi-omics meta-analysis using a large array of GeneLab datasets and astronaut data has identified mitochondria as the main organelle being disrupted by space flight, both in mice and humans, with important consequences to metabolism [44]. Radiation exposure in general has been associated with an increased risk of developing chronic metabolic disorders such as insulin resistance and Type 2 diabetes [45]. However, disturbances in oxidative metabolism following exposure to particle radiation have been associated with reduced mitochondrial protein transport that persists for a long time after the decomposition of ROS. This condition may impair the stability and activity of DNA repair proteins [46]. In addition, while the oxidative mechanisms are negatively regulated, activation of the immune response is observed [47]. Consistent with radiation data, the large GeneLab study [44] suggested DNA damage from the urine and blood metabolic data compiled from the astronaut cohort and NASA Twin Study data, indicating mitochondrial stress as a consistent phenotype of spaceflight.

The objective of this work focused on studying the overall response to high-LET particles at a cellular level, in order to further elucidate how space radiation alone disrupts cells. This can aid in the advancement of prevention methods regarding risk factors in relation to cancer and other diseases or conditions [48], either in the case of therapeutic procedures or space missions, given that complete shielding in spacecrafts is impossible. From a systems biology perspective, the aforementioned cellular mechanisms can be examined through altered gene expression. Thus, we decided to approach the matter through a differential gene expression analysis (DGEA) of human tissues exposed to high-LET radiation, taking advantage of publicly available microarray data. To this end, we carefully selected three datasets of healthy human cell samples; in each of them, we identified differentially expressed genes (DEGs) between irradiated and non-irradiated cells. Subsequently, we performed three separate meta-analyses, followed by a functional enrichment analysis, aiming to highlight the differences in cellular response between low and high-LET exposure effects at a systems biology level.

## 2. Materials and Methods

**Figure 1:**
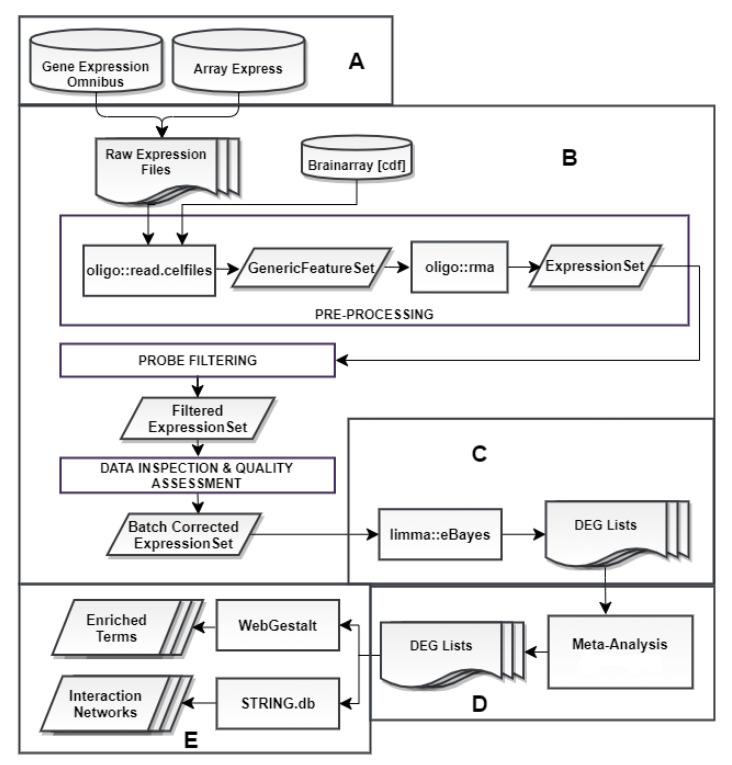
Our analysis workflow comprised of five steps: (**A**) Data collection from the online repositories Gene Expression Omnibus (GEO) and ArrayExpress. (**B**) Pre-processing that includes: (i) introduction of the corresponding CDF files from Brainarray (v.24) for probe summarization, (ii) execution of the Robust Multi-array Average (RMA) algorithm [49], (iii) probe filtering based on mean expressions [50], (iv) data inspection and quality assessment via principal component analysis (PCA) [51] and multi-dimensional scaling (MDS) [52] plots, followed by batch effect correction using the ComBat function of the sva package, if necessary [53,54]. (**C**) Differential gene expression analysis (DGEA) using the eBayes function of the limma [55] package. (**D**) Differentially expressed gene (DEG) list categorization into three groups, according to experimental condition and meta-analysis implementation. (**E**) Investigation of biological background of final DEG lists through WebGestalt [56] and STRING.db [57], in order to acquire enriched terms and protein–protein interaction (PPI) networks.

Our analysis can be summarized in a workflow (Figure 1). It commenced with a microarray data search conducted in public repositories and concluded with a functional enrichment analysis performed on statistically significant DEGs derived from 3 separate meta-analyses.

### 2.1. Primary Data Search

The search for our microarray data was conducted in Gene Expression Omnibus (GEO) [58] of the National Center for Biotechnology Information (NCBI), which serves as a public repository for gene expression data, in compliance with the requirements of Minimal Information About Microarray Experiments (MIAME) [59] and ArrayExpress [60] as a basic functional genomics data archive of the European Molecular Biology Laboratory—European Bioinformatics Institute (EMBL-EBI). Our selection criteria involved single-channel cDNA microarray experiments using human tissue samples, irradiated with high-LET HZE radiation. To this end, an advanced search of GEO was formulated as: (([(“expression profiling by array” [DataSet Type]) AND radiation, ionizing [MeSH Terms]] AND high LET[Description]) OR HZE[Description]) AND “*Homo sapiens*” [porgn], which yielded 8 data series. Subsequently, the two queries performed in ArrayExpress that yielded 2 additional datasets were: “ionizing radiation” AND “high-LET” and “ionizing radiation” AND “high LET”. Finally, datasets were manually curated in order to exclude non-irradiated, UV-irradiated and tumor samples, thus concluding in 3 data series.

### 2.2. Microarray Data Preprocessing and Differential Gene Expression Analysis

Pre-processing and DGEA were conducted in the R programming language [61], predominantly using Bioconductor (v.3.11) packages: oligo [62] and limma [55]. Provided that a data series included X-ray irradiated samples, data were also analyzed in terms of comparison with low-LET effects. Thus, data series with more than two distinct conditions were split in order to analyze genes that were differentially expressed between cells exposed to a specific type and dose of IR, for a specific time point of collection post-irradiation, and their corresponding control samples. Exported lists containing statistically significant DEGs, included metrics such as Log_2_ fold change (Log_2_FC), *p*-values and false discovery rate (FDR) [63] adjusted *p*-values for each gene. The lists were further annotated using org.Hs.eg.db (*v.3.8.2*) [64] to include HUGO Gene Nomenclature Committee (HGNC) [65] gene symbols and gene names.

### 2.3. Implementation of Meta-Analyses

DEG lists from the three (3) data series were further combined in three (3) separate meta-analyses to identify genes of differential expression within and across studies. In order to achieve optimal results, DEG lists were sorted into 3 groups (Table 1) depending on radiation type, radiation dose and time of collection post-irradiation before being subjected to a meta-analysis.

Our meta-analysis method combined unadjusted *p*-values of each study for every gene, using a weighted version of Stouffer’s meta-analysis [66], proposed by Mosteller and Bush [67], as previously described [68]. For each gene and study, its two-tailed unadjusted *p*-value was converted into a one-tailed *p*-value, based on the sign of the corresponding Log_2_FC. For each one-tailed *p*-value, the corresponding *z*-score was calculated using the inverse normal distribution function (*Φ*^−1^). The meta-analysis *p*-value for each gene was calculated, as shown in Equation (1) from the weighted *z*-score sum, using the normal distribution function (*Φ*) [67]:

(1) $$p=\Phi \left(\frac{\sum_{i\;=\;1}^{k}n_{i}\Phi^{-1}\left(p_{i}\right)}{\sqrt{\sum_{i\;=\;1}^{k}n_{i}{}^{2}}}\right)$$

where *p*_i_ is the eBayes-derived *p*-value, *n*_i_ is the number of samples of study *i* and *k* is the number of studies. Finally, *p*-values underwent FDR adjustment and 0.01 was selected as the threshold for statistical significance.

### 2.4. Functional Enrichment Analysis and Gene Network Construction

Our meta-analyses resulted in 3 final DEG lists, with the statistical significance cutoff for differential expression set to an adjusted *p*-value of <0.01. Consequently, each list was divided into up- and downregulated genes that were subjected to functional enrichment analysis in WebGestalt [56] using the Over-Representation Analysis (ORA) method [69]. As reference gene sets for biological processes and pathways, we selected Gene Ontology (GO) terms [70] and the KEGG, Reactome, Panther and WikiPathways databases [71,72,73,74], respectively. The statistical significance of each over-representation of biological term or pathway was estimated using hyper-geometric distribution and finally *p*-values were FDR-adjusted in order to distinguish terms with adjusted *p*-values of <0.01 as statistically significant.

Towards investigating the interactome of our three DEG lists and identifying possible underlying cell mechanisms, we constructed their protein–protein interaction (PPI) network, using STRING.db (*v.11.0*) [57]. The edges of the network, corresponding to protein interactions, were determined based on text mining and experimental sources with the highest confidence (Table A1).

## 3. Results

Queries performed in GEO and ArrayExpress identified ten (10) data series that fulfilled our search criteria. After manual curation, the repository accession numbers of the selected datasets were GSE63952 [75], E-MTAB-5754 [76], E-MTAB-3463 and E-MTAB-5761. Subsequently, we downloaded the corresponding CEL files containing raw expressions and CDF files from Brainarray (v.24). Finally, we selected samples that could be further combined in a meta-analysis (Table 2).

### 3.1. Differential Gene Expression

Our data series contained combinations of experimental conditions; therefore, were split into distinct studies (Table 3) before performing DGEA. The cutoff for statistical significance in differential expression was set as an FDR adjusted *p*-value of <0.05. Additionally, samples collected 7 days post-irradiation and samples exposed to 0.1 Gy of X-rays from data series E-MTAB-5754 and E-MTAB-3463, respectively, were excluded from the analysis, as they could not be sorted into a meta-analysis group. Finally, samples exposed to low doses of γ-rays from data series GSE63952 were also omitted.

**Figure 2:**
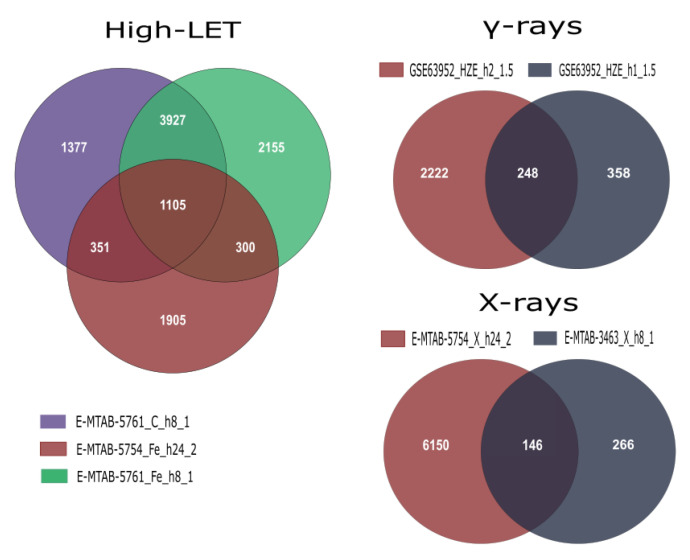
Venn diagrams illustrating overlapping DEGs within and across studies that were further combined in the three meta-analysis groups. Labels contain information on data series accession, radiation type, time of collection and radiation dose in Gy.

**Figure 3:**
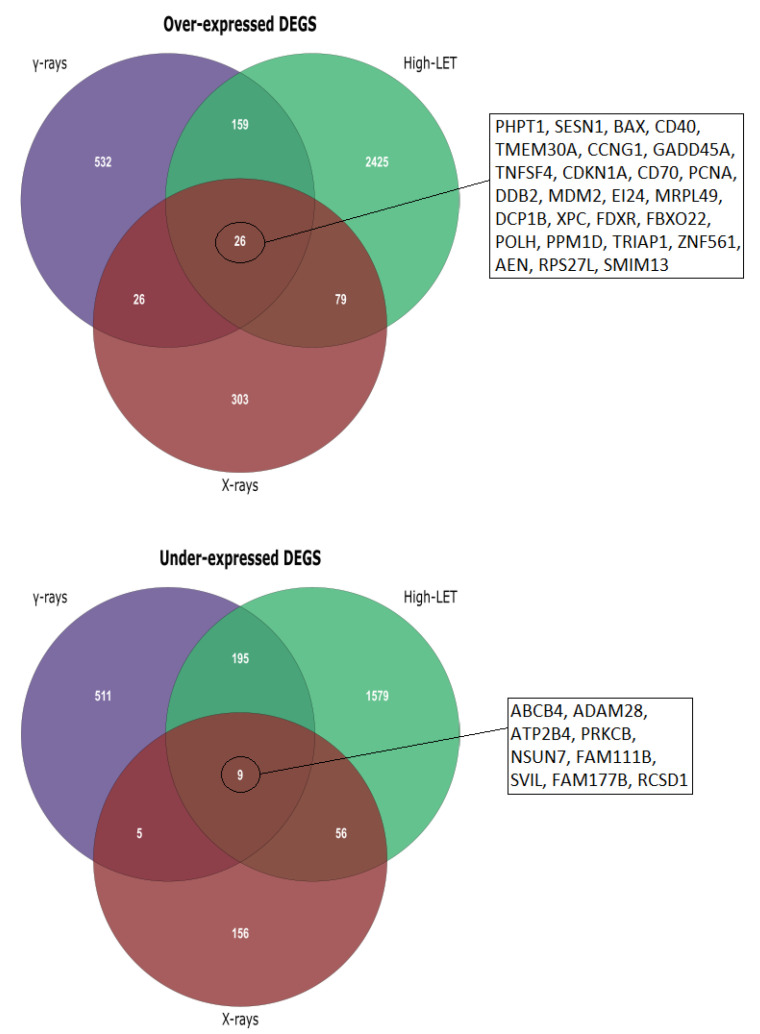
Venn diagrams for under- and overexpressed genes obtained after meta-analysis. Common DEGs in the three groups are depicted, while unique DEGs after exposure to high-LET radiation are included in the Supplementary Materials, Table S1.

After conducting the meta-analyses using the corresponding unfiltered gene lists derived from DGEA in R for each group (Figure 2), we acquired the final DEG lists by selecting the cutoff of an adjusted *p*-value of <0.01 (Table 4). In addition, statistically significant differentially expressed genes were divided into up- and downregulated, in order to perform functional enrichment analysis. Finally, an initial investigation for possible differentiation in expression patterns after exposure to low- and high-LET radiation was performed through Venn diagram creation (Figure 3).

### 3.2. Functional Enrichment Results

**Figure 4:**
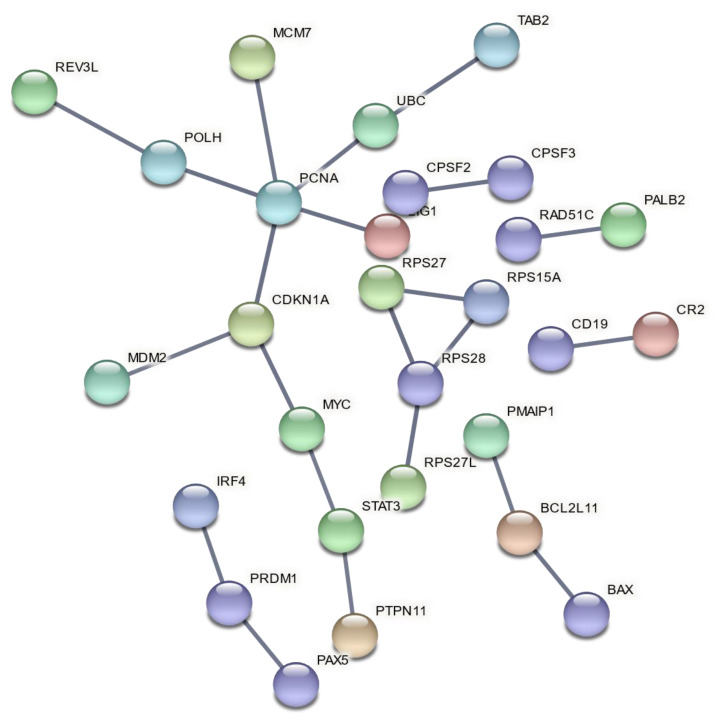
PPI network of DEGs after exposure to high doses of X-rays, collected 8–24 h post-irradiation.

**Figure 5:**
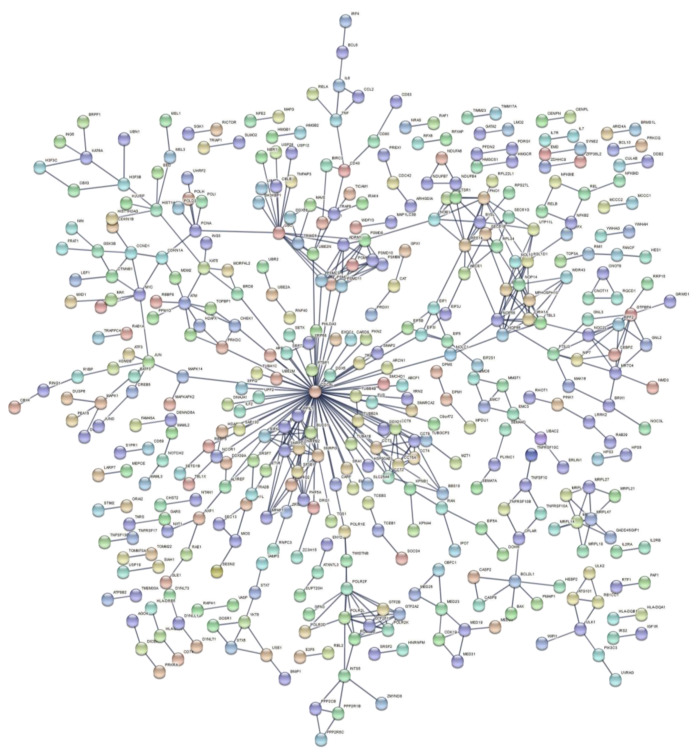
PPI network of DEGs after exposure to high doses of γ-rays, collected 1–2 h post-irradiation.

**Figure 6:**
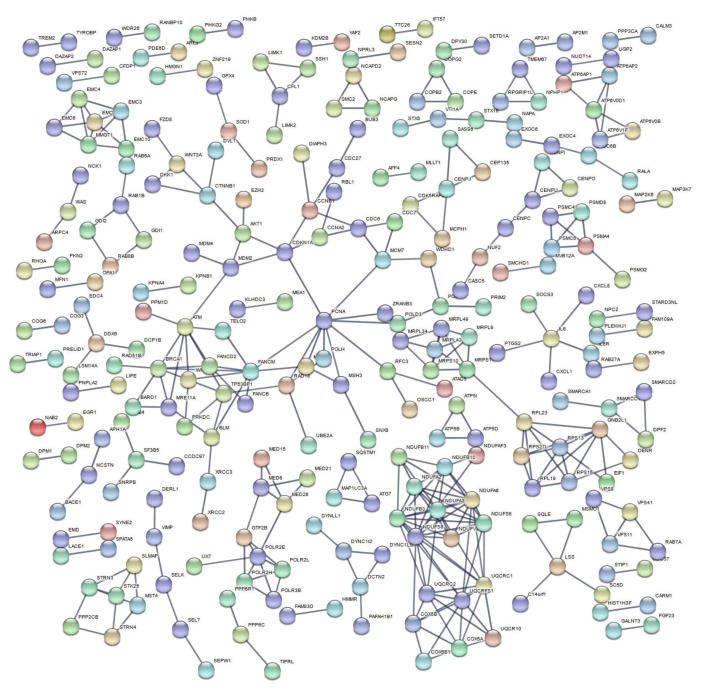
PPI network of DEGs after exposure to high doses of HZE particles, collected 6–24 h post-irradiation.

Functional enrichment analysis of up-and downregulated genes resulting from the three meta-analyses produced lists of statistically significant GO biological processes and biological pathways with a selected FDR cutoff of 0.01 (Supplementary Materials, Tables S2–S4). The enriched gene sets exhibit a level of repeatability due to the selection of reference gene sets from multiple databases, as well as the variety of gene sets that indicate the same group of biological processes or pathways. Thus, we selected the gene sets with the lowest FDR value among biologically related processes and pathways to be presented (Table 5, Table 6 and Table 7) in order to avoid redundant enriched terms. PPI networks of DEGs for each meta-analysis group were also constructed (Figure 4, Figure 5 and Figure 6).

## 4. Discussion

After we formed the three meta-analysis groups depending on the radiation type and time of collection post-irradiation (Table 1), differences between high- and low-LET effects emerged. In addition, by performing separate functional enrichment analyses for over- and under-expressed genes, a clear overview of which biological processes and pathways were up- or downregulated in each case was achieved. From the Venn diagrams created for over- and under-expressed genes post-meta-analysis (Figure 3), it is evident that the major alterations in gene expression were observed after HZE exposure (8–24 h post-irradiation), with the under-expressed genes being of greater significance regarding cellular response processes and pathways, as was later observed in the enrichment results (Table 6). DEG numbers per group are indicative of different expression patterns after exposure to high- and low-LET radiation (Figure 3), while unique DEGs after HZE exposure may suggest a response signature (Supplementary Materials, Table S1).

During the early hours (1–2) post-γ-ray exposure (Table 5), we can distinguish the biologically expected activation of DDR cumulatively through cell cycle checkpoints, p53 and apoptotic pathways, the latter being further supported by fibroblast growth factor receptor (FGFR), Forkhead box transcription factor (FoxO) and epidermal growth factor (EGF)receptor signaling pathways [77,78,79], in accordance with the results from the GSE63952 dataset [75]. Furthermore, there are multiple enriched pathways and processes that suggest immune response activation, for instance: leukocyte proliferation, cytokine signaling and NF-κB-related pathways. In addition, the interleukin 18 (IL-18) signaling pathway was triggered, which correlated with the activation of NF-κB, culminating in the production and release of several cytokines, chemokines and cellular adhesion molecules, as well as mitogen-activated protein kinases (MAPKs) activation. IL-18-mediated signaling acts as one of the vital components of the immunomodulatory cytokine networks involved in host defense, inflammation and tissue regeneration [80]. In addition, inflammatory bowel disease (IBD) is over-represented, probably due to DEGs associated with the innate immune response [81]. Finally, adaptive immune system mechanisms were represented through antigen processing and presentation, along with allograft rejection pathways [82]. To conclude for the early response to γ-rays, several disease pathways were also stimulated, like Type I diabetes mellitus and rheumatoid arthritis. However, links to disease formation also arose from under-expressed DEGs (Table 5). Inhibition of platelet-derived growth factor (PDGF) receptor signaling resulted in the loss of pericytes and a reduction in vessel density in the neovascularized cornea involved in the pathogenesis of corneal neovascularization. Additionally, PDGF plays a prominent role in the migration of smooth muscle cells (SMCs) into the neointima in atherosclerosis and is also involved in the formation of cardiovascular disease [83,84]. Furthermore, insufficient ErbB protein family signaling in humans is associated with the development of neurodegenerative diseases, such as multiple sclerosis (MS) and Alzheimer’s disease [85]. Finally, a contradicting result is that autophagy mechanisms seem to be deactivated, as autophagy ensures cell homeostasis by degrading damaged organelles and proteins, and by simultaneously providing energy under stressful conditions such as radiation exposure [86]. Nevertheless, this result, together with the DNA damage response (*ATM*-dependent only) under-regulation may be an artifact of ORA enrichment method in combination with the early 1–2 h time point.

For HZE particle exposure 8–24 post-irradiation (Table 6), the most interesting finding is the variety of DNA repair mechanism that are halted, as observed from the enrichment results for under-expressed DEGs. These mainly include homologous recombination and DSB repair. In addition, the presence of single strand annealing (SSA) supports the overflow of DSBs that occur after high-LET exposure and correlates with the down-regulated chromosome organization pathways. However, while SSA is capable of restoring a broken chromosome to avoid its loss during cell division, overreliance on SSA could be catastrophic to mammalian genomes, given the high density of repetitive elements [87], thus promoting the mutagenic effect of such types of IR. To conclude, the above, along with several downregulated pathways and processes regarding the cell cycle and the lack of an indication of apoptotic mechanism, may suggest that the cell ceases efforts to repair DNA lesions after 8 h post-irradiation.

Regarding upregulated enriched terms, neutrophils display an array of biological functions that are important for both the innate and adaptive immune responses. In addition, they exhibit marked abnormalities in phenotype and function in various systemic autoimmune diseases [88], while neutrophil degranulation is also an important event in inflammatory diseases such as asthma and chronic obstructive pulmonary disease (COPD) [89]. Although neutrophils are considered as one kind of phagocyte of the innate immune system, more and more evidence has supported that they can also play an important regulatory role in the adaptive immune response [90]. Additionally, murine and human studies, to date, show that they are potent modulators of immunity and are capable of directly interacting with lymphocytes and modulating their responses at local sites of inflammation, as well as in draining lymph nodes [91]. To conclude, neutrophils are rather complex cells demonstrating the capacity of modulating the adaptive immune response via direct interaction with, or by producing cytokines that affect dendritic cells and lymphocytes, while their heterogeneity, with clearly different functional phenotypes, has been recently described, particularly in cancer and inflammation [92].

Subsequently, the most upregulated pathway is that of the lysosome, probably explained by the fact that substances for digestion are acquired by the lysosomes via a series of processes including endocytosis, phagocytosis and autophagy [93]. This result suggests that autophagy mechanisms seem to persist after high-LET exposure and should be studied further concerning their effects on healthy tissue after heavy ion tumor treatments [94].

In a comparison of HZE and X-ray enrichment results for later hours of exposure (8–24), the number of enriched terms with a significant FDR is far lower in the latter case (Table 6 and Table 7). This fact is further supported by the dense PPI networks formed after HZE exposure in comparison with X-rays (Figure 4 and Figure 6). The presence of the *CDKN1A* gene is identified in all three networks (Figure 4, Figure 5 and Figure 6). This gene has also been found to be overexpressed in multiple IR-exposed human cell lines, such as a series of healthy cells after exposure to high doses of X-rays and gamma-rays [68], and healthy [95] and cancerous [96] breast epithelial cells exposed to high doses of electron radiation. The p21 protein encoded by this gene, which is known to be interdependent with tumor suppressor protein TP53 [13], is also responsible for inhibiting cellular proliferation in response to DNA damage and highly correlated with DNA repair and apoptosis [76]. This holistic bioinformatics approach revealed cellular trends towards inflammation and degeneration, which might be central to the development of the late effects of high-LET radiation exposure. Furthermore, this pattern of DNA repair mechanism arrest that is not observed after low-LET exposure might be correlated with complex DNA damage formation. This increase in complex DNA damage in comparison with X-ray exposure has been previously observed [97] and could involve the triggering of chromosomal translocations [98]. Recent reports show downregulation of cell cycle suppressing genes (*ABL1* and *CDKN1A*) and upregulation of cell cycle promoting genes (*CCNB1,* *CCND1,* *KPNA2,* *MCM4, MKI67* and *STMN1*) in cells exposed to HZE particles under microgravity [99]. HZE particles themselves induce complex DNA damage and arrest DNA repair process, and also failure to arrest at the G1/S, G2 checkpoints under microgravity; the combination of these responses will lead to genomic instability, including chromosome aberrations, and eventually lead to malignant transformation of those cells. Based on our results and all the above analyses and discussion, we believe in general that the cell’s response to IR (especially high-LET) is a complex phenomenon depending not only on RBE/LET but also on cell type, radiation dose and time of collection post-irradiation. Furthermore, the RBE differs for different biological endpoints such as cell survival, induction of chromosomal aberrations and gene expression. In addition, there is not a clear direct relationship between gene expression and RBE for cell survival, as the latter cannot be solely based on DEG counts. More accurately, RBE/LET effects should conjointly be interpreted based on the biological pathways and processes they represent, as well as the degree of association between them (as can be ascertained through PPI network construction). Nonetheless, a much higher number of DEGs for high-LET particles (x 6.8) compared with X-rays (Table 4), for the 8–24 h time frame, is detected, probably due to the different RBEs. However, that was not the case for γ-ray exposure, where we detected the number of DEGs increased by a factor of ~3.1 for the 1–2 h time frame, yet enriched terms were quite significant.

To assess the risk of radiation in future long-term space travel, current cumulative bioinformatics approaches may be extremely helpful in understanding the overall cellular responses to HZE particles under a systems biology approach. Consequently, understanding radiation-response mechanisms can aid in the development of countermeasures for future space missions and ameliorate the detrimental effects of heavy ions on astronauts while optimizing radiation therapy protocols utilizing heavy ions. Finally, the most interesting question that arises from our results is the manner through which adaptive immune mechanisms may affect the high-LET radiation response.

## Figures and Tables

**Table 1:** Information on each meta-analysis group regarding experimental conditions and datasets.

Data Series	Conditions			Meta-Analysis Group
	IR	Dose [Gy]	Time [h]	
GSE63952	γ-rays	1.5	1	γ-rays
GSE63952	γ-rays	1.5	2	
E-MTAB-5761	Carbon ions	1	8	high LET
E-MTAB-5761	Iron ions	1	8	
E-MTAB-5754	Iron ions	2	24	
E-MTAB-3463	X-rays	1	8	X-rays
E-MTAB-5754	X-rays	2	24	

**Table 2:** Repository accessions and experimental description of selected samples.

Accession and Platform	IR Type	LET	Human Tissue	Condition	Sample Count
**GSE63952** [PrimeView] Affymetrix Human Gene Expression Array-GPL15207	γ-rays (Cs-137)	0.91 keV/µm	Isolated leucocytes (irradiation of whole blood)	Control-1 h	10
				1.5 Gy-1 h	10
				Control-2 h	10
				1.5 Gy|2 h	10
**E-MTAB-5754** [HuGene-2_0-st] Affymetrix Human Gene 2.0 ST Array-GPL16686	X-rays	3 keV/µm	Coronary artery endothelial cells	Control	3
				2 Gy-24 h	3
	Fe ions	155 keV/µm		Control	3
				2 Gy-24 h	3
**E-MTAB-3463** **E-MTAB-5761** [HuGene-1_0-st-v1] Affymetrix GeneChip Human Gene 1.0 ST Array-A-AFFY-141	X-rays	2–3 keV/µm	Peripheral blood mononuclear cells	Control	20
				1 Gy-8 h	20
	Fe ions	155 keV/µm		Control	4
				1 Gy-8 h	4
	C Ions	60–80 keV/µm		Control	4
				1 Gy-8 h	4

**Table 3:** DEG counts derived from DGEA for each comparison within each dataset. The table includes information about the experimental sample parameters and total mapped gene count for every Affymetrix microarray.

Accession	E-MTAB-5754		E-MTAB-5761|3463			GSE63952	
Mapped Genes	23195		19758|19211			14383	
IR Type	Fe Ions	X-rays	Fe Ions	C Ions	X-rays	γ-rays	
Dose	2 Gy	2 Gy	1 Gy	1 Gy	1 Gy	1.5 Gy	
Time Point	24 h	24 h	8 h	8 h	8 h	2 h	6 h
DEG Counts	3661	6296	7487	6760	412	2470	704

**Table 4:** Description of experimental conditions for each meta-analysis group and final DEG counts. Common DEG counts obtained from the initial DGEA are also included for comparison. A much higher number of DEGs for with high-linear energy transfer (LET) radiation compared with X-rays (factor ~ 6.8) and γ-rays (factor ~ 3.1) is observed.

Group	γ-rays		High LET		X-rays	
IR	γ-rays		Fe|Carbon Ions		X-rays	
Dose	1.5 Gy		1–2 Gy		1–2 Gy	
Time	1–2 h		6–24 h		8–24 h	
Expression	Over	Under	Over	Under	Over	Under
Final DEG count	743	720	2689	1839	434	226
Final Total DEGS	1463		4528		660	
Pre-meta DEGs	248		1105		146	
Mapped Genes	14383		26747		26179	

**Table 5:** Significantly enriched biological processes and pathways for up- and downregulated genes after exposure to high doses of γ-rays, collected 1–2 h post-irradiation.

Gene Set	Description	FDR
Upregulated genes ↑		
GO:0022613	Ribonucleoprotein complex biogenesis	0
GO:0016072	rRNA metabolic process	0.000000301
GO:0072331	Signal transduction by p53 class mediator	0.000012800
GO:0042770	Signal transduction in response to DNA damage	0.000020800
GO:2001233	Regulation of apoptotic signaling pathway	0.001434038
GO:0097193	Intrinsic apoptotic signaling pathway	0.002302183
GO:0070661	Leukocyte proliferation	0.003108069
GO:0071900	Regulation of protein serine/threonine kinase activity	0.005804333
GO:0045930	Negative regulation of mitotic cell cycle	0.00809352
GO:0009314	Response to radiation	0.009151245
R-HSA-8953854	Metabolism of RNA	0.000000000
WP4286	Genotoxicity pathway	0.000000095
R-HSA-3700989	Transcriptional regulation by TP53	0.000001200
hsa04115	p53 signaling pathway	0.000004120
WP1530	miRNA regulation of DNA damage response	0.000037400
R-HSA-1280215	Cytokine signaling in immune system	0.000387000
WP707	DNA damage response	0.000473000
R-HSA-190236	Signaling by FGFR	0.000802000
hsa05166	Human T cell leukemia virus 1 infection	0.001199481
R-HSA-5668541	TNFR2 non-canonical NF-kB pathway	0.001199481
R-HSA-2555396	Mitotic metaphase and anaphase	0.001231855
hsa05330	Allograft rejection	0.001496383
WP3617	Photodynamic therapy-induced NF-κB survival signaling	0.001496383
WP254	Apoptosis	0.001564745
R-HSA-8852276	The role of GTSE1 in G2/M progression after G2 checkpoint	0.001630981
hsa04940	Type I diabetes mellitus	0.00261137
WP231	TNF alpha signaling pathway	0.003514333
WP4754	IL-18 signaling pathway	0.004141241
R-HSA-5357801	Programmed cell death	0.004562085
R-HSA-202424	Downstream TCR signaling	0.004973236
hsa05321	Inflammatory bowel disease (IBD)	0.005425333
hsa04612	Antigen processing and presentation	0.007296195
R-HSA-5675221	Negative regulation of MAPK pathway	0.007296195
hsa05416	Viral myocarditis	0.009034727
hsa04640	Hematopoietic cell lineage	0.009151245
Downregulated genes ↓		
hsa04068	FoxO signaling pathway	0.000580000
WP710	DNA damage response (only ATM-dependent)	0.002169335
P00047	PDGF signaling pathway	0.002446475
hsa04140	Autophagy	0.004449471
P00018	EGF receptor signaling pathway	0.007375195
hsa04012	ErbB signaling pathway	0.009145883

**Table 6:** Significant enriched biological processes and pathways for up-and downregulated genes after exposure to high doses of high Z and high energy (HZE), collected 6–24 h post-irradiation.

Gene set	Description	FDR
Upregulated genes ↑		
GO:0002446	Neutrophil mediated immunity	0.001203968
GO:0036230	Granulocyte activation	0.001389131
hsa04142	Lysosome	0.000000170
R-HSA-6798695	Neutrophil degranulation	0.000399000
WP4286	Genotoxicity pathway	0.001389131
hsa00600	Sphingolipid metabolism	0.009444878
Downregulated genes ↓		
GO:0007059	Chromosome segregation	0
GO:0071103	DNA conformation change	0.000000000
GO:1901987	Regulation of cell cycle phase transition	0.000000034
GO:0044839	Cell cycle G2/M phase transition	0.000000083
GO:0033044	Regulation of chromosome organization	0.000001350
GO:0006302	Double-strand break repair	0.000003850
GO:0006310	DNA recombination	0.000007580
GO:0045930	Negative regulation of mitotic cell cycle	0.000077000
GO:0007051	Spindle organization	0.000092300
GO:0006338	Chromatin remodeling	0.000148000
R-HSA-1640170	Cell cycle	0
R-HSA-68877	Mitotic prometaphase	0
R-HSA-68886	M phase	0.000000001
R-HSA-69620	Cell cycle checkpoints	0.000000001
R-HSA-2500257	Resolution of sister chromatid cohesion	0.000000024
R-HSA-69618	Mitotic spindle checkpoint	0.000000071
R-HSA-2565942	Regulation of PLK1 activity at G2/M transition	0.000004010
R-HSA-380287	Centrosome maturation	0.000009780
R-HSA-2467813	Separation of sister chromatids	0.000013200
R-HSA-73894	DNA repair	0.000014000
R-HSA-8854518	AURKA activation by TPX2	0.000018300
R-HSA-69275	G2/M transition	0.000058500
R-HSA-5693579	Homologous DNA pairing and strand exchange	0.000106000
WP4016	DNA IR-damage and cellular response via ATR	0.000111000
hsa03440	Homologous recombination	0.000946000
R-HSA-5693607	Processing of DNA double-strand break ends	0.001067765
R-HSA-5693567	HDR through homologous recombination (HRR) or single strand annealing (SSA)	0.001665003
R-HSA-983168	Antigen processing: ubiquitination and proteasome degradation	0.003072282
R-HSA-69473	G2/M DNA damage checkpoint	0.004395125
R-HSA-5693532	DNA double-strand break repair	0.005140536

**Table 7:** Significantly enriched biological processes and pathways for upregulated genes after exposure to high doses of X-rays, collected 8–24 h post-irradiation.

Gene Set	Description	FDR
Upregulated genes ↑		
GO:0072331	Signal transduction by p53 class mediator	0
GO:0042770	Signal transduction in response to DNA damage	0.000002070
GO:0000075	Cell cycle checkpoint	0.000509000
GO:0009314	Response to radiation	0.000623000
GO:0007050	Cell cycle arrest	0.00594055
GO:0097193	Intrinsic apoptotic signaling pathway	0.006625582
WP4286	Genotoxicity pathway	0
R-HSA-73857	RNA polymerase II transcription	0
R-HSA-3700989	Transcriptional regulation by TP53	0
hsa04115	p53 signaling pathway	0.000000001
WP1530	miRNA regulation of DNA damage response	0.000002070
WP707	DNA damage response	0.000010900
R-HSA-6791312	TP53 regulates transcription of cell cycle genes	0.000137000
R-HSA-5633008	TP53 regulates transcription of cell death genes	0.000691000
hsa04068	FoxO signaling pathway	0.002554446

## Data Availability

All primary omics datasets used and analysis results are included in the manuscript but available also on logical request.

## Supplementary Materials

Supplementary Materials can be found at https://www.mdpi.com/2075-1729/11/2/115/s1, Table S1: Statistically significant DEGs (Adj. *p*-value < 0.01) derived from meta-analysis for samples irradiated with high doses of HZE particles, collected 6–24 h post-IR not common with any other meta-analysis group. This meta-analysis group consists of 3 DEG lists obtained from DGEA, using a total of 11 control and 11 irradiated samples [Data Series: E-MTAB-5761 and E-MTAB-5754], Table S2: Enriched biological processes and pathways for up- and down-regulated genes after exposure to high doses of γ-rays, collected 1–2 h post irradiation, Table S3: Enriched biological processes and pathways for up- and down-regulated genes after exposure to high doses of HZE, collected 6–24 h post irradiation, Table S4: Enriched biological processes and pathways for up-regulated genes after exposure to high doses of X-rays, collected 8–24 h post irradiation.

## Abbreviations

CDK	Cyclic-Dependent Kinase
CNS	Central Nervous System
COPD	Chronic Obstructive Pulmonary Disease
DDR	DNA Damage Response
DEGs	Differentially Expressed Genes
DGEA	Differential Gene Expression Analysis
DSBs	Double Strand Breaks
FDR	False Discovery Rate
EGF	Epidermal Growth Factor
FGFR	Fibroblast Growth Factor Receptor
FoxO	Forkhead box transcription factors
GCR	Galactic Cosmic Radiation
GEO	Gene Expression Omnibus
GO	Gene Ontology
HR	Homologous Recombination
HZE	High Z and high Energy particles
IBD	Inflammatory Bowel Disease
IgA	Immunoglobulin A
IL-18	Interleukin 18
LET	Linear Energy Transfer
Log_2_FC	Log_2_ Fold Change
MAPKs	Mitogen-Activated Protein Kinases
MDS	Multidimensional Scaling
MS	Multiple Sclerosis
NF-κB	Nuclear Factor kappa B
ORA	Over-Representation Analysis
PCA	Principal Component Analysis
PDGF	Platelet-Derived Growth Factor
PPI	Protein–Protein Interaction
RBE	Relative Biological Effectiveness
RMA	Robust Multi-array Average
ROS	Reactive Oxygen Species
SMC	Smooth Muscle Cells
SSA	Single Strand Annealing

## Appendix A

**Table A1:** Specific arguments used during the analysis. Unless explicitly specified otherwise, default parameters were used.

**WebGestalt**	**Basic Parameters:**
	Organism of interest: *Homo sapiens* Method of interest: ORA Functional database: Gene Ontology + (biological process: no redundant), pathway + (KEGG)(Reactome)(Panther)(WikiPathways), Network + (Transcription Factor target) Gene list: Select Gene ID type: Ensembl gene id Reference gene list: Upload user reference set: (total mappings–Ensembl gene id)
	**Advanced Parameters**
	Minimum number of genes for category: 2 Multiple test adjustment: BH (Benjamini–Hochberg) Significance level: FDR (0.05) Number of categories visualized in the report: 100
**STRING**	**Basic Settings:**
	Meaning of network edges: confidence Active interaction sources: text mining, experiments Minimum required interaction score: highest confidence (0.9)
	**Advanced Settings:**
	Hide disconnected nodes in the network Disable structure previous inside network bubbles
