# Supplementary material for: A Meta-Analysis of the Effects of High-LET Ionizing Radiations in Human Gene Expression
Source: Life (Basel). 2021 Feb 3;11(2):115. doi: 10.3390/life11020115 (PMC7913660; doi:10.3390/life11020115)
Supplement: Supplementary file 1 [file life-11-00115-s001.pdf]

life

MDPI

Supplementary Materials

A Meta-Analysis of the Effects of High-LET Ionizing Radiations in Human Gene Expression

Table S1. Statistically significant DEGs (Adj. *p*-value < 0.01) derived from meta-analysis for samples irradiated with high doses of HZE particles, collected 6-24 h post-IR not common with any other meta-analysis group. This meta-analysis group consists of 3 DEG lists obtained from DGEA, using a total of 11 control and 11 irradiated samples [Data Series: E-MTAB-5761 and E-MTAB-5754].

Ensembl ID	Gene Symbol	Gene Description
**Up-Regulated Genes ↑ (2425)**		
ENSG00000000938	FGR	FGR proto-oncogene, Src family tyrosine kinase
ENSG00000001036	FUCA2	alpha-L-fucosidase 2
ENSG00000001084	GCLC	glutamate-cysteine ligase catalytic subunit
ENSG00000001631	KRIT1	KRIT1 ankyrin repeat containing
ENSG00000002079	MYH16	myosin heavy chain 16 pseudogene
ENSG00000002587	HS3ST1	heparan sulfate-glucosamine 3-sulfotransferase 1
ENSG00000003056	M6PR	mannose-6-phosphate receptor, cation dependent
ENSG00000004059	ARF5	ADP ribosylation factor 5
ENSG00000004777	ARHGAP33	Rho GTPase activating protein 33
ENSG00000004799	PDK4	pyruvate dehydrogenase kinase 4
ENSG00000004848	ARX	aristaless related homeobox
ENSG00000005022	SLC25A5	solute carrier family 25 member 5
ENSG00000005108	THSD7A	thrombospondin type 1 domain containing 7A
ENSG00000005194	CIAPIN1	cytokine induced apoptosis inhibitor 1
ENSG00000005381	MPO	myeloperoxidase
ENSG00000005486	RHBDD2	rhomboid domain containing 2
ENSG00000005884	ITGA3	integrin subunit alpha 3
ENSG00000006016	CRLF1	cytokine receptor like factor 1
ENSG00000006377	DLX6	distal-less homeobox 6
ENSG00000006451	RALA	RAS like proto-oncogene A
ENSG00000006704	GTF2IRD1	GTF2I repeat domain containing 1
ENSG00000007047	MARK4	microtubule affinity regulating kinase 4
ENSG00000007174	DNAH9	dynein axonemal heavy chain 9
ENSG00000008517	IL32	interleukin 32
ENSG00000008710	PKD1	polycystin 1, transient receptor potential channel interacting
ENSG00000009765	IYD	iodotyrosine deiodinase
ENSG00000010256	UQCRC1	ubiquinol-cytochrome c reductase core protein 1
ENSG00000010270	STARD3NL	STARD3 N-terminal like
ENSG00000010295	IFFO1	intermediate filament family orphan 1
ENSG00000010319	SEMA3G	semaphorin 3G

ENSG00000010322	NISCH	nischarin
ENSG00000010327	STAB1	stabilin 1
ENSG00000010610	CD4	CD4 molecule
ENSG00000010704	HFE	homeostatic iron regulator
ENSG00000011523	CEP68	centrosomal protein 68
ENSG00000011566	MAP4K3	mitogen-activated protein kinase kinase kinase kinase 3
ENSG00000011590	ZBTB32	zinc finger and BTB domain containing 32
ENSG00000011600	TYROBP	transmembrane immune signaling adaptor TYROBP
ENSG00000012822	CALCOCO1	calcium binding and coiled-coil domain 1
ENSG00000013288	MAN2B2	mannosidase alpha class 2B member 2
ENSG00000013588	GPRC5A	G protein-coupled receptor class C group 5 member A
ENSG00000014164	ZC3H3	zinc finger CCCH-type containing 3
ENSG00000014216	CAPN1	calpain 1
ENSG00000015285	WAS	WASP actin nucleation promoting factor
ENSG00000015413	DPEP1	dipeptidase 1
ENSG00000015475	BID	BH3 interacting domain death agonist
ENSG00000020219	CCT8L1P	chaperonin containing TCP1 subunit 8 like 1, pseudogene
ENSG00000023318	ERP44	endoplasmic reticulum protein 44
ENSG00000023902	PLEKHO1	pleckstrin homology domain containing O1
ENSG00000027697	IFNGR1	interferon gamma receptor 1
ENSG00000030582	GRN	granulin precursor
ENSG00000031823	RANBP3	RAN binding protein 3
ENSG00000032444	PNPLA6	patatin like phospholipase domain containing 6
ENSG00000033627	ATP6V0A1	ATPase H+ transporting V0 subunit a1
ENSG00000034713	GABARAPL2	GABA type A receptor associated protein like 2
ENSG00000036672	USP2	ubiquitin specific peptidase 2
ENSG00000037757	MRI1	methylthioribose-1-phosphate isomerase 1
ENSG00000038358	EDC4	enhancer of mRNA decapping 4
ENSG00000038945	MSR1	macrophage scavenger receptor 1
ENSG00000040531	CTNS	cystinosin, lysosomal cystine transporter
ENSG00000040633	PHF23	PHD finger protein 23
ENSG00000041357	PSMA4	proteasome 20S subunit alpha 4
ENSG00000042062	RIPOR3	RIPOR family member 3
ENSG00000042753	AP2S1	adaptor related protein complex 2 subunit sigma 1
ENSG00000043591	ADRB1	adrenoceptor beta 1
ENSG00000044012	GUCA2B	guanylate cyclase activator 2B
ENSG00000047648	ARHGAP6	Rho GTPase activating protein 6
ENSG00000049089	COL9A2	collagen type IX alpha 2 chain
ENSG00000049130	KITLG	KIT ligand
ENSG00000049323	LTBP1	latent transforming growth factor beta binding protein 1
ENSG00000049860	HEXB	hexosaminidase subunit beta
ENSG00000050767	COL23A1	collagen type XXIII alpha 1 chain
ENSG00000050820	BCAR1	BCAR1 scaffold protein, Cas family member

ENSG00000052802	MSMO1	methylsterol monooxygenase 1
ENSG00000052850	ALX4	ALX homeobox 4
ENSG00000053918	KCNQ1	potassium voltage-gated channel subfamily Q member 1
ENSG00000054116	TRAPPC3	trafficking protein particle complex 3
ENSG00000055950	MRPL43	mitochondrial ribosomal protein L43
ENSG00000057608	GDI2	GDP dissociation inhibitor 2
ENSG00000058262	SEC61A1	SEC61 translocon subunit alpha 1
ENSG00000058453	CROCC	ciliary rootlet coiled-coil, rootletin
ENSG00000059122	FLYWCH1	FLYWCH-type zinc finger 1
ENSG00000061656	SPAG4	sperm associated antigen 4
ENSG00000062598	ELMO2	engulfment and cell motility 2
ENSG00000063176	SPHK2	sphingosine kinase 2
ENSG00000063180	CA11	carbonic anhydrase 11
ENSG00000063244	U2AF2	U2 small nuclear RNA auxiliary factor 2
ENSG00000063515	GSC2	goosecoid homeobox 2
ENSG00000063660	GPC1	glypican 1
ENSG00000063854	HAGH	hydroxyacylglutathione hydrolase
ENSG00000064195	DLX3	distal-less homeobox 3
ENSG00000064545	TMEM161A	transmembrane protein 161A
ENSG00000064601	CTSA	cathepsin A
ENSG00000065320	NTN1	netrin 1
ENSG00000065518	NDUFB4	NADH:ubiquinone oxidoreductase subunit B4
ENSG00000065833	ME1	malic enzyme 1
ENSG00000065970	FOXJ2	forkhead box J2
ENSG00000066379	POLR1H	RNA polymerase I subunit H
ENSG00000066735	KIF26A	kinesin family member 26A
ENSG00000067182	TNFRSF1A	TNF receptor superfamily member 1A
ENSG00000067225	PKM	pyruvate kinase M1/2
ENSG00000067560	RHOA	ras homolog family member A
ENSG00000067829	IDH3G	isocitrate dehydrogenase (NAD(+)) 3 non-catalytic subunit gamma
ENSG00000068078	FGFR3	fibroblast growth factor receptor 3
ENSG00000068438	FTSJ1	FtsJ RNA 2'-O-methyltransferase 1
ENSG00000068697	LAPTM4A	lysosomal protein transmembrane 4 alpha
ENSG00000068903	SIRT2	sirtuin 2
ENSG00000069011	PITX1	paired like homeodomain 1
ENSG00000069329	VPS35	VPS35 retromer complex component
ENSG00000069399	BCL3	BCL3 transcription coactivator
ENSG00000069424	KCNAB2	potassium voltage-gated channel subfamily A regulatory beta subunit 2
ENSG00000069482	GAL	galanin and GMAP prepropeptide
ENSG00000069696	DRD4	dopamine receptor D4
ENSG00000070444	MNT	MAX network transcriptional repressor
ENSG00000070729	CNGB1	cyclic nucleotide gated channel subunit beta 1

ENSG00000070761	CFAP20	cilia and flagella associated protein 20
ENSG00000070814	TCOF1	treacle ribosome biogenesis factor 1
ENSG00000070985	TRPM5	transient receptor potential cation channel subfamily M member 5
ENSG00000071205	ARHGAP10	Rho GTPase activating protein 10
ENSG00000071553	ATP6AP1	ATPase H+ transporting accessory protein 1
ENSG00000071626	DAZAP1	DAZ associated protein 1
ENSG00000071655	MBD3	methyl-CpG binding domain protein 3
ENSG00000071677	PRLH	prolactin releasing hormone
ENSG00000071859	FAM50A	family with sequence similarity 50 member A
ENSG00000071889	FAM3A	FAM3 metabolism regulating signaling molecule A
ENSG00000072062	PRKACA	protein kinase cAMP-activated catalytic subunit alpha
ENSG00000072071	ADGRL1	adhesion G protein-coupled receptor L1
ENSG00000072195	SPEG	striated muscle enriched protein kinase
ENSG00000072422	RHOBTB1	Rho related BTB domain containing 1
ENSG00000072518	MARK2	microtubule affinity regulating kinase 2
ENSG00000073598	FNDC8	fibronectin type III domain containing 8
ENSG00000073792	IGF2BP2	insulin like growth factor 2 mRNA binding protein 2
ENSG00000074621	SLC24A1	solute carrier family 24 member 1
ENSG00000075240	GRAMD4	GRAM domain containing 4
ENSG00000075391	RASAL2	RAS protein activator like 2
ENSG00000075415	SLC25A3	solute carrier family 25 member 3
ENSG00000075461	CACNG4	calcium voltage-gated channel auxiliary subunit gamma 4
ENSG00000075785	RAB7A	RAB7A, member RAS oncogene family
ENSG00000075886	TUBA3D	tubulin alpha 3d
ENSG00000076201	PTPN23	protein tyrosine phosphatase non-receptor type 23
ENSG00000076356	PLXNA2	plexin A2
ENSG00000076944	STXBP2	syntaxin binding protein 2
ENSG00000077009	NMRK2	nicotinamide riboside kinase 2
ENSG00000077080	ACTL6B	actin like 6B
ENSG00000077463	SIRT6	sirtuin 6
ENSG00000077514	POLD3	DNA polymerase delta 3, accessory subunit
ENSG00000078808	SDF4	stromal cell derived factor 4
ENSG00000078814	MYH7B	myosin heavy chain 7B
ENSG00000078902	TOLLIP	toll interacting protein
ENSG00000079277	MKNK1	MAPK interacting serine/threonine kinase 1
ENSG00000079313	REXO1	RNA exonuclease 1 homolog
ENSG00000079385	CEACAM1	CEA cell adhesion molecule 1
ENSG00000079432	CIC	capicua transcriptional repressor
ENSG00000079435	LIPE	lipase E, hormone sensitive type
ENSG00000079459	FDFT1	farnesyl-diphosphate farnesyltransferase 1
ENSG00000079805	DNM2	dynamin 2
ENSG00000080573	COL5A3	collagen type V alpha 3 chain
ENSG00000082929	LINC01587	long intergenic non-protein coding RNA 1587

ENSG00000083444	PLOD1	procollagen-lysine,2-oxoglutarate 5-dioxygenase 1
ENSG00000084234	APLP2	amyloid beta precursor like protein 2
ENSG00000084628	NKAIN1	sodium/potassium transporting ATPase interacting 1
ENSG00000084636	COL16A1	collagen type XVI alpha 1 chain
ENSG00000084764	MAPRE3	microtubule associated protein RP/EB family member 3
ENSG00000085265	FCN1	ficolin 1
ENSG00000085644	ZNF213	zinc finger protein 213
ENSG00000085733	CTTN	cortactin
ENSG00000086506	HBQ1	hemoglobin subunit theta 1
ENSG00000087076	HSD17B14	hydroxysteroid 17-beta dehydrogenase 14
ENSG00000087077	TRIP6	thyroid hormone receptor interactor 6
ENSG00000087116	ADAMTS2	ADAM metallopeptidase with thrombospondin type 1 motif 2
ENSG00000087250	MT3	metallothionein 3
ENSG00000087253	LPCAT2	lysophosphatidylcholine acyltransferase 2
ENSG00000087495	PHACTR3	phosphatase and actin regulator 3
ENSG00000088038	CNOT3	CCR4-NOT transcription complex subunit 3
ENSG00000088256	GNA11	G protein subunit alpha 11
ENSG00000088854	C20orf194	chromosome 20 open reading frame 194
ENSG00000089050	RBBP9	RB binding protein 9, serine hydrolase
ENSG00000089057	SLC23A2	solute carrier family 23 member 2
ENSG00000089063	TMEM230	transmembrane protein 230
ENSG00000089225	TBX5	T-box transcription factor 5
ENSG00000089289	IGBP1	immunoglobulin binding protein 1
ENSG00000089486	CDIP1	cell death inducing p53 target 1
ENSG00000089597	GANAB	glucosidase II alpha subunit
ENSG00000090013	BLVRB	biliverdin reductase B
ENSG00000090061	CCNK	cyclin K
ENSG00000090372	STRN4	striatin 4
ENSG00000090432	MUL1	mitochondrial E3 ubiquitin protein ligase 1
ENSG00000090565	RAB11FIP3	RAB11 family interacting protein 3
ENSG00000090581	GNPTG	N-acetylglucosamine-1-phosphate transferase subunit gamma
ENSG00000090661	CERS4	ceramide synthase 4
ENSG00000090674	MCOLN1	mucolipin 1
ENSG00000091536	MYO15A	myosin XVA
ENSG00000092010	PSME1	proteasome activator subunit 1
ENSG00000092850	TEKT2	tektin 2
ENSG00000093010	COMT	catechol-O-methyltransferase
ENSG00000095321	CRAT	carnitine O-acetyltransferase
ENSG00000095397	WHRN	whirlin
ENSG00000095713	CRTAC1	cartilage acidic protein 1
ENSG00000095917	TPSD1	tryptase delta 1
ENSG00000095970	TREM2	triggering receptor expressed on myeloid cells 2
ENSG00000096080	MRPS18A	mitochondrial ribosomal protein S18A

ENSG00000099194	SCD	stearoyl-CoA desaturase
ENSG00000099377	HSD3B7	hydroxy-delta-5-steroid dehydrogenase, 3 beta- and steroid delta-isomerase 7
ENSG00000099381	SETD1A	SET domain containing 1A, histone lysine methyltransferase
ENSG00000099622	CIRBP	cold inducible RNA binding protein
ENSG00000099624	ATP5F1D	ATP synthase F1 subunit delta
ENSG00000099625	CBARP	CACN subunit beta associated regulatory protein
ENSG00000099785	MARCHF2	membrane associated ring-CH-type finger 2
ENSG00000099797	TECR	trans-2,3-enoyl-CoA reductase
ENSG00000099814	CEP170B	centrosomal protein 170B
ENSG00000099817	POLR2E	RNA polymerase II, I and III subunit E
ENSG00000099840	IZUMO4	IZUMO family member 4
ENSG00000099901	RANBP1	RAN binding protein 1
ENSG00000099917	MED15	mediator complex subunit 15
ENSG00000099954	CECR2	CECR2 histone acetyl-lysine reader
ENSG00000099992	TBC1D10A	TBC1 domain family member 10A
ENSG00000099995	SF3A1	splicing factor 3a subunit 1
ENSG00000100038	TOP3B	DNA topoisomerase III beta
ENSG00000100055	CYTH4	cytohesin 4
ENSG00000100083	GGA1	golgi associated, gamma adaptin ear containing, ARF binding protein 1
ENSG00000100106	TRIOBP	TRIO and F-actin binding protein
ENSG00000100122	CRYBB1	crystallin beta B1
ENSG00000100225	FBXO7	F-box protein 7
ENSG00000100226	GTPBP1	GTP binding protein 1
ENSG00000100234	TIMP3	TIMP metallopeptidase inhibitor 3
ENSG00000100253	MIOX	myo-inositol oxygenase
ENSG00000100258	LMF2	lipase maturation factor 2
ENSG00000100263	RHBDD3	rhomboid domain containing 3
ENSG00000100292	HMOX1	heme oxygenase 1
ENSG00000100299	ARSA	arylsulfatase A
ENSG00000100302	RASD2	RASD family member 2
ENSG00000100311	PDGFB	platelet derived growth factor subunit B
ENSG00000100346	CACNA1I	calcium voltage-gated channel subunit alpha1 I
ENSG00000100365	NCF4	neutrophil cytosolic factor 4
ENSG00000100380	ST13	ST13 Hsp70 interacting protein
ENSG00000100401	RANGAP1	Ran GTPase activating protein 1
ENSG00000100600	LGMN	legumain
ENSG00000100625	SIX4	SIX homeobox 4
ENSG00000100726	TELO2	telomere maintenance 2
ENSG00000100823	APEX1	apurinic/apyrimidinic endodeoxyribonuclease 1
ENSG00000100884	CPNE6	copine 6
ENSG00000100938	GMPR2	guanosine monophosphate reductase 2
ENSG00000100949	RABGGTA	Rab geranylgeranyltransferase subunit alpha

ENSG00000100987	VSX1	visual system homeobox 1
ENSG00000100997	ABHD12	abhydrolase domain containing 12, lysophospholipase
ENSG00000101076	HNF4A	hepatocyte nuclear factor 4 alpha
ENSG00000101144	BMP7	bone morphogenetic protein 7
ENSG00000101162	TUBB1	tubulin beta 1 class VI
ENSG00000101200	AVP	arginine vasopressin
ENSG00000101203	COL20A1	collagen type XX alpha 1 chain
ENSG00000101210	EEF1A2	eukaryotic translation elongation factor 1 alpha 2
ENSG00000101282	RSPO4	R-spondin 4
ENSG00000101347	SAMHD1	SAM and HD domain containing deoxynucleoside triphosphate triphosphohydrolase 1
ENSG00000101363	MANBAL	mannosidase beta like
ENSG00000101365	IDH3B	isocitrate dehydrogenase (NAD(+)) 3 non-catalytic subunit beta
ENSG00000101439	CST3	cystatin C
ENSG00000101489	CELF4	CUGBP Elav-like family member 4
ENSG00000101812	H2BW2	H2B.W histone 2
ENSG00000101825	MXRA5	matrix remodeling associated 5
ENSG00000101945	SUV39H1	suppressor of variegation 3-9 homolog 1
ENSG00000102055	PPP1R2C	PPP1R2C family member C
ENSG00000102100	SLC35A2	solute carrier family 35 member A2
ENSG00000102109	PCSK1N	proprotein convertase subtilisin/kexin type 1 inhibitor
ENSG00000102265	TIMP1	TIMP metallopeptidase inhibitor 1
ENSG00000102302	FGD1	FYVE, RhoGEF and PH domain containing 1
ENSG00000102393	GLA	galactosidase alpha
ENSG00000102539	MLNR	motilin receptor
ENSG00000102683	SGCG	sarcoglycan gamma
ENSG00000102805	CLN5	CLN5 intracellular trafficking protein
ENSG00000102870	ZNF629	zinc finger protein 629
ENSG00000102901	CENPT	centromere protein T
ENSG00000102924	CBLN1	cerebellin 1 precursor
ENSG00000103005	USB1	U6 snRNA biogenesis phosphodiesterase 1
ENSG00000103024	NME3	NME/NM23 nucleoside diphosphate kinase 3
ENSG00000103034	NDRG4	NDRG family member 4
ENSG00000103042	SLC38A7	solute carrier family 38 member 7
ENSG00000103056	SMPD3	sphingomyelin phosphodiesterase 3
ENSG00000103066	PLA2G15	phospholipase A2 group XV
ENSG00000103111	MON1B	MON1 homolog B, secretory trafficking associated
ENSG00000103145	HCFC1R1	host cell factor C1 regulator 1
ENSG00000103148	NPRL3	NPR3 like, GATOR1 complex subunit
ENSG00000103184	SEC14L5	SEC14 like lipid binding 5
ENSG00000103202	NME4	NME/NM23 nucleoside diphosphate kinase 4
ENSG00000103249	CLCN7	chloride voltage-gated channel 7
ENSG00000103254	ANTKMT	adenine nucleotide translocase lysine methyltransferase

ENSG00000103260	METRN	meteorin, glial cell differentiation regulator
ENSG00000103274	NUBP1	nucleotide binding protein 1
ENSG00000103326	CAPN15	calpain 15
ENSG00000103495	MAZ	MYC associated zinc finger protein
ENSG00000103496	STX4	syntaxin 4
ENSG00000103502	CDIPT	CDP-diacylglycerol--inositol 3-phosphatidyltransferase
ENSG00000103740	ACSBG1	acyl-CoA synthetase bubblegum family member 1
ENSG00000104059	FAM189A1	family with sequence similarity 189 member A1
ENSG00000104142	VPS18	VPS18 core subunit of CORVET and HOPS complexes
ENSG00000104341	LAPTM4B	lysosomal protein transmembrane 4 beta
ENSG00000104415	CCN4	cellular communication network factor 4
ENSG00000104497	SNX16	sorting nexin 16
ENSG00000104823	ECH1	enoyl-CoA hydratase 1
ENSG00000104824	HNRNPL	heterogeneous nuclear ribonucleoprotein L
ENSG00000104853	CLPTM1	CLPTM1 regulator of GABA type A receptor forward trafficking
ENSG00000104859	CLASRP	CLK4 associating serine/arginine rich protein
ENSG00000104870	FCGRT	Fc fragment of IgG receptor and transporter
ENSG00000104883	PEX11G	peroxisomal biogenesis factor 11 gamma
ENSG00000104886	PLEKHJ1	pleckstrin homology domain containing J1
ENSG00000104946	TBC1D17	TBC1 domain family member 17
ENSG00000104953	TLE6	TLE family member 6, subcortical maternal complex member
ENSG00000104957	CCDC130	coiled-coil domain containing 130
ENSG00000104976	SNAPC2	small nuclear RNA activating complex polypeptide 2
ENSG00000105053	VRK3	VRK serine/threonine kinase 3
ENSG00000105058	FAM32A	family with sequence similarity 32 member A
ENSG00000105063	PPP6R1	protein phosphatase 6 regulatory subunit 1
ENSG00000105135	ILVBL	ilvB acetolactate synthase like
ENSG00000105137	SYDE1	synapse defective Rho GTPase homolog 1
ENSG00000105143	SLC1A6	solute carrier family 1 member 6
ENSG00000105197	TIMM50	translocase of inner mitochondrial membrane 50
ENSG00000105204	DYRK1B	dual specificity tyrosine phosphorylation regulated kinase 1B
ENSG00000105223	PLD3	phospholipase D family member 3
ENSG00000105227	PRX	periaxin
ENSG00000105251	SHD	Src homology 2 domain containing transforming protein D
ENSG00000105261	OVOL3	ovo like zinc finger 3
ENSG00000105281	SLC1A5	solute carrier family 1 member 5
ENSG00000105298	CACTIN	cactin, spliceosome C complex subunit
ENSG00000105341	DMAC2	distal membrane arm assembly complex 2
ENSG00000105357	MYH14	myosin heavy chain 14
ENSG00000105364	MRPL4	mitochondrial ribosomal protein L4
ENSG00000105397	TYK2	tyrosine kinase 2
ENSG00000105401	CDC37	cell division cycle 37, HSP90 cochaperone
ENSG00000105402	NAPA	NSF attachment protein alpha

ENSG00000105438	KDELR1	KDEL endoplasmic reticulum protein retention receptor 1
ENSG00000105479	CCDC114	coiled-coil domain containing 114
ENSG00000105556	MIER2	MIER family member 2
ENSG00000105605	CACNG7	calcium voltage-gated channel auxiliary subunit gamma 7
ENSG00000105609	LILRB5	leukocyte immunoglobulin like receptor B5
ENSG00000105613	MAST1	microtubule associated serine/threonine kinase 1
ENSG00000105618	PRPF31	pre-mRNA processing factor 31
ENSG00000105642	KCNN1	potassium calcium-activated channel subfamily N member 1
ENSG00000105643	ARRDC2	arrestin domain containing 2
ENSG00000105662	CRTC1	CREB regulated transcription coactivator 1
ENSG00000105664	COMP	cartilage oligomeric matrix protein
ENSG00000105669	COPE	COPI coat complex subunit epsilon
ENSG00000105677	TMEM147	transmembrane protein 147
ENSG00000105679	GAPDHS	glyceraldehyde-3-phosphate dehydrogenase, spermatogenic
ENSG00000105697	HAMP	hepcidin antimicrobial peptide
ENSG00000105698	USF2	upstream transcription factor 2, c-fos interacting
ENSG00000105700	KXD1	KxDL motif containing 1
ENSG00000105732	ZNF574	zinc finger protein 574
ENSG00000105771	SMG9	SMG9 nonsense mediated mRNA decay factor
ENSG00000105875	WDR91	WD repeat domain 91
ENSG00000105967	TFEC	transcription factor EC
ENSG00000105974	CAV1	caveolin 1
ENSG00000106123	EPHB6	EPH receptor B6
ENSG00000106211	HSPB1	heat shock protein family B (small) member 1
ENSG00000106236	NPTX2	neuronal pentraxin 2
ENSG00000106299	WASL	WASP like actin nucleation promoting factor
ENSG00000106367	AP1S1	adaptor related protein complex 1 subunit sigma 1
ENSG00000106397	PLOD3	procollagen-lysine,2-oxoglutarate 5-dioxygenase 3
ENSG00000106436	MYL10	myosin light chain 10
ENSG00000106609	TMEM248	transmembrane protein 248
ENSG00000106665	CLIP2	CAP-Gly domain containing linker protein 2
ENSG00000106683	LIMK1	LIM domain kinase 1
ENSG00000106991	ENG	endoglin
ENSG00000107140	TESK1	testis associated actin remodelling kinase 1
ENSG00000107164	FUBP3	far upstream element binding protein 3
ENSG00000107262	BAG1	BAG cochaperone 1
ENSG00000107281	NPDC1	neural proliferation, differentiation and control 1
ENSG00000107295	SH3GL2	SH3 domain containing GRB2 like 2, endophilin A1
ENSG00000107404	DVL1	dishevelled segment polarity protein 1
ENSG00000107521	HPS1	HPS1 biogenesis of lysosomal organelles complex 3 subunit 1
ENSG00000107562	CXCL12	C-X-C motif chemokine ligand 12
ENSG00000107719	PALD1	phosphatase domain containing paladin 1
ENSG00000107807	TLX1	T cell leukemia homeobox 1

ENSG00000107816	LZTS2	leucine zipper tumor suppressor 2
ENSG00000107819	SFXN3	sideroflexin 3
ENSG00000107872	FBXL15	F-box and leucine rich repeat protein 15
ENSG00000107984	DKK1	dickkopf WNT signaling pathway inhibitor 1
ENSG00000108107	RPL28	ribosomal protein L28
ENSG00000108255	CRYBA1	crystallin beta A1
ENSG00000108298	RPL19	ribosomal protein L19
ENSG00000108309	RUNDC3A	RUN domain containing 3A
ENSG00000108387	SEPTIN4	septin 4
ENSG00000108518	PFN1	profilin 1
ENSG00000108523	RNF167	ring finger protein 167
ENSG00000108528	SLC25A11	solute carrier family 25 member 11
ENSG00000108557	RAI1	retinoic acid induced 1
ENSG00000108604	SMARCD2	SWI/SNF related, matrix associated, actin dependent regulator of chromatin, subfamily d, member 2
ENSG00000108622	ICAM2	intercellular adhesion molecule 2
ENSG00000108839	ALOX12	arachidonate 12-lipoxygenase, 12S type
ENSG00000108883	EFTUD2	elongation factor Tu GTP binding domain containing 2
ENSG00000108960	MMD	monocyte to macrophage differentiation associated
ENSG00000109016	DHRS7B	dehydrogenase/reductase 7B
ENSG00000109066	TMEM104	transmembrane protein 104
ENSG00000109180	OCIAD1	OCIA domain containing 1
ENSG00000109501	WFS1	wolframin ER transmembrane glycoprotein
ENSG00000109971	HSPA8	heat shock protein family A (Hsp70) member 8
ENSG00000110011	DNAJC4	DnaJ heat shock protein family (Hsp40) member C4
ENSG00000110057	UNC93B1	unc-93 homolog B1, TLR signaling regulator
ENSG00000110108	TMEM109	transmembrane protein 109
ENSG00000110171	TRIM3	tripartite motif containing 3
ENSG00000110237	ARHGEF17	Rho guanine nucleotide exchange factor 17
ENSG00000110244	APOA4	apolipoprotein A4
ENSG00000110492	MDK	midkine
ENSG00000110514	MADD	MAP kinase activating death domain
ENSG00000110693	SOX6	SRY-box transcription factor 6
ENSG00000110697	PITPNM1	phosphatidylinositol transfer protein membrane associated 1
ENSG00000110700	RPS13	ribosomal protein S13
ENSG00000110711	AIP	aryl hydrocarbon receptor interacting protein
ENSG00000110717	NDUFS8	NADH:ubiquinone oxidoreductase core subunit S8
ENSG00000110723	EXPH5	exophilin 5
ENSG00000110906	KCTD10	potassium channel tetramerization domain containing 10
ENSG00000110925	CSRNP2	cysteine and serine rich nuclear protein 2
ENSG00000110955	ATP5F1B	ATP synthase F1 subunit beta
ENSG00000110958	PTGES3	prostaglandin E synthase 3
ENSG00000111321	LTBR	lymphotoxin beta receptor

ENSG00000111328	CDK2AP1	cyclin dependent kinase 2 associated protein 1
ENSG00000111344	RASAL1	RAS protein activator like 1
ENSG00000111540	RAB5B	RAB5B, member RAS oncogene family
ENSG00000111671	SPSB2	splA/ryanodine receptor domain and SOCS box containing 2
ENSG00000111676	ATN1	atrophin 1
ENSG00000111679	PTPN6	protein tyrosine phosphatase non-receptor type 6
ENSG00000111843	TMEM14C	transmembrane protein 14C
ENSG00000112335	SNX3	sorting nexin 3
ENSG00000112514	CUTA	cutA divalent cation tolerance homolog
ENSG00000112561	TFEB	transcription factor EB
ENSG00000112787	FBRSL1	fibrosin like 1
ENSG00000113108	APBB3	amyloid beta precursor protein binding family B member 3
ENSG00000113578	FGF1	fibroblast growth factor 1
ENSG00000113648	MACROH2A1	macroH2A.1 histone
ENSG00000113732	ATP6V0E1	ATPase H+ transporting V0 subunit e1
ENSG00000113763	UNC5A	unc-5 netrin receptor A
ENSG00000113811	SELENOK	selenoprotein K
ENSG00000113845	TIMMDC1	translocase of inner mitochondrial membrane domain containing 1
ENSG00000114349	GNAT1	G protein subunit alpha transducin 1
ENSG00000114354	TFG	trafficking from ER to golgi regulator
ENSG00000114631	PODXL2	podocalyxin like 2
ENSG00000114853	ZBTB47	zinc finger and BTB domain containing 47
ENSG00000114859	CLCN2	chloride voltage-gated channel 2
ENSG00000114902	SPCS1	signal peptidase complex subunit 1
ENSG00000114956	DGUOK	deoxyguanosine kinase
ENSG00000115041	KCNIP3	potassium voltage-gated channel interacting protein 3
ENSG00000115129	TP53I3	tumor protein p53 inducible protein 3
ENSG00000115170	ACVR1	activin A receptor type 1
ENSG00000115194	SLC30A3	solute carrier family 30 member 3
ENSG00000115204	MPV17	mitochondrial inner membrane protein MPV17
ENSG00000115211	EIF2B4	eukaryotic translation initiation factor 2B subunit delta
ENSG00000115241	PPM1G	protein phosphatase, Mg2+/Mn2+ dependent 1G
ENSG00000115268	RPS15	ribosomal protein S15
ENSG00000115275	MOGS	mannosyl-oligosaccharide glucosidase
ENSG00000115289	PCGF1	polycomb group ring finger 1
ENSG00000115307	AUP1	AUP1 lipid droplet regulating VLDL assembly factor
ENSG00000115318	LOXL3	lysyl oxidase like 3
ENSG00000115468	EFHD1	EF-hand domain family member D1
ENSG00000115525	ST3GAL5	ST3 beta-galactoside alpha-2,3-sialyltransferase 5
ENSG00000115590	IL1R2	interleukin 1 receptor type 2
ENSG00000115694	STK25	serine/threonine kinase 25
ENSG00000115806	GORASP2	golgi reassembly stacking protein 2
ENSG00000115884	SDC1	syndecan 1

ENSG00000116014	KISS1R	KISS1 receptor
ENSG00000116016	EPAS1	endothelial PAS domain protein 1
ENSG00000116032	GRIN3B	glutamate ionotropic receptor NMDA type subunit 3B
ENSG00000116035	VAX2	ventral anterior homeobox 2
ENSG00000116044	NFE2L2	nuclear factor, erythroid 2 like 2
ENSG00000116133	DHCR24	24-dehydrocholesterol reductase
ENSG00000116209	TMEM59	transmembrane protein 59
ENSG00000116254	CHD5	chromodomain helicase DNA binding protein 5
ENSG00000116299	ELAPOR1	endosome-lysosome associated apoptosis and autophagy regulator 1
ENSG00000116521	SCAMP3	secretory carrier membrane protein 3
ENSG00000116586	LAMTOR2	late endosomal/lysosomal adaptor, MAPK and MTOR activator 2
ENSG00000116604	MEF2D	myocyte enhancer factor 2D
ENSG00000116649	SRM	spermidine synthase
ENSG00000116661	FBXO2	F-box protein 2
ENSG00000116670	MAD2L2	mitotic arrest deficient 2 like 2
ENSG00000116685	KIAA2013	KIAA2013
ENSG00000116774	OLFML3	olfactomedin like 3
ENSG00000116819	TFAP2E	transcription factor AP-2 epsilon
ENSG00000116871	MAP7D1	MAP7 domain containing 1
ENSG00000116885	OSCP1	organic solute carrier partner 1
ENSG00000117115	PADI2	peptidyl arginine deiminase 2
ENSG00000117308	GALE	UDP-galactose-4-epimerase
ENSG00000117318	ID3	inhibitor of DNA binding 3, HLH protein
ENSG00000117362	APH1A	aph-1 homolog A, gamma-secretase subunit
ENSG00000117410	ATP6V0B	ATPase H+ transporting V0 subunit b
ENSG00000117448	AKR1A1	aldo-keto reductase family 1 member A1
ENSG00000117592	PRDX6	peroxiredoxin 6
ENSG00000117676	RPS6KA1	ribosomal protein S6 kinase A1
ENSG00000117691	NENF	neudesin neurotrophic factor
ENSG00000117862	TXNDC12	thioredoxin domain containing 12
ENSG00000117877	POLR1G	RNA polymerase I subunit G
ENSG00000117971	CHRNB4	cholinergic receptor nicotinic beta 4 subunit
ENSG00000118137	APOA1	apolipoprotein A1
ENSG00000118181	RPS25	ribosomal protein S25
ENSG00000118200	CAMSAP2	calmodulin regulated spectrin associated protein family member 2
ENSG00000118276	B4GALT6	beta-1,4-galactosyltransferase 6
ENSG00000118526	TCF21	transcription factor 21
ENSG00000118557	PMFBP1	polyamine modulated factor 1 binding protein 1
ENSG00000118579	MED28	mediator complex subunit 28
ENSG00000118640	VAMP8	vesicle associated membrane protein 8
ENSG00000118707	TGIF2	TGFB induced factor homeobox 2
ENSG00000118816	CCNI	cyclin I
ENSG00000118855	MFSD1	major facilitator superfamily domain containing 1

ENSG00000118972	FGF23	fibroblast growth factor 23
ENSG00000118990	GLRXP3	glutaredoxin pseudogene 3
ENSG00000119013	NDUFB3	NADH:ubiquinone oxidoreductase subunit B3
ENSG00000119227	PIGZ	phosphatidylinositol glycan anchor biosynthesis class Z
ENSG00000119283	TRIM67	tripartite motif containing 67
ENSG00000119396	RAB14	RAB14, member RAS oncogene family
ENSG00000119414	PPP6C	protein phosphatase 6 catalytic subunit
ENSG00000119547	ONECUT2	one cut homeobox 2
ENSG00000119559	C19orf25	chromosome 19 open reading frame 25
ENSG00000119574	ZBTB45	zinc finger and BTB domain containing 45
ENSG00000119630	PGF	placental growth factor
ENSG00000119655	NPC2	NPC intracellular cholesterol transporter 2
ENSG00000119669	IRF2BPL	interferon regulatory factor 2 binding protein like
ENSG00000119673	ACOT2	acyl-CoA thioesterase 2
ENSG00000119760	SUPT7L	SPT7 like, STAGA complex subunit gamma
ENSG00000119865	CNRIP1	cannabinoid receptor interacting protein 1
ENSG00000119899	SLC17A5	solute carrier family 17 member 5
ENSG00000119953	SMNDC1	survival motor neuron domain containing 1
ENSG00000119977	TCTN3	tectonic family member 3
ENSG00000119986	AVPI1	arginine vasopressin induced 1
ENSG00000120057	SFRP5	secreted frizzled related protein 5
ENSG00000120314	WDR55	WD repeat domain 55
ENSG00000120337	TNFSF18	TNF superfamily member 18
ENSG00000120594	PLXDC2	plexin domain containing 2
ENSG00000120738	EGR1	early growth response 1
ENSG00000120913	PDLIM2	PDZ and LIM domain 2
ENSG00000120942	UBIAD1	UbiA prenyltransferase domain containing 1
ENSG00000121073	SLC35B1	solute carrier family 35 member B1
ENSG00000121454	LHX4	LIM homeobox 4
ENSG00000121743	GJA3	gap junction protein alpha 3
ENSG00000121774	KHDRBS1	KH RNA binding domain containing, signal transduction associated 1
ENSG00000122133	PAEP	progestagen associated endometrial protein
ENSG00000122140	MRPS2	mitochondrial ribosomal protein S2
ENSG00000122254	HS3ST2	heparan sulfate-glucosamine 3-sulfotransferase 2
ENSG00000122367	LDB3	LIM domain binding 3
ENSG00000122386	ZNF205	zinc finger protein 205
ENSG00000122490	SLC66A2	solute carrier family 66 member 2
ENSG00000122515	ZMIZ2	zinc finger MIZ-type containing 2
ENSG00000122592	HOXA7	homeobox A7
ENSG00000122691	TWIST1	twist family bHLH transcription factor 1
ENSG00000122705	CLTA	clathrin light chain A
ENSG00000122756	CNTFR	ciliary neurotrophic factor receptor

ENSG00000122877	EGR2	early growth response 2
ENSG00000122971	ACADS	acyl-CoA dehydrogenase short chain
ENSG00000123009	NME2P1	NME2 pseudogene 1
ENSG00000123154	WDR83	WD repeat domain 83
ENSG00000123353	ORMDL2	ORMDL sphingolipid biosynthesis regulator 2
ENSG00000123454	DBH	dopamine beta-hydroxylase
ENSG00000124107	SLPI	secretory leukocyte peptidase inhibitor
ENSG00000124116	WFDC3	WAP four-disulfide core domain 3
ENSG00000124155	PIGT	phosphatidylinositol glycan anchor biosynthesis class T
ENSG00000124164	VAPB	VAMP associated protein B and C
ENSG00000124222	STX16	syntaxin 16
ENSG00000124249	KCNK15	potassium two pore domain channel subfamily K member 15
ENSG00000124251	TP53TG5	TP53 target 5
ENSG00000124333	VAMP7	vesicle associated membrane protein 7
ENSG00000124357	NAGK	N-acetylglucosamine kinase
ENSG00000124380	SNRNP27	small nuclear ribonucleoprotein U4/U6.U5 subunit 27
ENSG00000124493	GRM4	glutamate metabotropic receptor 4
ENSG00000124664	SPDEF	SAM pointed domain containing ETS transcription factor
ENSG00000124701	APOBEC2	apolipoprotein B mRNA editing enzyme catalytic subunit 2
ENSG00000124702	KLHDC3	kelch domain containing 3
ENSG00000124733	MEA1	male-enhanced antigen 1
ENSG00000124772	CPNE5	copine 5
ENSG00000124782	RREB1	ras responsive element binding protein 1
ENSG00000124827	GCM2	glial cells missing transcription factor 2
ENSG00000125046	SSUH2	ssu-2 homolog
ENSG00000125304	TM9SF2	transmembrane 9 superfamily member 2
ENSG00000125378	BMP4	bone morphogenetic protein 4
ENSG00000125384	PTGER2	prostaglandin E receptor 2
ENSG00000125398	SOX9	SRY-box transcription factor 9
ENSG00000125445	MRPS7	mitochondrial ribosomal protein S7
ENSG00000125447	GGA3	golgi associated, gamma adaptin ear containing, ARF binding protein 3
ENSG00000125449	ARMC7	armadillo repeat containing 7
ENSG00000125503	PPP1R12C	protein phosphatase 1 regulatory subunit 12C
ENSG00000125505	MBOAT7	membrane bound O-acyltransferase domain containing 7
ENSG00000125508	SRMS	src-related kinase lacking C-terminal regulatory tyrosine and N-terminal myristylation sites
ENSG00000125510	OPRL1	opioid related nociceptin receptor 1
ENSG00000125533	BHLHE23	basic helix-loop-helix family member e23
ENSG00000125631	HTR5BP	5-hydroxytryptamine receptor 5B, pseudogene
ENSG00000125650	PSPN	persephin
ENSG00000125734	GPR108	G protein-coupled receptor 108
ENSG00000125744	RTN2	reticulon 2
ENSG00000125775	SDCBP2	syndecan binding protein 2

ENSG00000125813	PAX1	paired box 1
ENSG00000125820	NKX2-2	NK2 homeobox 2
ENSG00000125835	SNRPB	small nuclear ribonucleoprotein polypeptides B and B1
ENSG00000125845	BMP2	bone morphogenetic protein 2
ENSG00000125850	OVOL2	ovo like zinc finger 2
ENSG00000125861	GFRA4	GDNF family receptor alpha 4
ENSG00000125877	ITPA	inosine triphosphatase
ENSG00000125878	TCF15	transcription factor 15
ENSG00000125898	FAM110A	family with sequence similarity 110 member A
ENSG00000125901	MRPS26	mitochondrial ribosomal protein S26
ENSG00000125912	NCLN	nicalin
ENSG00000125995	ROMO1	reactive oxygen species modulator 1
ENSG00000126062	TMEM115	transmembrane protein 115
ENSG00000126106	TMEM53	transmembrane protein 53
ENSG00000126215	XRCC3	X-ray repair cross complementing 3
ENSG00000126218	F10	coagulation factor X
ENSG00000126233	SLURP1	secreted LY6/PLAUR domain containing 1
ENSG00000126243	LRFN3	leucine rich repeat and fibronectin type III domain containing 3
ENSG00000126247	CAPNS1	calpain small subunit 1
ENSG00000126249	PDCD2L	programmed cell death 2 like
ENSG00000126254	RBM42	RNA binding motif protein 42
ENSG00000126267	COX6B1	cytochrome c oxidase subunit 6B1
ENSG00000126351	THRA	thyroid hormone receptor alpha
ENSG00000126353	CCR7	C-C motif chemokine receptor 7
ENSG00000126368	NR1D1	nuclear receptor subfamily 1 group D member 1
ENSG00000126457	PRMT1	protein arginine methyltransferase 1
ENSG00000126461	SCAF1	SR-related CTD associated factor 1
ENSG00000126500	FLRT1	fibronectin leucine rich transmembrane protein 1
ENSG00000126562	WNK4	WNK lysine deficient protein kinase 4
ENSG00000126698	DNAJC8	DnaJ heat shock protein family (Hsp40) member C8
ENSG00000126705	AHDC1	AT-hook DNA binding motif containing 1
ENSG00000126746	ZNF384	zinc finger protein 384
ENSG00000126756	UXT	ubiquitously expressed prefoldin like chaperone
ENSG00000126767	ELK1	ETS transcription factor ELK1
ENSG00000126778	SIX1	SIX homeobox 1
ENSG00000126903	SLC10A3	solute carrier family 10 member 3
ENSG00000126934	MAP2K2	mitogen-activated protein kinase kinase 2
ENSG00000127129	EDN2	endothelin 2
ENSG00000127399	LRRC61	leucine rich repeat containing 61
ENSG00000127415	IDUA	alpha-L-iduronidase
ENSG00000127418	FGFRL1	fibroblast growth factor receptor like 1
ENSG00000127445	PIN1	peptidylprolyl cis/trans isomerase, NIMA-interacting 1
ENSG00000127452	FBXL12	F-box and leucine rich repeat protein 12

ENSG00000127554	GFER	growth factor, augmenter of liver regeneration
ENSG00000127564	PKMYT1	protein kinase, membrane associated tyrosine/threonine 1
ENSG00000127578	WFIKKN1	WAP, follistatin/kazal, immunoglobulin, kunitz and netrin domain containing 1
ENSG00000127831	VIL1	villin 1
ENSG00000127948	POR	cytochrome p450 oxidoreductase
ENSG00000128016	ZFP36	ZFP36 ring finger protein
ENSG00000128039	SRD5A3	steroid 5 alpha-reductase 3
ENSG00000128266	GNAZ	G protein subunit alpha z
ENSG00000128268	MGAT3	beta-1,4-mannosyl-glycoprotein 4-beta-N-acetylglucosaminyltransferase
ENSG00000128322	IGLL1	immunoglobulin lambda like polypeptide 1
ENSG00000128463	EMC4	ER membrane protein complex subunit 4
ENSG00000128487	SPECC1	sperm antigen with calponin homology and coiled-coil domains 1
ENSG00000128524	ATP6V1F	ATPase H+ transporting V1 subunit F
ENSG00000128610	FEZF1	FEZ family zinc finger 1
ENSG00000128683	GAD1	glutamate decarboxylase 1
ENSG00000128789	PSMG2	proteasome assembly chaperone 2
ENSG00000129028	THAP10	THAP domain containing 10
ENSG00000129245	FXR2	FMR1 autosomal homolog 2
ENSG00000129472	RAB2B	RAB2B, member RAS oncogene family
ENSG00000129474	AJUBA	ajuba LIM protein
ENSG00000129535	NRL	neural retina leucine zipper
ENSG00000129562	DAD1	defender against cell death 1
ENSG00000129625	REEP5	receptor accessory protein 5
ENSG00000129654	FOXJ1	forkhead box J1
ENSG00000129757	CDKN1C	cyclin dependent kinase inhibitor 1C
ENSG00000129911	KLF16	Kruppel like factor 16
ENSG00000129946	SHC2	SHC adaptor protein 2
ENSG00000129990	SYT5	synaptotagmin 5
ENSG00000130054	FAM155B	family with sequence similarity 155 member B
ENSG00000130173	ANGPTL8	angiopoietin like 8
ENSG00000130208	APOC1	apolipoprotein C1
ENSG00000130244	FAM98C	family with sequence similarity 98 member C
ENSG00000130287	NCAN	neurocan
ENSG00000130303	BST2	bone marrow stromal cell antigen 2
ENSG00000130304	SLC27A1	solute carrier family 27 member 1
ENSG00000130311	DDA1	DET1 and DDB1 associated 1
ENSG00000130312	MRPL34	mitochondrial ribosomal protein L34
ENSG00000130382	MLLT1	MLLT1 super elongation complex subunit
ENSG00000130487	KLHDC7B	kelch domain containing 7B
ENSG00000130517	PGPEP1	pyroglutamyl-peptidase I
ENSG00000130590	SAMD10	sterile alpha motif domain containing 10
ENSG00000130600	H19	H19 imprinted maternally expressed transcript

ENSG00000130643	CALY	calcyon neuron specific vesicular protein
ENSG00000130669	PAK4	p21 (RAC1) activated kinase 4
ENSG00000130675	MNX1	motor neuron and pancreas homeobox 1
ENSG00000130700	GATA5	GATA binding protein 5
ENSG00000130748	TMEM160	transmembrane protein 160
ENSG00000130749	ZC3H4	zinc finger CCCH-type containing 4
ENSG00000130751	NPAS1	neuronal PAS domain protein 1
ENSG00000130755	GMFG	glia maturation factor gamma
ENSG00000130758	MAP3K10	mitogen-activated protein kinase kinase kinase 10
ENSG00000130821	SLC6A8	solute carrier family 6 member 8
ENSG00000130827	PLXNA3	plexin A3
ENSG00000130830	MPP1	membrane palmitoylated protein 1
ENSG00000130881	LRP3	LDL receptor related protein 3
ENSG00000130958	SLC35D2	solute carrier family 35 member D2
ENSG00000131013	PPIL4	peptidylprolyl isomerase like 4
ENSG00000131037	EPS8L1	EPS8 like 1
ENSG00000131095	GFAP	glial fibrillary acidic protein
ENSG00000131171	SH3BGRL	SH3 domain binding glutamate rich protein like
ENSG00000131187	F12	coagulation factor XII
ENSG00000131264	CDX4	caudal type homeobox 4
ENSG00000131408	NR1H2	nuclear receptor subfamily 1 group H member 2
ENSG00000131409	LRRC4B	leucine rich repeat containing 4B
ENSG00000131462	TUBG1	tubulin gamma 1
ENSG00000131475	VPS25	vacuolar protein sorting 25 homolog
ENSG00000131495	NDUFA2	NADH:ubiquinone oxidoreductase subunit A2
ENSG00000131591	C1orf159	chromosome 1 open reading frame 159
ENSG00000131650	KREMEN2	kringle containing transmembrane protein 2
ENSG00000131724	IL13RA1	interleukin 13 receptor subunit alpha 1
ENSG00000131771	PPP1R1B	protein phosphatase 1 regulatory inhibitor subunit 1B
ENSG00000131788	PIAS3	protein inhibitor of activated STAT 3
ENSG00000132000	PODNL1	podocan like 1
ENSG00000132017	DCAF15	DDB1 and CUL4 associated factor 15
ENSG00000132024	CC2D1A	coiled-coil and C2 domain containing 1A
ENSG00000132128	LRRC41	leucine rich repeat containing 41
ENSG00000132164	SLC6A11	solute carrier family 6 member 11
ENSG00000132388	UBE2G1	ubiquitin conjugating enzyme E2 G1
ENSG00000132446	FTHL17	ferritin heavy chain like 17
ENSG00000132471	WBP2	WW domain binding protein 2
ENSG00000132478	UNK	unk zinc finger
ENSG00000132535	DLG4	discs large MAGUK scaffold protein 4
ENSG00000132581	SDF2	stromal cell derived factor 2
ENSG00000132612	VPS4A	vacuolar protein sorting 4 homolog A
ENSG00000132613	MTSS2	MTSS I-BAR domain containing 2

ENSG00000132680	KHDC4	KH domain containing 4, pre-mRNA splicing factor
ENSG00000132768	DPH2	diphthamide biosynthesis 2
ENSG00000132801	ZSWIM3	zinc finger SWIM-type containing 3
ENSG00000132965	ALOX5AP	arachidonate 5-lipoxygenase activating protein
ENSG00000133216	EPHB2	EPH receptor B2
ENSG00000133265	HSPBP1	HSPA (Hsp70) binding protein 1
ENSG00000133321	PLAAT4	phospholipase A and acyltransferase 4
ENSG00000133519	ZDHHC8P1	ZDHHC8 pseudogene 1
ENSG00000133612	AGAP3	ArfGAP with GTPase domain, ankyrin repeat and PH domain 3
ENSG00000133619	KRBA1	KRAB-A domain containing 1
ENSG00000133773	CCDC59	coiled-coil domain containing 59
ENSG00000133874	RNF122	ring finger protein 122
ENSG00000133884	DPF2	double PHD fingers 2
ENSG00000133935	ERG28	ergosterol biosynthesis 28 homolog
ENSG00000134061	CD180	CD180 molecule
ENSG00000134201	GSTM5	glutathione S-transferase mu 5
ENSG00000134256	CD101	CD101 molecule
ENSG00000134278	SPIRE1	spire type actin nucleation factor 1
ENSG00000134287	ARF3	ADP ribosylation factor 3
ENSG00000134443	GRP	gastrin releasing peptide
ENSG00000134569	LRP4	LDL receptor related protein 4
ENSG00000134590	RTL8C	retrotransposon Gag like 8C
ENSG00000134815	DHX34	DExH-box helicase 34
ENSG00000134825	TMEM258	transmembrane protein 258
ENSG00000134917	ADAMTS8	ADAM metallopeptidase with thrombospondin type 1 motif 8
ENSG00000134955	SLC37A2	solute carrier family 37 member 2
ENSG00000135046	ANXA1	annexin A1
ENSG00000135077	HAVCR2	hepatitis A virus cellular receptor 2
ENSG00000135218	CD36	CD36 molecule
ENSG00000135390	ATP5MC2	ATP synthase membrane subunit c locus 2
ENSG00000135506	OS9	OS9 endoplasmic reticulum lectin
ENSG00000135519	KCNH3	potassium voltage-gated channel subfamily H member 3
ENSG00000135821	GLUL	glutamate-ammonia ligase
ENSG00000135824	RGS8	regulator of G protein signaling 8
ENSG00000135838	NPL	N-acetylneuraminate pyruvate lyase
ENSG00000135932	CAB39	calcium binding protein 39
ENSG00000135940	COX5B	cytochrome c oxidase subunit 5B
ENSG00000136002	ARHGEF4	Rho guanine nucleotide exchange factor 4
ENSG00000136021	SCYL2	SCY1 like pseudokinase 2
ENSG00000136114	THSD1	thrombospondin type 1 domain containing 1
ENSG00000136160	EDNRB	endothelin receptor type B
ENSG00000136206	SPDYE1	speedy/RINGO cell cycle regulator family member E1
ENSG00000136235	GPNMB	glycoprotein nmb

ENSG00000136274	NACAD	NAC alpha domain containing
ENSG00000136295	TTYH3	tweety family member 3
ENSG00000136367	ZFHX2	zinc finger homeobox 2
ENSG00000136378	ADAMTS7	ADAM metallopeptidase with thrombospondin type 1 motif 7
ENSG00000136383	ALPK3	alpha kinase 3
ENSG00000136457	CHAD	chondroadherin
ENSG00000136478	TEX2	testis expressed 2
ENSG00000136485	DCAF7	DDB1 and CUL4 associated factor 7
ENSG00000136710	CCDC115	coiled-coil domain containing 115
ENSG00000136826	KLF4	Kruppel like factor 4
ENSG00000136866	ZFP37	ZFP37 zinc finger protein
ENSG00000136867	SLC31A2	solute carrier family 31 member 2
ENSG00000136868	SLC31A1	solute carrier family 31 member 1
ENSG00000136908	DPM2	dolichyl-phosphate mannosyltransferase subunit 2, regulatory
ENSG00000136931	NR5A1	nuclear receptor subfamily 5 group A member 1
ENSG00000136944	LMX1B	LIM homeobox transcription factor 1 beta
ENSG00000136986	DERL1	derlin 1
ENSG00000137073	UBAP2	ubiquitin associated protein 2
ENSG00000137094	DNAJB5	DnaJ heat shock protein family (Hsp40) member B5
ENSG00000137106	GRHPR	glyoxylate and hydroxypyruvate reductase
ENSG00000137142	IGFBPL1	insulin like growth factor binding protein like 1
ENSG00000137161	CNPY3	canopy FGF signaling regulator 3
ENSG00000137166	FOXP4	forkhead box P4
ENSG00000137198	GMPR	guanosine monophosphate reductase
ENSG00000137207	YIPF3	Yip1 domain family member 3
ENSG00000137216	TMEM63B	transmembrane protein 63B
ENSG00000137221	TJAP1	tight junction associated protein 1
ENSG00000137364	TPMT	thiopurine S-methyltransferase
ENSG00000137474	MYO7A	myosin VIIA
ENSG00000137496	IL18BP	interleukin 18 binding protein
ENSG00000137509	PRCP	prolylcarboxypeptidase
ENSG00000137575	SDCBP	syndecan binding protein
ENSG00000137699	TRIM29	tripartite motif containing 29
ENSG00000137727	ARHGAP20	Rho GTPase activating protein 20
ENSG00000137809	ITGA11	integrin subunit alpha 11
ENSG00000137843	PAK6	p21 (RAC1) activated kinase 6
ENSG00000137845	ADAM10	ADAM metallopeptidase domain 10
ENSG00000137877	SPTBN5	spectrin beta, non-erythrocytic 5
ENSG00000137970	RPL7P9	ribosomal protein L7 pseudogene 9
ENSG00000138172	CALHM2	calcium homeostasis modulator family member 2
ENSG00000138434	ITPRID2	ITPR interacting domain containing 2
ENSG00000138622	HCN4	hyperpolarization activated cyclic nucleotide gated potassium channel 4

ENSG00000138760	SCARB2	scavenger receptor class B member 2
ENSG00000138944	SHISAL1	shisa like 1
ENSG00000139044	B4GALNT3	beta-1,4-N-acetyl-galactosaminyltransferase 3
ENSG00000139112	GABARAPL1	GABA type A receptor associated protein like 1
ENSG00000139190	VAMP1	vesicle associated membrane protein 1
ENSG00000139194	RBP5	retinol binding protein 5
ENSG00000139289	PHLDA1	pleckstrin homology like domain family A member 1
ENSG00000139438	FAM222A	family with sequence similarity 222 member A
ENSG00000139445	FOXN4	forkhead box N4
ENSG00000139579	NABP2	nucleic acid binding protein 2
ENSG00000139624	CERS5	ceramide synthase 5
ENSG00000139625	MAP3K12	mitogen-activated protein kinase kinase kinase 12
ENSG00000139636	LMBR1L	limb development membrane protein 1 like
ENSG00000139644	TMBIM6	transmembrane BAX inhibitor motif containing 6
ENSG00000139648	KRT71	keratin 71
ENSG00000139687	RB1	RB transcriptional corepressor 1
ENSG00000139767	SRRM4	serine/arginine repetitive matrix 4
ENSG00000139800	ZIC5	Zic family member 5
ENSG00000139880	CDH24	cadherin 24
ENSG00000139974	SLC38A6	solute carrier family 38 member 6
ENSG00000140264	SERF2	small EDRK-rich factor 2
ENSG00000140299	BNIP2	BCL2 interacting protein 2
ENSG00000140319	SRP14	signal recognition particle 14
ENSG00000140320	BAHD1	bromo adjacent homology domain containing 1
ENSG00000140391	TSPAN3	tetraspanin 3
ENSG00000140497	SCAMP2	secretory carrier membrane protein 2
ENSG00000140632	GLYR1	glyoxylate reductase 1 homolog
ENSG00000140740	UQCRC2	ubiquinol-cytochrome c reductase core protein 2
ENSG00000140795	MYLK3	myosin light chain kinase 3
ENSG00000140931	CMTM3	CKLF like MARVEL transmembrane domain containing 3
ENSG00000140945	CDH13	cadherin 13
ENSG00000140983	RHOT2	ras homolog family member T2
ENSG00000140988	RPS2	ribosomal protein S2
ENSG00000140990	NDUFB10	NADH:ubiquinone oxidoreductase subunit B10
ENSG00000140992	PDPK1	3-phosphoinositide dependent protein kinase 1
ENSG00000141012	GALNS	galactosamine (N-acetyl)-6-sulfatase
ENSG00000141026	MED9	mediator complex subunit 9
ENSG00000141084	RANBP10	RAN binding protein 10
ENSG00000141349	G6PC3	glucose-6-phosphatase catalytic subunit 3
ENSG00000141480	ARRB2	arrestin beta 2
ENSG00000141497	ZMYND15	zinc finger MYND-type containing 15
ENSG00000141503	MINK1	misshapen like kinase 1
ENSG00000141504	SAT2	spermidine/spermine N1-acetyltransferase family member 2

ENSG00000141540	TTYH2	tweety family member 2
ENSG00000141582	CBX4	chromobox 4
ENSG00000141644	MBD1	methyl-CpG binding domain protein 1
ENSG00000141738	GRB7	growth factor receptor bound protein 7
ENSG00000141854	MISP3	MISP family member 3
ENSG00000141858	SAMD1	sterile alpha motif domain containing 1
ENSG00000141867	BRD4	bromodomain containing 4
ENSG00000141933	TPGS1	tubulin polyglutamylase complex subunit 1
ENSG00000141934	PLPP2	phospholipid phosphatase 2
ENSG00000141971	MVB12A	multivesicular body subunit 12A
ENSG00000142039	CCDC97	coiled-coil domain containing 97
ENSG00000142156	COL6A1	collagen type VI alpha 1 chain
ENSG00000142173	COL6A2	collagen type VI alpha 2 chain
ENSG00000142182	DNMT3L	DNA methyltransferase 3 like
ENSG00000142186	SCYL1	SCY1 like pseudokinase 1
ENSG00000142208	AKT1	AKT serine/threonine kinase 1
ENSG00000142235	LMTK3	lemur tyrosine kinase 3
ENSG00000142252	GEMIN7	gem nuclear organelle associated protein 7
ENSG00000142330	CAPN10	calpain 10
ENSG00000142453	CARM1	coactivator associated arginine methyltransferase 1
ENSG00000142459	EVI5L	ecotropic viral integration site 5 like
ENSG00000142507	PSMB6	proteasome 20S subunit beta 6
ENSG00000142513	ACP4	acid phosphatase 4
ENSG00000142655	PEX14	peroxisomal biogenesis factor 14
ENSG00000142657	PGD	phosphogluconate dehydrogenase
ENSG00000142700	DMRTA2	DMRT like family A2
ENSG00000143067	ZNF697	zinc finger protein 697
ENSG00000143079	CTTNBP2NL	CTTNBP2 N-terminal like
ENSG00000143110	C1orf162	chromosome 1 open reading frame 162
ENSG00000143126	CELSR2	cadherin EGF LAG seven-pass G-type receptor 2
ENSG00000143153	ATP1B1	ATPase Na+/K+ transporting subunit beta 1
ENSG00000143167	GPA33	glycoprotein A33
ENSG00000143178	TBX19	T-box transcription factor 19
ENSG00000143183	TMCO1	transmembrane and coiled-coil domains 1
ENSG00000143294	PRCC	proline rich mitotic checkpoint control factor
ENSG00000143303	RRNAD1	ribosomal RNA adenine dimethylase domain containing 1
ENSG00000143344	RGL1	ral guanine nucleotide dissociation stimulator like 1
ENSG00000143368	SF3B4	splicing factor 3b subunit 4
ENSG00000143373	ZNF687	zinc finger protein 687
ENSG00000143382	ADAMTSL4	ADAMTS like 4
ENSG00000143393	PI4KB	phosphatidylinositol 4-kinase beta
ENSG00000143418	CERS2	ceramide synthase 2
ENSG00000143436	MRPL9	mitochondrial ribosomal protein L9

ENSG00000143520	FLG2	filaggrin family member 2
ENSG00000143537	ADAM15	ADAM metallopeptidase domain 15
ENSG00000143569	UBAP2L	ubiquitin associated protein 2 like
ENSG00000143630	HCN3	hyperpolarization activated cyclic nucleotide gated potassium channel 3
ENSG00000143632	ACTA1	actin alpha 1, skeletal muscle
ENSG00000143753	DEGS1	delta 4-desaturase, sphingolipid 1
ENSG00000143761	ARF1	ADP ribosylation factor 1
ENSG00000143774	GUK1	guanylate kinase 1
ENSG00000143816	WNT9A	Wnt family member 9A
ENSG00000143845	ETNK2	ethanolamine kinase 2
ENSG00000143862	ARL8A	ADP ribosylation factor like GTPase 8A
ENSG00000144227	NXPH2	neurexophilin 2
ENSG00000144488	ESPNL	espin like
ENSG00000144524	COPS7B	COP9 signalosome subunit 7B
ENSG00000144567	RETREG2	reticulophagy regulator family member 2
ENSG00000144596	GRIP2	glutamate receptor interacting protein 2
ENSG00000144746	ARL6IP5	ADP ribosylation factor like GTPase 6 interacting protein 5
ENSG00000144840	RABL3	RAB, member of RAS oncogene family like 3
ENSG00000144857	BOC	BOC cell adhesion associated, oncogene regulated
ENSG00000144909	OSBPL11	oxysterol binding protein like 11
ENSG00000145198	VWA5B2	von Willebrand factor A domain containing 5B2
ENSG00000145217	SLC26A1	solute carrier family 26 member 1
ENSG00000145248	SLC10A4	solute carrier family 10 member 4
ENSG00000145284	SCD5	stearoyl-CoA desaturase 5
ENSG00000145354	CISD2	CDGSH iron sulfur domain 2
ENSG00000145506	NKD2	NKD inhibitor of WNT signaling pathway 2
ENSG00000145685	LHFPL2	LHFPL tetraspan subfamily member 2
ENSG00000146063	TRIM41	tripartite motif containing 41
ENSG00000146067	FAM193B	family with sequence similarity 193 member B
ENSG00000146112	PPP1R18	protein phosphatase 1 regulatory subunit 18
ENSG00000146197	SCUBE3	signal peptide, CUB domain and EGF like domain containing 3
ENSG00000146242	TPBG	trophoblast glycoprotein
ENSG00000146373	RNF217	ring finger protein 217
ENSG00000146729	NIPSNAP2	nipsnap homolog 2
ENSG00000146826	MAP11	microtubule associated protein 11
ENSG00000146828	SLC12A9	solute carrier family 12 member 9
ENSG00000147027	TMEM47	transmembrane protein 47
ENSG00000147119	CHST7	carbohydrate sulfotransferase 7
ENSG00000147123	NDUFB11	NADH:ubiquinone oxidoreductase subunit B11
ENSG00000147127	RAB41	RAB41, member RAS oncogene family
ENSG00000147246	HTR2C	5-hydroxytryptamine receptor 2C
ENSG00000147416	ATP6V1B2	ATPase H+ transporting V1 subunit B2

ENSG00000147533	GOLGA7	golgin A7
ENSG00000147804	SLC39A4	solute carrier family 39 member 4
ENSG00000147883	CDKN2B	cyclin dependent kinase inhibitor 2B
ENSG00000147889	CDKN2A	cyclin dependent kinase inhibitor 2A
ENSG00000147955	SIGMAR1	sigma non-opioid intracellular receptor 1
ENSG00000148153	INIP	INTS3 and NABP interacting protein
ENSG00000148215	OR5C1	olfactory receptor family 5 subfamily C member 1
ENSG00000148291	SURF2	surfeit 2
ENSG00000148297	MED22	mediator complex subunit 22
ENSG00000148303	RPL7A	ribosomal protein L7a
ENSG00000148308	GTF3C5	general transcription factor IIIC subunit 5
ENSG00000148339	SLC25A25	solute carrier family 25 member 25
ENSG00000148341	SH3GLB2	SH3 domain containing GRB2 like, endophilin B2
ENSG00000148411	NACC2	NACC family member 2
ENSG00000148426	PROSER2	proline and serine rich 2
ENSG00000148824	MTG1	mitochondrial ribosome associated GTPase 1
ENSG00000148841	ITPRIP	inositol 1,4,5-trisphosphate receptor interacting protein
ENSG00000149021	SCGB1A1	secretoglobin family 1A member 1
ENSG00000149043	SYT8	synaptotagmin 8
ENSG00000149100	EIF3M	eukaryotic translation initiation factor 3 subunit M
ENSG00000149357	LAMTOR1	late endosomal/lysosomal adaptor, MAPK and MTOR activator 1
ENSG00000149380	P4HA3	prolyl 4-hydroxylase subunit alpha 3
ENSG00000149476	TKFC	triokinase and FMN cyclase
ENSG00000149480	MTA2	metastasis associated 1 family member 2
ENSG00000149485	FADS1	fatty acid desaturase 1
ENSG00000149506	ZP1	zona pellucida glycoprotein 1
ENSG00000149532	CPSF7	cleavage and polyadenylation specific factor 7
ENSG00000149575	SCN2B	sodium voltage-gated channel beta subunit 2
ENSG00000149609	C20orf144	chromosome 20 open reading frame 144
ENSG00000149658	YTHDF1	YTH N6-methyladenosine RNA binding protein 1
ENSG00000149781	FERMT3	fermitin family member 3
ENSG00000149806	FAU	FAU ubiquitin like and ribosomal protein S30 fusion
ENSG00000149809	TM7SF2	transmembrane 7 superfamily member 2
ENSG00000149922	TBX6	T-box transcription factor 6
ENSG00000149932	TMEM219	transmembrane protein 219
ENSG00000150457	LATS2	large tumor suppressor kinase 2
ENSG00000150594	ADRA2A	adrenoceptor alpha 2A
ENSG00000150773	PIH1D2	PIH1 domain containing 2
ENSG00000150967	ABCB9	ATP binding cassette subfamily B member 9
ENSG00000151005	TKTL2	transketolase like 2
ENSG00000151093	OXSM	3-oxoacyl-ACP synthase, mitochondrial
ENSG00000151117	TMEM86A	transmembrane protein 86A
ENSG00000151239	TWF1	twinfilin actin binding protein 1

ENSG00000151640	DPYSL4	dihydropyrimidinase like 4
ENSG00000151846	PABPC3	poly(A) binding protein cytoplasmic 3
ENSG00000151952	TMEM132D	transmembrane protein 132D
ENSG00000152315	KCNK13	potassium two pore domain channel subfamily K member 13
ENSG00000152518	ZFP36L2	ZFP36 ring finger protein like 2
ENSG00000152669	CCNO	cyclin O
ENSG00000153037	SRP19	signal recognition particle 19
ENSG00000153208	MERTK	MER proto-oncogene, tyrosine kinase
ENSG00000153303	FRMD1	FERM domain containing 1
ENSG00000153443	UBALD1	UBA like domain containing 1
ENSG00000153551	CMTM7	CKLF like MARVEL transmembrane domain containing 7
ENSG00000153558	FBXL2	F-box and leucine rich repeat protein 2
ENSG00000153714	LURAP1L	leucine rich adaptor protein 1 like
ENSG00000153789	CIBAR2	CBY1 interacting BAR domain containing 2
ENSG00000153989	NUS1	NUS1 dehydrodolichyl diphosphate synthase subunit
ENSG00000154146	NRGN	neurogranin
ENSG00000154342	WNT3A	Wnt family member 3A
ENSG00000154723	ATP5PF	ATP synthase peripheral stalk subunit F6
ENSG00000154764	WNT7A	Wnt family member 7A
ENSG00000155097	ATP6V1C1	ATPase H+ transporting V1 subunit C1
ENSG00000155254	MARVELD1	MARVEL domain containing 1
ENSG00000155265	GOLGA7B	golgin A7 family member B
ENSG00000155506	LARP1	La ribonucleoprotein 1, translational regulator
ENSG00000155760	FZD7	frizzled class receptor 7
ENSG00000155876	RRAGA	Ras related GTP binding A
ENSG00000155980	KIF5A	kinesin family member 5A
ENSG00000156150	ALX3	ALX homeobox 3
ENSG00000156171	DRAM2	DNA damage regulated autophagy modulator 2
ENSG00000156304	SCAF4	SR-related CTD associated factor 4
ENSG00000156486	KCNS2	potassium voltage-gated channel modifier subfamily S member 2
ENSG00000156853	ZNF689	zinc finger protein 689
ENSG00000156858	PRR14	proline rich 14
ENSG00000156873	PHKG2	phosphorylase kinase catalytic subunit gamma 2
ENSG00000156925	ZIC3	Zic family member 3
ENSG00000156973	PDE6D	phosphodiesterase 6D
ENSG00000157005	SST	somatostatin
ENSG00000157017	GHRL	ghrelin and obestatin prepropeptide
ENSG00000157191	NECAP2	NECAP endocytosis associated 2
ENSG00000157240	FZD1	frizzled class receptor 1
ENSG00000157450	RNF111	ring finger protein 111
ENSG00000157483	MYO1E	myosin IE
ENSG00000157538	VPS26C	VPS26 endosomal protein sorting factor C
ENSG00000157613	CREB3L1	cAMP responsive element binding protein 3 like 1

ENSG00000157782	CABP1	calcium binding protein 1
ENSG00000157827	FMNL2	formin like 2
ENSG00000157965	SSX8P	SSX family member 8, pseudogene
ENSG00000158008	EXTL1	exostosin like glycosyltransferase 1
ENSG00000158062	UBXN11	UBX domain protein 11
ENSG00000158092	NCK1	NCK adaptor protein 1
ENSG00000158106	RHPN1	rhophilin Rho GTPase binding protein 1
ENSG00000158156	XKR8	XK related 8
ENSG00000158186	MRAS	muscle RAS oncogene homolog
ENSG00000158258	CLSTN2	calsyntenin 2
ENSG00000158292	GPR153	G protein-coupled receptor 153
ENSG00000158373	H2BC5	H2B clustered histone 5
ENSG00000158423	RIBC1	RIB43A domain with coiled-coils 1
ENSG00000158445	KCNB1	potassium voltage-gated channel subfamily B member 1
ENSG00000158458	NRG2	neuregulin 2
ENSG00000158545	ZC3H18	zinc finger CCCH-type containing 18
ENSG00000158604	TMED4	transmembrane p24 trafficking protein 4
ENSG00000158639	PAGE5	PAGE family member 5
ENSG00000158747	NBL1	NBL1, DAN family BMP antagonist
ENSG00000158748	HTR6	5-hydroxytryptamine receptor 6
ENSG00000158773	USF1	upstream transcription factor 1
ENSG00000158793	NIT1	nitrilase 1
ENSG00000158815	FGF17	fibroblast growth factor 17
ENSG00000158850	B4GALT3	beta-1,4-galactosyltransferase 3
ENSG00000158863	FAM160B2	family with sequence similarity 160 member B2
ENSG00000158869	FCER1G	Fc fragment of IgE receptor Ig
ENSG00000158874	APOA2	apolipoprotein A2
ENSG00000158955	WNT9B	Wnt family member 9B
ENSG00000159173	TNNI1	troponin I1, slow skeletal type
ENSG00000159214	CCDC24	coiled-coil domain containing 24
ENSG00000159335	PTMS	parathymosin
ENSG00000159337	PLA2G4D	phospholipase A2 group IVD
ENSG00000159423	ALDH4A1	aldehyde dehydrogenase 4 family member A1
ENSG00000159692	CTBP1	C-terminal binding protein 1
ENSG00000159714	ZDHHC1	zinc finger DHHC-type containing 1
ENSG00000159720	ATP6V0D1	ATPase H+ transporting V0 subunit d1
ENSG00000159761	C16orf86	chromosome 16 open reading frame 86
ENSG00000159788	RGS12	regulator of G protein signaling 12
ENSG00000159792	PSKH1	protein serine kinase H1
ENSG00000159873	CCDC117	coiled-coil domain containing 117
ENSG00000160014	CALM3	calmodulin 3
ENSG00000160049	DFFA	DNA fragmentation factor subunit alpha
ENSG00000160072	ATAD3B	ATPase family AAA domain containing 3B

ENSG00000160094	ZNF362	zinc finger protein 362
ENSG00000160194	NDUFV3	NADH:ubiquinone oxidoreductase subunit V3
ENSG00000160214	RRP1	ribosomal RNA processing 1
ENSG00000160298	C21orf58	chromosome 21 open reading frame 58
ENSG00000160360	GPSM1	G protein signaling modulator 1
ENSG00000160396	HIPK4	homeodomain interacting protein kinase 4
ENSG00000160401	CFAP157	cilia and flagella associated protein 157
ENSG00000160404	TOR2A	torsin family 2 member A
ENSG00000160439	RDH13	retinol dehydrogenase 13
ENSG00000160469	BRSK1	BR serine/threonine kinase 1
ENSG00000160539	PLPP7	phospholipid phosphatase 7 (inactive)
ENSG00000160570	DEDD2	death effector domain containing 2
ENSG00000160685	ZBTB7B	zinc finger and BTB domain containing 7B
ENSG00000160688	FLAD1	flavin adenine dinucleotide synthetase 1
ENSG00000160695	VPS11	VPS11 core subunit of CORVET and HOPS complexes
ENSG00000160712	IL6R	interleukin 6 receptor
ENSG00000160714	UBE2Q1	ubiquitin conjugating enzyme E2 Q1
ENSG00000160716	CHRNB2	cholinergic receptor nicotinic beta 2 subunit
ENSG00000160741	CRTC2	CREB regulated transcription coactivator 2
ENSG00000160767	FAM189B	family with sequence similarity 189 member B
ENSG00000160789	LMNA	lamin A/C
ENSG00000160801	PTH1R	parathyroid hormone 1 receptor
ENSG00000160803	UBQLN4	ubiquilin 4
ENSG00000160813	PPP1R35	protein phosphatase 1 regulatory subunit 35
ENSG00000160856	FCRL3	Fc receptor like 3
ENSG00000160877	NACC1	nucleus accumbens associated 1
ENSG00000160886	LY6K	lymphocyte antigen 6 family member K
ENSG00000160959	LRRC14	leucine rich repeat containing 14
ENSG00000160963	COL26A1	collagen type XXVI alpha 1 chain
ENSG00000160972	PPP1R16A	protein phosphatase 1 regulatory subunit 16A
ENSG00000160999	SH2B2	SH2B adaptor protein 2
ENSG00000161011	SQSTM1	sequestosome 1
ENSG00000161013	MGAT4B	alpha-1,3-mannosyl-glycoprotein 4-beta-N-acetylglucosaminyltransferase B
ENSG00000161021	MAML1	mastermind like transcriptional coactivator 1
ENSG00000161082	CELF5	CUGBP Elav-like family member 5
ENSG00000161091	MFSD12	major facilitator superfamily domain containing 12
ENSG00000161202	DVL3	dishevelled segment polarity protein 3
ENSG00000161203	AP2M1	adaptor related protein complex 2 subunit mu 1
ENSG00000161217	PCYT1A	phosphate cytidylyltransferase 1, choline, alpha
ENSG00000161249	DMKN	dermokine
ENSG00000161277	THAP8	THAP domain containing 8
ENSG00000161395	PGAP3	post-GPI attachment to proteins phospholipase 3

ENSG00000161509	GRIN2C	glutamate ionotropic receptor NMDA type subunit 2C
ENSG00000161544	CYGB	cytoglobin
ENSG00000161558	TMEM143	transmembrane protein 143
ENSG00000161647	MPP3	membrane palmitoylated protein 3
ENSG00000161652	IZUMO2	IZUMO family member 2
ENSG00000161653	NAGS	N-acetylglutamate synthase
ENSG00000161671	EMC10	ER membrane protein complex subunit 10
ENSG00000161798	AQP5	aquaporin 5
ENSG00000161847	RAVER1	ribonucleoprotein, PTB binding 1
ENSG00000161905	ALOX15	arachidonate 15-lipoxygenase
ENSG00000161911	TREML1	triggering receptor expressed on myeloid cells like 1
ENSG00000161921	CXCL16	C-X-C motif chemokine ligand 16
ENSG00000162009	SSTR5	somatostatin receptor 5
ENSG00000162267	ITIH3	inter-alpha-trypsin inhibitor heavy chain 3
ENSG00000162298	SYVN1	synoviolin 1
ENSG00000162396	PARS2	prolyl-tRNA synthetase 2, mitochondrial
ENSG00000162430	SELENON	selenoprotein N
ENSG00000162460	TMEM82	transmembrane protein 82
ENSG00000162490	DRAXIN	dorsal inhibitory axon guidance protein
ENSG00000162496	DHRS3	dehydrogenase/reductase 3
ENSG00000162512	SDC3	syndecan 3
ENSG00000162517	PEF1	penta-EF-hand domain containing 1
ENSG00000162545	CAMK2N1	calcium/calmodulin dependent protein kinase II inhibitor 1
ENSG00000162571	TTLL10	tubulin tyrosine ligase like 10
ENSG00000162572	SCNN1D	sodium channel epithelial 1 subunit delta
ENSG00000162650	ATXN7L2	ataxin 7 like 2
ENSG00000162722	TRIM58	tripartite motif containing 58
ENSG00000162736	NCSTN	nicastrin
ENSG00000162755	KLHDC9	kelch domain containing 9
ENSG00000162771	FAM71A	family with sequence similarity 71 member A
ENSG00000162804	SNED1	sushi, nidogen and EGF like domains 1
ENSG00000162849	KIF26B	kinesin family member 26B
ENSG00000162894	FCMR	Fc fragment of IgM receptor
ENSG00000162949	CAPN13	calpain 13
ENSG00000162976	SLC66A3	solute carrier family 66 member 3
ENSG00000163013	FBXO41	F-box protein 41
ENSG00000163081	CCDC140	CCDC140 long non-coding RNA
ENSG00000163131	CTSS	cathepsin S
ENSG00000163159	VPS72	vacuolar protein sorting 72 homolog
ENSG00000163191	S100A11	S100 calcium binding protein A11
ENSG00000163207	IVL	involucrin
ENSG00000163221	S100A12	S100 calcium binding protein A12
ENSG00000163362	INAVA	innate immunity activator

ENSG00000163374	YY1AP1	YY1 associated protein 1
ENSG00000163453	IGFBP7	insulin like growth factor binding protein 7
ENSG00000163466	ARPC2	actin related protein 2/3 complex subunit 2
ENSG00000163472	TMEM79	transmembrane protein 79
ENSG00000163485	ADORA1	adenosine A1 receptor
ENSG00000163497	FEV	FEV transcription factor, ETS family member
ENSG00000163501	IHH	Indian hedgehog signaling molecule
ENSG00000163512	AZI2	5-azacytidine induced 2
ENSG00000163517	HDAC11	histone deacetylase 11
ENSG00000163519	TRAT1	T cell receptor associated transmembrane adaptor 1
ENSG00000163623	NKX6-1	NK6 homeobox 1
ENSG00000163683	SMIM14	small integral membrane protein 14
ENSG00000163754	GYG1	glycogenin 1
ENSG00000163795	ZNF513	zinc finger protein 513
ENSG00000163812	ZDHHC3	zinc finger DHHC-type palmitoyltransferase 3
ENSG00000163882	POLR2H	RNA polymerase II, I and III subunit H
ENSG00000163888	CAMK2N2	calcium/calmodulin dependent protein kinase II inhibitor 2
ENSG00000163930	BAP1	BRCA1 associated protein 1
ENSG00000163956	LRPAP1	LDL receptor related protein associated protein 1
ENSG00000164008	C1orf50	chromosome 1 open reading frame 50
ENSG00000164050	PLXNB1	plexin B1
ENSG00000164077	MON1A	MON1 homolog A, secretory trafficking associated
ENSG00000164078	MST1R	macrophage stimulating 1 receptor
ENSG00000164111	ANXA5	annexin A5
ENSG00000164112	TMEM155	transmembrane protein 155
ENSG00000164125	GASK1B	golgi associated kinase 1B
ENSG00000164172	MOCS2	molybdenum cofactor synthesis 2
ENSG00000164332	UBLCP1	ubiquitin like domain containing CTD phosphatase 1
ENSG00000164442	CITED2	Cbp/p300 interacting transactivator with Glu/Asp rich carboxy-terminal domain 2
ENSG00000164694	FNDC1	fibronectin type III domain containing 1
ENSG00000164733	CTSB	cathepsin B
ENSG00000164794	KCNV1	potassium voltage-gated channel modifier subfamily V member 1
ENSG00000164841	TMEM74	transmembrane protein 74
ENSG00000164853	UNCX	UNC homeobox
ENSG00000164855	TMEM184A	transmembrane protein 184A
ENSG00000164877	MICALL2	MICAL like 2
ENSG00000164885	CDK5	cyclin dependent kinase 5
ENSG00000164889	SLC4A2	solute carrier family 4 member 2
ENSG00000164897	TMUB1	transmembrane and ubiquitin like domain containing 1
ENSG00000164970	FAM219A	family with sequence similarity 219 member A
ENSG00000165171	METTL27	methyltransferase like 27
ENSG00000165175	MID1IP1	MID1 interacting protein 1

ENSG00000165283	STOML2	stomatin like 2
ENSG00000165300	SLITRK5	SLIT and NTRK like family member 5
ENSG00000165424	ZCCHC24	zinc finger CCHC-type containing 24
ENSG00000165475	CRYL1	crystallin lambda 1
ENSG00000165496	RPL10L	ribosomal protein L10 like
ENSG00000165644	COMTD1	catechol-O-methyltransferase domain containing 1
ENSG00000165646	SLC18A2	solute carrier family 18 member A2
ENSG00000165685	TMEM52B	transmembrane protein 52B
ENSG00000165731	RET	ret proto-oncogene
ENSG00000165752	STK32C	serine/threonine kinase 32C
ENSG00000165804	ZNF219	zinc finger protein 219
ENSG00000165807	PPP1R36	protein phosphatase 1 regulatory subunit 36
ENSG00000165861	ZFYVE1	zinc finger FYVE-type containing 1
ENSG00000165887	ANKRD2	ankyrin repeat domain 2
ENSG00000165914	TTC7B	tetratricopeptide repeat domain 7B
ENSG00000165915	SLC39A13	solute carrier family 39 member 13
ENSG00000165948	IFI27L1	interferon alpha inducible protein 27 like 1
ENSG00000165985	C1QL3	complement C1q like 3
ENSG00000166033	HTRA1	HtrA serine peptidase 1
ENSG00000166106	ADAMTS15	ADAM metallopeptidase with thrombospondin type 1 motif 15
ENSG00000166136	NDUFB8	NADH:ubiquinone oxidoreductase subunit B8
ENSG00000166183	ASPG	asparaginase
ENSG00000166189	HPS6	HPS6 biogenesis of lysosomal organelles complex 2 subunit 3
ENSG00000166228	PCBD1	pterin-4 alpha-carbinolamine dehydratase 1
ENSG00000166311	SMPD1	sphingomyelin phosphodiesterase 1
ENSG00000166402	TUB	TUB bipartite transcription factor
ENSG00000166426	CRABP1	cellular retinoic acid binding protein 1
ENSG00000166452	AKIP1	A-kinase interacting protein 1
ENSG00000166492	FAM86GP	family with sequence similarity 86 member G, pseudogene
ENSG00000166546	BEAN1	brain expressed associated with NEDD4 1
ENSG00000166619	BLCAP	BLCAP apoptosis inducing factor
ENSG00000166716	ZNF592	zinc finger protein 592
ENSG00000166793	YPEL4	yippee like 4
ENSG00000166794	PPIB	peptidylprolyl isomerase B
ENSG00000166797	CIAO2A	cytosolic iron-sulfur assembly component 2A
ENSG00000166823	MESP1	mesoderm posterior bHLH transcription factor 1
ENSG00000166886	NAB2	NGFI-A binding protein 2
ENSG00000166888	STAT6	signal transducer and activator of transcription 6
ENSG00000166925	TSC22D4	TSC22 domain family member 4
ENSG00000166987	MBD6	methyl-CpG binding domain protein 6
ENSG00000167074	TEF	TEF transcription factor, PAR bZIP family member
ENSG00000167104	BPIFB6	BPI fold containing family B member 6
ENSG00000167118	URM1	ubiquitin related modifier 1

ENSG00000167157	PRRX2	paired related homeobox 2
ENSG00000167178	ISLR2	immunoglobulin superfamily containing leucine rich repeat 2
ENSG00000167182	SP2	Sp2 transcription factor
ENSG00000167302	TEPSIN	TEPSIN adaptor related protein complex 4 accessory protein
ENSG00000167395	ZNF646	zinc finger protein 646
ENSG00000167459	LINC00905	long intergenic non-protein coding RNA 905
ENSG00000167461	RAB8A	RAB8A, member RAS oncogene family
ENSG00000167468	GPX4	glutathione peroxidase 4
ENSG00000167476	JSRP1	junctional sarcoplasmic reticulum protein 1
ENSG00000167487	KLHL26	kelch like family member 26
ENSG00000167508	MVD	mevalonate diphosphate decarboxylase
ENSG00000167535	CACNB3	calcium voltage-gated channel auxiliary subunit beta 3
ENSG00000167566	NCKAP5L	NCK associated protein 5 like
ENSG00000167595	PROSER3	proline and serine rich 3
ENSG00000167600	CYP2S1	cytochrome P450 family 2 subfamily S member 1
ENSG00000167613	LAIR1	leukocyte associated immunoglobulin like receptor 1
ENSG00000167614	TTYH1	tweety family member 1
ENSG00000167619	TMEM145	transmembrane protein 145
ENSG00000167625	ZNF526	zinc finger protein 526
ENSG00000167646	DNAAF3	dynein axonemal assembly factor 3
ENSG00000167685	ZNF444	zinc finger protein 444
ENSG00000167702	KIFC2	kinesin family member C2
ENSG00000167716	WDR81	WD repeat domain 81
ENSG00000167769	ACER1	alkaline ceramidase 1
ENSG00000167770	OTUB1	OTU deubiquitinase, ubiquitin aldehyde binding 1
ENSG00000167791	CABP2	calcium binding protein 2
ENSG00000167797	CDK2AP2	cyclin dependent kinase 2 associated protein 2
ENSG00000167798	C3P1	complement component 3 precursor pseudogene
ENSG00000167840	ZNF232	zinc finger protein 232
ENSG00000167850	CD300C	CD300c molecule
ENSG00000167861	HID1	HID1 domain containing
ENSG00000167880	EVPL	envoplakin
ENSG00000167912		novel transcript
ENSG00000167925	GHDC	GH3 domain containing
ENSG00000167962	ZNF598	zinc finger protein 598, E3 ubiquitin ligase
ENSG00000167968	DNASE1L2	deoxyribonuclease 1 like 2
ENSG00000167977	KCTD5	potassium channel tetramerization domain containing 5
ENSG00000167987	VPS37C	VPS37C subunit of ESCRT-I
ENSG00000167994	RAB3IL1	RAB3A interacting protein like 1
ENSG00000168002	POLR2G	RNA polymerase II subunit G
ENSG00000168005	SPINDOC	spindlin interactor and repressor of chromatin binding
ENSG00000168056	LTBP3	latent transforming growth factor beta binding protein 3
ENSG00000168060	NAALADL1	N-acetylated alpha-linked acidic dipeptidase like 1

ENSG00000168071	CCDC88B	coiled-coil domain containing 88B
ENSG00000168159	RNF187	ring finger protein 187
ENSG00000168256	NKIRAS2	NFKB inhibitor interacting Ras like 2
ENSG00000168264	IRF2BP2	interferon regulatory factor 2 binding protein 2
ENSG00000168268	NT5DC2	5'-nucleotidase domain containing 2
ENSG00000168269	FOXI1	forkhead box I1
ENSG00000168350	DEGS2	delta 4-desaturase, sphingolipid 2
ENSG00000168394	TAP1	transporter 1, ATP binding cassette subfamily B member
ENSG00000168397	ATG4B	autophagy related 4B cysteine peptidase
ENSG00000168418	KCNG4	potassium voltage-gated channel modifier subfamily G member 4
ENSG00000168439	STIP1	stress induced phosphoprotein 1
ENSG00000168453	HR	HR lysine demethylase and nuclear receptor corepressor
ENSG00000168476	REEP4	receptor accessory protein 4
ENSG00000168487	BMP1	bone morphogenetic protein 1
ENSG00000168505	GBX2	gastrulation brain homeobox 2
ENSG00000168509	HJV	hemojuvelin BMP co-receptor
ENSG00000168591	TMUB2	transmembrane and ubiquitin like domain containing 2
ENSG00000168621	GDNF	glial cell derived neurotrophic factor
ENSG00000168754	FAM178B	family with sequence similarity 178 member B
ENSG00000168906	MAT2A	methionine adenosyltransferase 2A
ENSG00000168907	PLA2G4F	phospholipase A2 group IVF
ENSG00000169021	UQCRFS1	ubiquinol-cytochrome c reductase, Rieske iron-sulfur polypeptide 1
ENSG00000169093	ASMTL	acetylserotonin O-methyltransferase like
ENSG00000169105	CHST14	carbohydrate sulfotransferase 14
ENSG00000169116	PARM1	prostate androgen-regulated mucin-like protein 1
ENSG00000169174	PCSK9	proprotein convertase subtilisin/kexin type 9
ENSG00000169188	APEX2	apurinic/apyrimidinic endodeoxyribonuclease 2
ENSG00000169189	NSMCE1	NSE1 homolog, SMC5-SMC6 complex component
ENSG00000169221	TBC1D10B	TBC1 domain family member 10B
ENSG00000169223	LMAN2	lectin, mannose binding 2
ENSG00000169230	PRELID1	PRELI domain containing 1
ENSG00000169245	CXCL10	C-X-C motif chemokine ligand 10
ENSG00000169248	CXCL11	C-X-C motif chemokine ligand 11
ENSG00000169253		ribosomal protein L36 (RPL36) pseudogene
ENSG00000169291	SHE	Src homology 2 domain containing E
ENSG00000169385	RNASE2	ribonuclease A family member 2
ENSG00000169439	SDC2	syndecan 2
ENSG00000169474	SPRR1A	small proline rich protein 1A
ENSG00000169515	CCDC8	coiled-coil domain containing 8
ENSG00000169564	PCBP1	poly(rC) binding protein 1
ENSG00000169683	LRRC45	leucine rich repeat containing 45
ENSG00000169696	ASPSCR1	ASPSCR1 tether for SLC2A4, UBX domain containing
ENSG00000169710	FASN	fatty acid synthase

ENSG00000169718	DUS1L	dihydrouridine synthase 1 like
ENSG00000169727	GPS1	G protein pathway suppressor 1
ENSG00000169733	RFNG	RFNG O-fucosylpeptide 3-beta-N-acetylglucosaminyltransferase
ENSG00000169738	DCXR	dicarbonyl and L-xylulose reductase
ENSG00000169750	RAC3	Rac family small GTPase 3
ENSG00000169856	ONECUT1	one cut homeobox 1
ENSG00000169951	ZNF764	zinc finger protein 764
ENSG00000169962	TAS1R3	taste 1 receptor member 3
ENSG00000169972	PUSL1	pseudouridine synthase like 1
ENSG00000170075	GPR37L1	G protein-coupled receptor 37 like 1
ENSG00000170088	TMEM192	transmembrane protein 192
ENSG00000170089		THO complex 3 (THOC3) pseudogene
ENSG00000170092	SPDYE5	speedy/RINGO cell cycle regulator family member E5
ENSG00000170266	GLB1	galactosidase beta 1
ENSG00000170323	FABP4	fatty acid binding protein 4
ENSG00000170345	FOS	Fos proto-oncogene, AP-1 transcription factor subunit
ENSG00000170348	TMED10	transmembrane p24 trafficking protein 10
ENSG00000170369	CST2	cystatin SA
ENSG00000170382	LRRN2	leucine rich repeat neuronal 2
ENSG00000170385	SLC30A1	solute carrier family 30 member 1
ENSG00000170425	ADORA2B	adenosine A2b receptor
ENSG00000170442	KRT86	keratin 86
ENSG00000170515	PA2G4	proliferation-associated 2G4
ENSG00000170561	IRX2	iroquois homeobox 2
ENSG00000170604	IRF2BP1	interferon regulatory factor 2 binding protein 1
ENSG00000170608	FOXA3	forkhead box A3
ENSG00000170684	ZNF296	zinc finger protein 296
ENSG00000170748	RBMXL2	RBMX like 2
ENSG00000170860	LSM3	LSM3 homolog, U6 small nuclear RNA and mRNA degradation associated
ENSG00000170876	TMEM43	transmembrane protein 43
ENSG00000170892	TSEN34	tRNA splicing endonuclease subunit 34
ENSG00000170893	TRH	thyrotropin releasing hormone
ENSG00000170906	NDUFA3	NADH:ubiquinone oxidoreductase subunit A3
ENSG00000171045	TSNARE1	t-SNARE domain containing 1
ENSG00000171119	NRTN	neurturin
ENSG00000171135	JAGN1	jagunal homolog 1
ENSG00000171159	C9orf16	chromosome 9 open reading frame 16
ENSG00000171219	CDC42BPG	CDC42 binding protein kinase gamma
ENSG00000171222	SCAND1	SCAN domain containing 1
ENSG00000171298	GAA	glucosidase alpha, acid
ENSG00000171302	CANT1	calcium activated nucleotidase 1
ENSG00000171307	ZDHHC16	zinc finger DHHC-type palmitoyltransferase 16

ENSG00000171311	EXOSC1	exosome component 1
ENSG00000171360	KRT38	keratin 38
ENSG00000171368	TPPP	tubulin polymerization promoting protein
ENSG00000171385	KCND3	potassium voltage-gated channel subfamily D member 3
ENSG00000171388	APLN	apelin
ENSG00000171401	KRT13	keratin 13
ENSG00000171443	ZNF524	zinc finger protein 524
ENSG00000171450	CDK5R2	cyclin dependent kinase 5 regulatory subunit 2
ENSG00000171475	WIPF2	WAS/WASL interacting protein family member 2
ENSG00000171496	OR1L8	olfactory receptor family 1 subfamily L member 8
ENSG00000171695	LKAAEAR1	LKAAEAR motif containing 1
ENSG00000171729	TMEM51	transmembrane protein 51
ENSG00000171773	NXNL1	nucleoredoxin like 1
ENSG00000171798	KNDC1	kinase non-catalytic C-lobe domain containing 1
ENSG00000171823	FBXL14	F-box and leucine rich repeat protein 14
ENSG00000171873	ADRA1D	adrenoceptor alpha 1D
ENSG00000171903	CYP4F11	cytochrome P450 family 4 subfamily F member 11
ENSG00000171914	TLN2	talin 2
ENSG00000172155	LCE1D	late cornified envelope 1D
ENSG00000172216	CEBPB	CCAAT enhancer binding protein beta
ENSG00000172247	C1QTNF4	C1q and TNF related 4
ENSG00000172250	SERHL	serine hydrolase like (pseudogene)
ENSG00000172269	DPAGT1	dolichyl-phosphate N-acetylglucosaminephosphotransferase 1
ENSG00000172270	BSG	basigin (Ok blood group)
ENSG00000172301	COPRS	coordinator of PRMT5 and differentiation stimulator
ENSG00000172375	C2CD2L	C2CD2 like
ENSG00000172409	CLP1	cleavage factor polyribonucleotide kinase subunit 1
ENSG00000172432	GTPBP2	GTP binding protein 2
ENSG00000172534	HCFC1	host cell factor C1
ENSG00000172538	FAM170B	family with sequence similarity 170 member B
ENSG00000172663	TMEM134	transmembrane protein 134
ENSG00000172680	MOS	MOS proto-oncogene, serine/threonine kinase
ENSG00000172782	FADS6	fatty acid desaturase 6
ENSG00000172830	SSH3	slingshot protein phosphatase 3
ENSG00000172831	CES2	carboxylesterase 2
ENSG00000172922	RNASEH2C	ribonuclease H2 subunit C
ENSG00000172932	ANKRD13D	ankyrin repeat domain 13D
ENSG00000172938	MRGPRD	MAS related GPR family member D
ENSG00000172971	UNC93B3	unc-93 homolog B3 (pseudogene)
ENSG00000172992	DCAKD	dephospho-CoA kinase domain containing
ENSG00000173065	FAM222B	family with sequence similarity 222 member B
ENSG00000173163	COMMD1	copper metabolism domain containing 1
ENSG00000173237	C11orf86	chromosome 11 open reading frame 86

ENSG00000173264	GPR137	G protein-coupled receptor 137
ENSG00000173391	OLR1	oxidized low density lipoprotein receptor 1
ENSG00000173511	VEGFB	vascular endothelial growth factor B
ENSG00000173517	PEAK1	pseudopodium enriched atypical kinase 1
ENSG00000173546	CSPG4	chondroitin sulfate proteoglycan 4
ENSG00000173548	SNX33	sorting nexin 33
ENSG00000173557	FAM166C	family with sequence similarity 166 member C
ENSG00000173578	XCR1	X-C motif chemokine receptor 1
ENSG00000173581	CCDC106	coiled-coil domain containing 106
ENSG00000173673	HES3	hes family bHLH transcription factor 3
ENSG00000173705	SUSD5	sushi domain containing 5
ENSG00000173769	TOPAZ1	testis and ovary specific PAZ domain containing 1
ENSG00000173786	CNP	2',3'-cyclic nucleotide 3' phosphodiesterase
ENSG00000173818	ENDOV	endonuclease V
ENSG00000173868	PHOSPHO1	phosphoethanolamine/phosphocholine phosphatase 1
ENSG00000173898	SPTBN2	spectrin beta, non-erythrocytic 2
ENSG00000173915	ATP5MD	ATP synthase membrane subunit DAPIT
ENSG00000173917	HOXB2	homeobox B2
ENSG00000173947	PIFO	primary cilia formation
ENSG00000174080	CTSF	cathepsin F
ENSG00000174233	ADCY6	adenylate cyclase 6
ENSG00000174236	REP15	RAB15 effector protein
ENSG00000174279	EVX2	even-skipped homeobox 2
ENSG00000174282	ZBTB4	zinc finger and BTB domain containing 4
ENSG00000174327	SLC16A13	solute carrier family 16 member 13
ENSG00000174403	MIR1-1HG-AS1	MIR1-1HG antisense RNA 1
ENSG00000174429	ABRA	actin binding Rho activating protein
ENSG00000174498	IGDCC3	immunoglobulin superfamily DCC subclass member 3
ENSG00000174516	PELI3	pellino E3 ubiquitin protein ligase family member 3
ENSG00000174667	OR7D4	olfactory receptor family 7 subfamily D member 4
ENSG00000174749	FAM241A	family with sequence similarity 241 member A
ENSG00000174775	HRAS	HRas proto-oncogene, GTPase
ENSG00000174791	RIN1	Ras and Rab interactor 1
ENSG00000174851	YIF1A	Yip1 interacting factor homolog A, membrane trafficking protein
ENSG00000174871	CNIH2	cornichon family AMPA receptor auxiliary protein 2
ENSG00000174903	RAB1B	RAB1B, member RAS oncogene family
ENSG00000174943	KCTD13	potassium channel tetramerization domain containing 13
ENSG00000175084	DES	desmin
ENSG00000175203	DCTN2	dynactin subunit 2
ENSG00000175283	DOLK	dolichol kinase
ENSG00000175294	CATSPER1	cation channel sperm associated 1
ENSG00000175315	CST6	cystatin E/M
ENSG00000175348	TMEM9B	TMEM9 domain family member B

ENSG00000175390	EIF3F	eukaryotic translation initiation factor 3 subunit F
ENSG00000175556	LONRF3	LON peptidase N-terminal domain and ring finger 3
ENSG00000175573	C11orf68	chromosome 11 open reading frame 68
ENSG00000175582	RAB6A	RAB6A, member RAS oncogene family
ENSG00000175592	FOSL1	FOS like 1, AP-1 transcription factor subunit
ENSG00000175634	RPS6KB2	ribosomal protein S6 kinase B2
ENSG00000175646	PRM1	protamine 1
ENSG00000175826	CTDNEP1	CTD nuclear envelope phosphatase 1
ENSG00000175866	BAIAP2	BAR/IMD domain containing adaptor protein 2
ENSG00000176046	NUPR1	nuclear protein 1, transcriptional regulator
ENSG00000176087	SLC35A4	solute carrier family 35 member A4
ENSG00000176101	SSNA1	SS nuclear autoantigen 1
ENSG00000176153	GPX2	glutathione peroxidase 2
ENSG00000176165	FOXG1	forkhead box G1
ENSG00000176170	SPHK1	sphingosine kinase 1
ENSG00000176182	MYPOP	Myb related transcription factor, partner of profilin
ENSG00000176183		actin, beta (ACTB) pseudogene
ENSG00000176243	CDV3P1	CDV3 pseudogene 1
ENSG00000176349		novel transcript, antisense to MAD1L1
ENSG00000176381	PRR18	proline rich 18
ENSG00000176387	HSD11B2	hydroxysteroid 11-beta dehydrogenase 2
ENSG00000176410	DNAJC30	DnaJ heat shock protein family (Hsp40) member C30
ENSG00000176444	CLK2	CDC like kinase 2
ENSG00000176472	ZNF575	zinc finger protein 575
ENSG00000176510	OR10AC1	olfactory receptor family 10 subfamily AC member 1 (gene/pseudogene)
ENSG00000176531	PHLDB3	pleckstrin homology like domain family B member 3
ENSG00000176753	C15orf56	chromosome 15 open reading frame 56
ENSG00000176769	TCERG1L	transcription elongation regulator 1 like
ENSG00000176840	MIR7-3HG	MIR7-3 host gene
ENSG00000176882		novel pseudogene
ENSG00000176887	SOX11	SRY-box transcription factor 11
ENSG00000176909	MAMSTR	MEF2 activating motif and SAP domain containing transcriptional regulator
ENSG00000176919	C8G	complement C8 gamma chain
ENSG00000176956	LY6H	lymphocyte antigen 6 family member H
ENSG00000176978	DPP7	dipeptidyl peptidase 7
ENSG00000176994	SMCR8	SMCR8-C9orf72 complex subunit
ENSG00000177045	SIX5	SIX homeobox 5
ENSG00000177051	FBXO46	F-box protein 46
ENSG00000177105	RHOG	ras homolog family member G
ENSG00000177234	LINC01561	long intergenic non-protein coding RNA 1561
ENSG00000177238	TRIM72	tripartite motif containing 72
ENSG00000177283	FZD8	frizzled class receptor 8

ENSG00000177369		uncharacterized FLJ40194 [Source:NCBI gene (formerly Entrezgene);Acc:124871]
ENSG00000177370	TIMM22	translocase of inner mitochondrial membrane 22
ENSG00000177398	UMODL1	uromodulin like 1
ENSG00000177462	OR2T8	olfactory receptor family 2 subfamily T member 8
ENSG00000177485	ZBTB33	zinc finger and BTB domain containing 33
ENSG00000177508	IRX3	iroquois homeobox 3
ENSG00000177575	CD163	CD163 molecule
ENSG00000177595	PIDD1	p53-induced death domain protein 1
ENSG00000177600	RPLP2	ribosomal protein lateral stalk subunit P2
ENSG00000177666	PNPLA2	patatin like phospholipase domain containing 2
ENSG00000177679	SRRM3	serine/arginine repetitive matrix 3
ENSG00000177685	CRACR2B	calcium release activated channel regulator 2B
ENSG00000177689	MAGEB10	MAGE family member B10
ENSG00000177706	FAM20C	FAM20C golgi associated secretory pathway kinase
ENSG00000177728	TMEM94	transmembrane protein 94
ENSG00000177791	MYOZ1	myozenin 1
ENSG00000177803	YWHAQP4	YWHAQ pseudogene 4
ENSG00000177822	TENM3-AS1	TENM3 antisense RNA 1
ENSG00000177879	AP3S1	adaptor related protein complex 3 subunit sigma 1
ENSG00000177943	MAMDC4	MAM domain containing 4
ENSG00000177951	BET1L	Bet1 golgi vesicular membrane trafficking protein like
ENSG00000177989	ODF3B	outer dense fiber of sperm tails 3B
ENSG00000178015	GPR150	G protein-coupled receptor 150
ENSG00000178057	NDUFAF3	NADH:ubiquinone oxidoreductase complex assembly factor 3
ENSG00000178188	SH2B1	SH2B adaptor protein 1
ENSG00000178199	ZC3H12D	zinc finger CCCH-type containing 12D
ENSG00000178279	TNP2	transition protein 2
ENSG00000178372	CALML5	calmodulin like 5
ENSG00000178403	NEUROG2	neurogenin 2
ENSG00000178464	RPL10P16	ribosomal protein L10 pseudogene 16
ENSG00000178467	P4HTM	prolyl 4-hydroxylase, transmembrane
ENSG00000178556	CKS1BP6	CDC28 protein kinase regulatory subunit 1B pseudogene 6
ENSG00000178597	PSAPL1	prosaposin like 1
ENSG00000178654	PPIAP33	peptidylprolyl isomerase A pseudogene 33
ENSG00000178695	KCTD12	potassium channel tetramerization domain containing 12
ENSG00000178723	GLULP4	glutamate-ammonia ligase pseudogene 4
ENSG00000178732	GP5	glycoprotein V platelet
ENSG00000178741	COX5A	cytochrome c oxidase subunit 5A
ENSG00000178762	H2BC2P	H2B clustered histone 2, pseudogene
ENSG00000178772	CPN2	carboxypeptidase N subunit 2
ENSG00000178803	ADORA2A-AS1	ADORA2A antisense RNA 1
ENSG00000178882	RFLNA	refilin A

ENSG00000178950	GAK	cyclin G associated kinase
ENSG00000178951	ZBTB7A	zinc finger and BTB domain containing 7A
ENSG00000178980	SELENOW	selenoprotein W
ENSG00000178982	EIF3K	eukaryotic translation initiation factor 3 subunit K
ENSG00000179038		tetratricopeptide repeat domain 3 (TTC3) pseudogene
ENSG00000179051	RCC2	regulator of chromosome condensation 2
ENSG00000179058	C9orf50	chromosome 9 open reading frame 50
ENSG00000179094	PER1	period circadian regulator 1
ENSG00000179111	HES7	hes family bHLH transcription factor 7
ENSG00000179163	FUCA1	alpha-L-fucosidase 1
ENSG00000179172	HNRNPCL1	heterogeneous nuclear ribonucleoprotein C like 1
ENSG00000179242	CDH4	cadherin 4
ENSG00000179331	RAB39A	RAB39A, member RAS oncogene family
ENSG00000179342		guanine nucleotide binding protein (G protein), alpha 11 (Gq class) (GNA11) pseudogene
ENSG00000179431	FJX1	four-jointed box kinase 1
ENSG00000179447	SLC24A3-AS1	SLC24A3 antisense RNA 1
ENSG00000179528	LBX2	ladybird homeobox 2
ENSG00000179588	ZFPM1	zinc finger protein, FOG family member 1
ENSG00000179632	MAF1	MAF1 homolog, negative regulator of RNA polymerase III
ENSG00000179636	TPPP2	tubulin polymerization promoting protein family member 2
ENSG00000179639	FCER1A	Fc fragment of IgE receptor Ia
ENSG00000179743		uncharacterized LOC729614 [Source:NCBI gene (formerly Entrezgene);Acc:729614]
ENSG00000179774	ATOH7	atonal bHLH transcription factor 7
ENSG00000179796	LRRC3B	leucine rich repeat containing 3B
ENSG00000179799	OR7E22P	olfactory receptor family 7 subfamily E member 22 pseudogene
ENSG00000179886	TIGD5	tigger transposable element derived 5
ENSG00000179921	GPBAR1	G protein-coupled bile acid receptor 1
ENSG00000179933	C14orf119	chromosome 14 open reading frame 119
ENSG00000179994	SPDYE7P	speedy/RINGO cell cycle regulator family member E7, pseudogene
ENSG00000180043	FAM71E2	family with sequence similarity 71 member E2
ENSG00000180053	NKX2-6	NK2 homeobox 6
ENSG00000180138	CSNK1A1L	casein kinase 1 alpha 1 like
ENSG00000180190	TDRP	testis development related protein
ENSG00000180209	MYLPF	myosin light chain, phosphorylatable, fast skeletal muscle
ENSG00000180251	SLC9A4	solute carrier family 9 member A4
ENSG00000180264	ADGRD2	adhesion G protein-coupled receptor D2
ENSG00000180269	GPR139	G protein-coupled receptor 139
ENSG00000180304	OAZ2	ornithine decarboxylase antizyme 2
ENSG00000180398	MCFD2	multiple coagulation factor deficiency 2, ER cargo receptor complex subunit
ENSG00000180438	TPRXL	tetrapeptide repeat homeobox like (pseudogene)
ENSG00000180447	GAS1	growth arrest specific 1
ENSG00000180610	ZBTB12BP	zinc finger and BTB domain containing 12B, pseudogene

ENSG00000180660	MAB21L1	mab-21 like 1
ENSG00000180663	VN1R3	vomeronasal 1 receptor 3
ENSG00000180767	CHST13	carbohydrate sulfotransferase 13
ENSG00000180769	WDFY3-AS2	WDFY3 antisense RNA 2
ENSG00000180879	SSR4	signal sequence receptor subunit 4
ENSG00000180900	SCRIB	scribble planar cell polarity protein
ENSG00000180921	FAM83H	family with sequence similarity 83 member H
ENSG00000181045	SLC26A11	solute carrier family 26 member 11
ENSG00000181123		novel transcript, antisense to SEC14L4
ENSG00000181126	HLA-V	major histocompatibility complex, class I, V (pseudogene)
ENSG00000181195	PENK	proenkephalin
ENSG00000181201	H2BU2P	H2B.U histone 2, pseudogene
ENSG00000181264	TLCD5	TLC domain containing 5
ENSG00000181409	AATK	apoptosis associated tyrosine kinase
ENSG00000181523	SGSH	N-sulfoglucosamine sulfohydrolase
ENSG00000181773	GPR3	G protein-coupled receptor 3
ENSG00000181781	ODF3L2	outer dense fiber of sperm tails 3 like 2
ENSG00000181786	ACTL9	actin like 9
ENSG00000181790	ADGRB1	adhesion G protein-coupled receptor B1
ENSG00000181800	CELF2-AS1	CELF2 antisense RNA 1
ENSG00000181830	SLC35C1	solute carrier family 35 member C1
ENSG00000181885	CLDN7	claudin 7
ENSG00000181965	NEUROG1	neurogenin 1
ENSG00000181982	CCDC149	coiled-coil domain containing 149
ENSG00000181991	MRPS11	mitochondrial ribosomal protein S11
ENSG00000182087	TMEM259	transmembrane protein 259
ENSG00000182095	TNRC18	trinucleotide repeat containing 18
ENSG00000182103	FAM181B	family with sequence similarity 181 member B
ENSG00000182117	NOP10	NOP10 ribonucleoprotein
ENSG00000182175	RGMA	repulsive guidance molecule BMP co-receptor a
ENSG00000182220	ATP6AP2	ATPase H+ transporting accessory protein 2
ENSG00000182223	ZAR1	zygote arrest 1
ENSG00000182257	PRR34	PRR34 long non-coding RNA
ENSG00000182325	FBXL6	F-box and leucine rich repeat protein 6
ENSG00000182327	GLTPD2	glycolipid transfer protein domain containing 2
ENSG00000182351	CRIP1P4	cysteine rich protein 1 pseudogene 4
ENSG00000182372	CLN8	CLN8 transmembrane ER and ERGIC protein
ENSG00000182389	CACNB4	calcium voltage-gated channel auxiliary subunit beta 4
ENSG00000182544	MFSD5	major facilitator superfamily domain containing 5
ENSG00000182575	NXPH3	neurexophilin 3
ENSG00000182578	CSF1R	colony stimulating factor 1 receptor
ENSG00000182580	EPHB3	EPH receptor B3

ENSG00000182600	SNORC	secondary ossification center associated regulator of chondrocyte maturation
ENSG00000182612	TSPAN10	tetraspanin 10
ENSG00000182759	MAFA	MAF bZIP transcription factor A
ENSG00000182795	C1orf116	chromosome 1 open reading frame 116
ENSG00000182796	TMEM198B	transmembrane protein 198B (pseudogene)
ENSG00000182853	VMO1	vitelline membrane outer layer 1 homolog
ENSG00000182899	RPL35A	ribosomal protein L35a
ENSG00000182938	OTOP3	otopetrin 3
ENSG00000182979	MTA1	metastasis associated 1
ENSG00000183034	OTOP2	otopetrin 2
ENSG00000183128	CALHM3	calcium homeostasis modulator 3
ENSG00000183146	PRORY	proline rich Y-linked
ENSG00000183153	GJD3	gap junction protein delta 3
ENSG00000183154		novel transcript, antisense to ERLIN2
ENSG00000183248	PRR36	proline rich 36
ENSG00000183258	DDX41	DEAD-box helicase 41
ENSG00000183283	DAZAP2	DAZ associated protein 2
ENSG00000183291	SELENOF	selenoprotein F
ENSG00000183496	MEX3B	mex-3 RNA binding family member B
ENSG00000183597	TANGO2	transport and golgi organization 2 homolog
ENSG00000183615	FAM167B	family with sequence similarity 167 member B
ENSG00000183741	CBX6	chromobox 6
ENSG00000183770	FOXL2	forkhead box L2
ENSG00000183773	AIFM3	apoptosis inducing factor mitochondria associated 3
ENSG00000183784	DOCK8-AS1	DOCK8 antisense RNA 1
ENSG00000183798	EMILIN3	elastin microfibril interfacer 3
ENSG00000183807	FAM162B	family with sequence similarity 162 member B
ENSG00000183822	NCF4-AS1	NCF4 antisense RNA 1
ENSG00000183828	NUDT14	nudix hydrolase 14
ENSG00000183888	SRARP	steroid receptor associated and regulated protein
ENSG00000183908	LRRC55	leucine rich repeat containing 55
ENSG00000183971	NPW	neuropeptide W
ENSG00000183979	NPB	neuropeptide B
ENSG00000184007	PTP4A2	protein tyrosine phosphatase 4A2
ENSG00000184058	TBX1	T-box transcription factor 1
ENSG00000184076	UQCR10	ubiquinol-cytochrome c reductase, complex III subunit X
ENSG00000184144	CNTN2	contactin 2
ENSG00000184148	SPRR4	small proline rich protein 4
ENSG00000184160	ADRA2C	adrenoceptor alpha 2C
ENSG00000184164	CRELD2	cysteine rich with EGF like domains 2
ENSG00000184188		actin, beta (ACTB) pseudogene
ENSG00000184216	IRAK1	interleukin 1 receptor associated kinase 1

ENSG00000184270	H2AC21	H2A clustered histone 21
ENSG00000184271	POU6F1	POU class 6 homeobox 1
ENSG00000184350	MRGPRE	MAS related GPR family member E
ENSG00000184357	H1-5	H1.5 linker histone, cluster member
ENSG00000184423	RPL23AP38	ribosomal protein L23a pseudogene 38
ENSG00000184436	THAP7	THAP domain containing 7
ENSG00000184441		novel transcript, antisense to C21orf2
ENSG00000184471	C1QTNF8	C1q and TNF related 8
ENSG00000184486	POU3F2	POU class 3 homeobox 2
ENSG00000184497	TMEM255B	transmembrane protein 255B
ENSG00000184508	HDDC3	HD domain containing 3
ENSG00000184601	C14orf180	chromosome 14 open reading frame 180
ENSG00000184678	H2BC21	H2B clustered histone 21
ENSG00000184702	SEPTIN5	septin 5
ENSG00000184731	FAM110C	family with sequence similarity 110 member C
ENSG00000184792	OSBP2	oxysterol binding protein 2
ENSG00000184831	APOO	apolipoprotein O
ENSG00000184887	BTBD6	BTB domain containing 6
ENSG00000184897	H1-10	H1.10 linker histone
ENSG00000184922	FMNL1	formin like 1
ENSG00000184937	WT1	WT1 transcription factor
ENSG00000184945	AQP12A	aquaporin 12A
ENSG00000184956	MUC6	mucin 6, oligomeric mucus/gel-forming
ENSG00000184985	SORCS2	sortilin related VPS10 domain containing receptor 2
ENSG00000184986	TMEM121	transmembrane protein 121
ENSG00000184988	TMEM106A	transmembrane protein 106A
ENSG00000184990	SIVA1	SIVA1 apoptosis inducing factor
ENSG00000185000	DGAT1	diacylglycerol O-acyltransferase 1
ENSG00000185049	NELFA	negative elongation factor complex member A
ENSG00000185070	FLRT2	fibronectin leucine rich transmembrane protein 2
ENSG00000185122	HSF1	heat shock transcription factor 1
ENSG00000185130	H2BC13	H2B clustered histone 13
ENSG00000185189	NRBP2	nuclear receptor binding protein 2
ENSG00000185198	PRSS57	serine protease 57
ENSG00000185296		aurora kinase A interacting protein 1 (AURKAIP1) pseudogene
ENSG00000185339	TCN2	transcobalamin 2
ENSG00000185359	HGS	hepatocyte growth factor-regulated tyrosine kinase substrate
ENSG00000185361	TNFAIP8L1	TNF alpha induced protein 8 like 1
ENSG00000185448	FAM47A	family with sequence similarity 47 member A
ENSG00000185453	ZSWIM9	zinc finger SWIM-type containing 9
ENSG00000185475	TMEM179B	transmembrane protein 179B
ENSG00000185522	LMNTD2	lamin tail domain containing 2
ENSG00000185527	PDE6G	phosphodiesterase 6G

ENSG00000185561	TLCD2	TLC domain containing 2
ENSG00000185565	LSAMP	limbic system associated membrane protein
ENSG00000185585	OLFML2A	olfactomedin like 2A
ENSG00000185641		ribosomal protein S25 (RPS25) pseudogene
ENSG00000185650	ZFP36L1	ZFP36 ring finger protein like 1
ENSG00000185666	SYN3	synapsin III
ENSG00000185689	C6orf201	chromosome 6 open reading frame 201
ENSG00000185739	SRL	sarcalumenin
ENSG00000185742	C11orf87	chromosome 11 open reading frame 87
ENSG00000185761	ADAMTSL5	ADAMTS like 5
ENSG00000185825	BCAP31	B cell receptor associated protein 31
ENSG00000185838	GNB1L	G protein subunit beta 1 like
ENSG00000185896	LAMP1	lysosomal associated membrane protein 1
ENSG00000185909	KLHDC8B	kelch domain containing 8B
ENSG00000186008	RPS4XP21	ribosomal protein S4X pseudogene 21
ENSG00000186082	KRT18P14	keratin 18 pseudogene 14
ENSG00000186115	CYP4F2	cytochrome P450 family 4 subfamily F member 2
ENSG00000186160	CYP4Z1	cytochrome P450 family 4 subfamily Z member 1
ENSG00000186207	LCE5A	late cornified envelope 5A
ENSG00000186234	FAM86MP	family with sequence similarity 86 member M, pseudogene
ENSG00000186283	TOR3A	torsin family 3 member A
ENSG00000186318	BACE1	beta-secretase 1
ENSG00000186326	RGS9BP	regulator of G protein signaling 9 binding protein
ENSG00000186340	THBS2	thrombospondin 2
ENSG00000186468	RPS23	ribosomal protein S23
ENSG00000186564	FOXD2	forkhead box D2
ENSG00000186567	CEACAM19	CEA cell adhesion molecule 19
ENSG00000186766	FOXI2	forkhead box I2
ENSG00000186825	CDRT15P3	CDRT15 pseudogene 3
ENSG00000186832	KRT16	keratin 16
ENSG00000186862	PDZD7	PDZ domain containing 7
ENSG00000186940	CHCHD2P9	coiled-coil-helix-coiled-coil-helix domain containing 2 pseudogene 9
ENSG00000186994	KANK3	KN motif and ankyrin repeat domains 3
ENSG00000187024	PTRH1	peptidyl-tRNA hydrolase 1 homolog
ENSG00000187049	TMEM216	transmembrane protein 216
ENSG00000187068	C3orf70	chromosome 3 open reading frame 70
ENSG00000187098	MITF	melanocyte inducing transcription factor
ENSG00000187135	VSTM2B	V-set and transmembrane domain containing 2B
ENSG00000187140	FOXD3	forkhead box D3
ENSG00000187144	SPATA21	spermatogenesis associated 21
ENSG00000187147	RNF220	ring finger protein 220
ENSG00000187166	H1-7	H1.7 linker histone
ENSG00000187173	LCE2A	late cornified envelope 2A

ENSG00000187238	LCE3B	late cornified envelope 3B
ENSG00000187446	CHP1	calcineurin like EF-hand protein 1
ENSG00000187474	FPR3	formyl peptide receptor 3
ENSG00000187534	PRR13P5	proline rich 13 pseudogene 5
ENSG00000187634	SAMD11	sterile alpha motif domain containing 11
ENSG00000187730	GABRD	gamma-aminobutyric acid type A receptor subunit delta
ENSG00000187800	PEAR1	platelet endothelial aggregation receptor 1
ENSG00000187821	HELT	helt bHLH transcription factor
ENSG00000187997	C17orf99	chromosome 17 open reading frame 99
ENSG00000188032	C19orf67	chromosome 19 open reading frame 67
ENSG00000188070	C11orf95	chromosome 11 open reading frame 95
ENSG00000188078		Hermansky-Pudlak syndrome 1 (HPS1) pseudogene
ENSG00000188130	MAPK12	mitogen-activated protein kinase 12
ENSG00000188176	SMTNL2	smoothelin like 2
ENSG00000188263	IL17REL	interleukin 17 receptor E like
ENSG00000188290	HES4	hes family bHLH transcription factor 4
ENSG00000188305	PEAK3	PEAK family member 3
ENSG00000188315	C3orf62	chromosome 3 open reading frame 62
ENSG00000188385	JAKMIP3	Janus kinase and microtubule interacting protein 3
ENSG00000188459	WASF4P	WASP family member 4, pseudogene
ENSG00000188542	DUSP28	dual specificity phosphatase 28
ENSG00000188585	CLEC20A	C-type lectin domain containing 20A
ENSG00000188613	NANOS1	nanos C2HC-type zinc finger 1
ENSG00000188620	HMX3	H6 family homeobox 3
ENSG00000188662	H1-9P	H1.9 linker histone, pseudogene
ENSG00000188763	FZD9	frizzled class receptor 9
ENSG00000189001	SBSN	suprabasin
ENSG00000196123	KIAA0895L	KIAA0895 like
ENSG00000196132	MYT1	myelin transcription factor 1
ENSG00000196169	KIF19	kinesin family member 19
ENSG00000196182	STK40	serine/threonine kinase 40
ENSG00000196224	KRTAP5-3	keratin associated protein 5-3
ENSG00000196296	ATP2A1	ATPase sarcoplasmic/endoplasmic reticulum Ca2+ transporting 1
ENSG00000196301	HLA-DRB9	major histocompatibility complex, class II, DR beta 9 (pseudogene)
ENSG00000196313	POM121	POM121 transmembrane nucleoporin
ENSG00000196364	PRSS29P	serine protease 29, pseudogene
ENSG00000196388	INCA1	inhibitor of CDK, cyclin A1 interacting protein 1
ENSG00000196395		GAGE family pseudogene
ENSG00000196421	C20orf204	chromosome 20 open reading frame 204
ENSG00000196431	CRYBA4	crystallin beta A4
ENSG00000196507	TCEAL3	transcription elongation factor A like 3
ENSG00000196547	MAN2A2	mannosidase alpha class 2A member 2
ENSG00000196557	CACNA1H	calcium voltage-gated channel subunit alpha1 H

ENSG00000196734	LCE1B	late cornified envelope 1B
ENSG00000196739	COL27A1	collagen type XXVII alpha 1 chain
ENSG00000196748	CLPSL2	colipase like 2
ENSG00000196811	CHRNG	cholinergic receptor nicotinic gamma subunit
ENSG00000196961	AP2A1	adaptor related protein complex 2 subunit alpha 1
ENSG00000196979	GPRACR	GPR107 adjacent cis regulating lncRNA
ENSG00000196990	FAM163B	family with sequence similarity 163 member B
ENSG00000197067	OR2T32P	olfactory receptor family 2 subfamily T member 32 pseudogene
ENSG00000197070	ARRDC1	arrestin domain containing 1
ENSG00000197114	ZGPAT	zinc finger CCCH-type and G-patch domain containing
ENSG00000197136	PCNX3	pecanex 3
ENSG00000197162	ZNF785	zinc finger protein 785
ENSG00000197180		uncharacterized protein BC009467 [Source:NCBI gene (formerly Entrezgene);Acc:158960]
ENSG00000197226	TBC1D9B	TBC1 domain family member 9B
ENSG00000197245	FAM110D	family with sequence similarity 110 member D
ENSG00000197283	SYNGAP1	synaptic Ras GTPase activating protein 1
ENSG00000197324	LRP10	LDL receptor related protein 10
ENSG00000197353	LYPD2	LY6/PLAUR domain containing 2
ENSG00000197380	DACT3	dishevelled binding antagonist of beta catenin 3
ENSG00000197405	C5AR1	complement C5a receptor 1
ENSG00000197457	STMN3	stathmin 3
ENSG00000197467	COL13A1	collagen type XIII alpha 1 chain
ENSG00000197487	GALP	galanin like peptide
ENSG00000197530	MIB2	mindbomb E3 ubiquitin protein ligase 2
ENSG00000197558	SSPOP	SCO-spondin, pseudogene
ENSG00000197746	PSAP	prosaposin
ENSG00000197747	S100A10	S100 calcium binding protein A10
ENSG00000197766	CFD	complement factor D
ENSG00000197798	FAM118B	family with sequence similarity 118 member B
ENSG00000197813		novel transcript
ENSG00000197921	HES5	hes family bHLH transcription factor 5
ENSG00000197982	C1orf122	chromosome 1 open reading frame 122
ENSG00000198003	CCDC151	coiled-coil domain containing 151
ENSG00000198053	SIRPA	signal regulatory protein alpha
ENSG00000198090	KRTAP4-6	keratin associated protein 4-6
ENSG00000198156	NPIPB6	nuclear pore complex interacting protein family member B6
ENSG00000198173	FAM47C	family with sequence similarity 47 member C
ENSG00000198189	HSD17B11	hydroxysteroid 17-beta dehydrogenase 11
ENSG00000198208	RPS6KL1	ribosomal protein S6 kinase like 1
ENSG00000198225	FKBP1C	FKBP prolyl isomerase 1C
ENSG00000198246	SLC29A3	solute carrier family 29 member 3
ENSG00000198276	UCKL1	uridine-cytidine kinase 1 like 1

ENSG00000198324	PHETA1	PH domain containing endocytic trafficking adaptor 1
ENSG00000198354	DCAF12L2	DDB1 and CUL4 associated factor 12 like 2
ENSG00000198454	LINC02692	long intergenic non-protein coding RNA 2692
ENSG00000198492	YTHDF2	YTH N6-methyladenosine RNA binding protein 2
ENSG00000198526	PABPC1P2	poly(A) binding protein cytoplasmic 1 pseudogene 2
ENSG00000198535	C2CD4A	C2 calcium dependent domain containing 4A
ENSG00000198569	SLC34A3	solute carrier family 34 member 3
ENSG00000198576	ARC	activity regulated cytoskeleton associated protein
ENSG00000198598	MMP17	matrix metallopeptidase 17
ENSG00000198711	SSBP3-AS1	SSBP3 antisense RNA 1
ENSG00000198715	GLMP	glycosylated lysosomal membrane protein
ENSG00000198768	APCDD1L	APC down-regulated 1 like
ENSG00000198816	ZNF358	zinc finger protein 358
ENSG00000198824	CHAMP1	chromosome alignment maintaining phosphoprotein 1
ENSG00000198832	SELENOM	selenoprotein M
ENSG00000198837	DENND4B	DENN domain containing 4B
ENSG00000198842	STYXL2	serine/threonine/tyrosine interacting like 2
ENSG00000198843	SELENOT	selenoprotein T
ENSG00000198853	RUSC2	RUN and SH3 domain containing 2
ENSG00000198889	DCAF12L1	DDB1 and CUL4 associated factor 12 like 1
ENSG00000198911	SREBF2	sterol regulatory element binding transcription factor 2
ENSG00000198925	ATG9A	autophagy related 9A
ENSG00000198931	APRT	adenine phosphoribosyltransferase
ENSG00000198960	ARMCX6	armadillo repeat containing X-linked 6
ENSG00000199036	MIR219A1	microRNA 219a-1
ENSG00000199490		Y RNA [Source:RFAM;Acc:RF00019]
ENSG00000199509	RNA5SP477	RNA, 5S ribosomal pseudogene 477
ENSG00000199565		Y RNA [Source:RFAM;Acc:RF00019]
ENSG00000199572	RNA5SP174	RNA, 5S ribosomal pseudogene 174
ENSG00000199638	RNA5SP319	RNA, 5S ribosomal pseudogene 319
ENSG00000199687	RNU1-38P	RNA, U1 small nuclear 38, pseudogene
ENSG00000199732		Y RNA [Source:RFAM;Acc:RF00019]
ENSG00000199831	RN7SKP291	RN7SK pseudogene 291
ENSG00000199845	RNA5SP375	RNA, 5S ribosomal pseudogene 375
ENSG00000199859	RNU6-582P	RNA, U6 small nuclear 582, pseudogene
ENSG00000199866		Y RNA [Source:RFAM;Acc:RF00019]
ENSG00000199883	RN7SKP90	RN7SK pseudogene 90
ENSG00000200011		Y RNA [Source:RFAM;Acc:RF00019]
ENSG00000200041		Y RNA [Source:RFAM;Acc:RF00019]
ENSG00000200097	RNU6-1167P	RNA, U6 small nuclear 1167, pseudogene
ENSG00000200101	RNU6-508P	RNA, U6 small nuclear 508, pseudogene
ENSG00000200206		
ENSG00000200238	RNA5SP133	RNA, 5S ribosomal pseudogene 133

ENSG00000200332		Y RNA [Source:RFAM;Acc:RF00019]
ENSG00000200361		Y RNA [Source:RFAM;Acc:RF00019]
ENSG00000200408	RNA5SP74	RNA, 5S ribosomal pseudogene 74
ENSG00000200483	RNU6-1017P	RNA, U6 small nuclear 1017, pseudogene
ENSG00000200652		
ENSG00000200702		Y RNA [Source:RFAM;Acc:RF00019]
ENSG00000201035	RNA5SP469	RNA, 5S ribosomal pseudogene 469
ENSG00000201066	RN7SKP280	RN7SK pseudogene 280
ENSG00000201078	RN7SKP214	RN7SK pseudogene 214
ENSG00000201162	RNU6-454P	RNA, U6 small nuclear 454, pseudogene
ENSG00000201216		Y RNA [Source:RFAM;Acc:RF00019]
ENSG00000201330	SNORD32B	small nucleolar RNA, C/D box 32B
ENSG00000201368		Small nucleolar RNA U3 [Source:RFAM;Acc:RF00012]
ENSG00000201390	RNU6-1141P	RNA, U6 small nuclear 1141, pseudogene
ENSG00000201415	RNA5SP204	RNA, 5S ribosomal pseudogene 204
ENSG00000201426		Y RNA [Source:RFAM;Acc:RF00019]
ENSG00000201447	RNA5SP509	RNA, 5S ribosomal pseudogene 509
ENSG00000201451		Y RNA [Source:RFAM;Acc:RF00019]
ENSG00000201535		Y RNA [Source:RFAM;Acc:RF00019]
ENSG00000201584		Y RNA [Source:RFAM;Acc:RF00019]
ENSG00000201612	RN7SKP15	RN7SK pseudogene 15
ENSG00000201624		Y RNA [Source:RFAM;Acc:RF00019]
ENSG00000201725	RNU6-304P	RNA, U6 small nuclear 304, pseudogene
ENSG00000201782	RN7SKP226	RN7SK pseudogene 226
ENSG00000202092	RNA5SP190	RNA, 5S ribosomal pseudogene 190
ENSG00000202260	RN7SKP69	RN7SK pseudogene 69
ENSG00000202318		Y RNA [Source:RFAM;Acc:RF00019]
ENSG00000202344	RN7SKP208	RN7SK pseudogene 208
ENSG00000202368		Y RNA [Source:RFAM;Acc:RF00019]
ENSG00000202461		Y RNA [Source:RFAM;Acc:RF00019]
ENSG00000203326	ZNF525	zinc finger protein 525
ENSG00000203690	TCP10L3	t-complex 10 like 3, pseudogene
ENSG00000203797	DDO	D-aspartate oxidase
ENSG00000203879	GDI1	GDP dissociation inhibitor 1
ENSG00000203950	RTL8A	retrotransposon Gag like 8A
ENSG00000203993	ARRDC1-AS1	ARRDC1 antisense RNA 1
ENSG00000204011	COL5A1-AS1	COL5A1 antisense RNA 1
ENSG00000204070	SYS1	SYS1 golgi trafficking protein
ENSG00000204099	NEU4	neuraminidase 4
ENSG00000204219	TCEA3	transcription elongation factor A3
ENSG00000204220	PFDN6	prefoldin subunit 6
ENSG00000204256	BRD2	bromodomain containing 2
ENSG00000204291	COL15A1	collagen type XV alpha 1 chain

ENSG00000204305	AGER	advanced glycosylation end-product specific receptor
ENSG00000204308	RNF5	ring finger protein 5
ENSG00000204310	AGPAT1	1-acylglycerol-3-phosphate O-acyltransferase 1
ENSG00000204366	ZBTB12	zinc finger and BTB domain containing 12
ENSG00000204428	LY6G5C	lymphocyte antigen 6 family member G5C
ENSG00000204438	GPANK1	G-patch domain and ankyrin repeats 1
ENSG00000204469	PRRC2A	proline rich coiled-coil 2A
ENSG00000204472	AIF1	allograft inflammatory factor 1
ENSG00000204511	MCCD1	mitochondrial coiled-coil domain 1
ENSG00000204525	HLA-C	major histocompatibility complex, class I, C
ENSG00000204539	CDSN	corneodesmosin
ENSG00000204564	C6orf136	chromosome 6 open reading frame 136
ENSG00000204610	TRIM15	tripartite motif containing 15
ENSG00000204619	PPP1R11	protein phosphatase 1 regulatory inhibitor subunit 11
ENSG00000204628	RACK1	receptor for activated C kinase 1
ENSG00000204640	NMS	neuromedin S
ENSG00000204653	ASPDH	aspartate dehydrogenase domain containing
ENSG00000204701	OR2J3	olfactory receptor family 2 subfamily J member 3
ENSG00000204745		anaphase promoting complex subunit 1 (ANAPC1) pseudogene
ENSG00000204767	INSYN2B	inhibitory synaptic factor family member 2B
ENSG00000205302	SNX2	sorting nexin 2
ENSG00000205363	INSYN1	inhibitory synaptic factor 1
ENSG00000205420	KRT6A	keratin 6A
ENSG00000205423	CNEP1R1	CTD nuclear envelope phosphatase 1 regulatory subunit 1
ENSG00000205581	HMGN1	high mobility group nucleosome binding domain 1
ENSG00000205593	DENND6B	DENN domain containing 6B
ENSG00000205629	LCMT1	leucine carboxyl methyltransferase 1
ENSG00000205639	MFSD2B	major facilitator superfamily domain containing 2B
ENSG00000205643	CDPF1	cysteine rich DPF motif domain containing 1
ENSG00000205667	ARSH	arylsulfatase family member H
ENSG00000205795	CYS1	cystin 1
ENSG00000205940	HSP90AB2P	heat shock protein 90 alpha family class B member 2, pseudogene
ENSG00000205976		golgi autoantigen, golgin subfamily a, 6-like 2 (GOLGA6L2) pseudogene
ENSG00000206177	HBM	hemoglobin subunit mu
ENSG00000206203	TSSK2	testis specific serine kinase 2
ENSG00000206341	HLA-H	major histocompatibility complex, class I, H (pseudogene)
ENSG00000206474	OR10C1	olfactory receptor family 10 subfamily C member 1
ENSG00000206527	HACD2	3-hydroxyacyl-CoA dehydratase 2
ENSG00000206640		Y RNA [Source:RFAM;Acc:RF00019]
ENSG00000206650	SNORA70G	small nucleolar RNA, H/ACA box 70G
ENSG00000206889	RNU6-1200P	RNA, U6 small nuclear 1200, pseudogene
ENSG00000207025		Y RNA [Source:RFAM;Acc:RF00019]

ENSG00000207073		Y RNA [Source:RFAM;Acc:RF00019]
ENSG00000207108		Y RNA [Source:RFAM;Acc:RF00019]
ENSG00000207129	RNA5SP187	RNA, 5S ribosomal pseudogene 187
ENSG00000207201	RNU1-148P	RNA, U1 small nuclear 148, pseudogene
ENSG00000207237	RNU6-110P	RNA, U6 small nuclear 110, pseudogene
ENSG00000207247		Y RNA [Source:RFAM;Acc:RF00019]
ENSG00000207269	RN7SKP62	RN7SK pseudogene 62
ENSG00000207391		Y RNA [Source:RFAM;Acc:RF00019]
ENSG00000207393	RNU6-136P	RNA, U6 small nuclear 136, pseudogene
ENSG00000207438		Y RNA [Source:RFAM;Acc:RF00019]
ENSG00000207483	RNU6-1067P	RNA, U6 small nuclear 1067, pseudogene
ENSG00000207614	MIR193A	microRNA 193a
ENSG00000207649	MIR138-2	microRNA 138-2
ENSG00000207730	MIR200B	microRNA 200b
ENSG00000207752	MIR199A1	microRNA 199a-1
ENSG00000207782	MIR150	microRNA 150
ENSG00000210144	MT-TY	mitochondrially encoded tRNA-Tyr (UAU/C)
ENSG00000210164	MT-TG	mitochondrially encoded tRNA-Gly (GGN)
ENSG00000210176	MT-TH	mitochondrially encoded tRNA-His (CAU/C)
ENSG00000210195	MT-TT	mitochondrially encoded tRNA-Thr (ACN)
ENSG00000210196	MT-TP	mitochondrially encoded tRNA-Pro (CCN)
ENSG00000211445	GPX3	glutathione peroxidase 3
ENSG00000211734	TRBV5-1	T cell receptor beta variable 5-1
ENSG00000211752	TRBV27	T cell receptor beta variable 27
ENSG00000211791	TRAV13-2	T cell receptor alpha variable 13-2
ENSG00000211793	TRAV9-2	T cell receptor alpha variable 9-2
ENSG00000211810	TRAV29DV5	T cell receptor alpha variable 29/delta variable 5
ENSG00000211955	IGHV3-33	immunoglobulin heavy variable 3-33
ENSG00000211979	IGHV7-81	immunoglobulin heavy variable 7-81 (non-functional)
ENSG00000212123	PRR22	proline rich 22
ENSG00000212259	RNU6-308P	RNA, U6 small nuclear 308, pseudogene
ENSG00000212571	RNA5SP482	RNA, 5S ribosomal pseudogene 482
ENSG00000212695		ribosomal protein 18 (RPL18) pseudogene
ENSG00000212747	RTL8B	retrotransposon Gag like 8B
ENSG00000212789	ST13P5	ST13, Hsp70 interacting protein pseudogene 5
ENSG00000212939		novel transcript
ENSG00000213244	H3P4	H3 histone pseudogene 4
ENSG00000213316	LTC4S	leukotriene C4 synthase
ENSG00000213398	LCAT	lecithin-cholesterol acyltransferase
ENSG00000213406	ANXA2P1	annexin A2 pseudogene 1
ENSG00000213538	KRT8P41	keratin 8 pseudogene 41
ENSG00000213563	C8orf82	chromosome 8 open reading frame 82
ENSG00000213672	NCKIPSD	NCK interacting protein with SH3 domain

ENSG00000213676	ATF6B	activating transcription factor 6 beta
ENSG00000213937	CLDN9	claudin 9
ENSG00000214026	MRPL23	mitochondrial ribosomal protein L23
ENSG00000214160	ALG3	ALG3 alpha-1,3- mannosyltransferase
ENSG00000214309	MBLAC1	metallo-beta-lactamase domain containing 1
ENSG00000214510	SPINK13	serine peptidase inhibitor Kazal type 13
ENSG00000214517	PPME1	protein phosphatase methylesterase 1
ENSG00000214548	MEG3	maternally expressed 3
ENSG00000214655	ZSWIM8	zinc finger SWIM-type containing 8
ENSG00000214720	KRT18P49	keratin 18 pseudogene 49
ENSG00000215148	PRSS41	serine protease 41
ENSG00000215375	MYL5	myosin light chain 5
ENSG00000215458	AATBC	apoptosis associated transcript in bladder cancer
ENSG00000215474	SKOR2	SKI family transcriptional corepressor 2
ENSG00000215644	GCGR	glucagon receptor
ENSG00000215905		ribosomal protein L36 (RPL36) pseudogene
ENSG00000215910	C1orf167	chromosome 1 open reading frame 167
ENSG00000216425	PIMREGP2	PIMREG pseudogene 2
ENSG00000217644		ribosomal protein L18a (RPL18A) pseudogene
ENSG00000218690	H2AC10P	H2A clustered histone 10, pseudogene
ENSG00000218891	ZNF579	zinc finger protein 579
ENSG00000219410		novel transcript
ENSG00000220749	RPL21P28	ribosomal protein L21 pseudogene 28
ENSG00000220785	MTMR9LP	myotubularin related protein 9 like, pseudogene
ENSG00000221946	FXYD7	FXYD domain containing ion transport regulator 7
ENSG00000221968	FADS3	fatty acid desaturase 3
ENSG00000222150	RNA5SP239	RNA, 5S ribosomal pseudogene 239
ENSG00000222376	RN7SKP152	RN7SK pseudogene 152
ENSG00000222383	RNA5SP203	RNA, 5S ribosomal pseudogene 203
ENSG00000222428	RNA5SP231	RNA, 5S ribosomal pseudogene 231
ENSG00000222524	RN7SKP109	RN7SK pseudogene 109
ENSG00000222685	RN7SKP119	RN7SK pseudogene 119
ENSG00000222842	RN7SKP168	RN7SK pseudogene 168
ENSG00000222859	RN7SKP136	RN7SK pseudogene 136
ENSG00000222922	RNA5SP501	RNA, 5S ribosomal pseudogene 501
ENSG00000223040	RN7SKP144	RN7SK pseudogene 144
ENSG00000223118	RN7SKP102	RN7SK pseudogene 102
ENSG00000223128	RN7SKP140	RN7SK pseudogene 140
ENSG00000223361	FTH1P10	ferritin heavy chain 1 pseudogene 10
ENSG00000223374		novel transcript
ENSG00000223510	CDRT15	CMT1A duplicated region transcript 15
ENSG00000223511		novel transcript
ENSG00000223749	MIR503HG	MIR503 host gene

ENSG00000223776	LGALS8-AS1	LGALS8 antisense RNA 1
ENSG00000223825	DAZAP2P1	DAZ associated protein 2 pseudogene 1
ENSG00000224109	CENPVL3	centromere protein V like 3
ENSG00000224186	C5orf66	chromosome 5 open reading frame 66
ENSG00000224207		mannosidase, beta A, lysosomal-like (MANBAL) pseudogene
ENSG00000224294	PINCR	p53-induced noncoding RNA
ENSG00000224555		Non-histone chromosomal protein HMG-14 (HMGN1) pseudogene
ENSG00000224645		novel transcript
ENSG00000224958	PGM5-AS1	PGM5 antisense RNA 1
ENSG00000224971	SUMO2P3	SUMO2 pseudogene 3
ENSG00000225022	UBE2D3P1	ubiquitin conjugating enzyme E2 D3 pseudogene 1
ENSG00000225101	OR52K3P	olfactory receptor family 52 subfamily K member 3 pseudogene
ENSG00000225159	NPM1P39	nucleophosmin 1 pseudogene 39
ENSG00000225190	PLEKHM1	pleckstrin homology and RUN domain containing M1
ENSG00000225422	RBMS1P1	RNA binding motif single stranded interacting protein 1 pseudogene 1
ENSG00000225492	GBP1P1	guanylate binding protein 1 pseudogene 1
ENSG00000225530	SP3P	Sp3 transcription factor pseudogene
ENSG00000225573	RPL35P5	ribosomal protein L35 pseudogene 5
ENSG00000225770	RPS29P3	ribosomal protein S29 pseudogene 3
ENSG00000225774	SIRPAP1	signal regulatory protein alpha pseudogene 1
ENSG00000225855	RUSC1-AS1	RUSC1 antisense RNA 1
ENSG00000225973	PIGBOS1	PIGB opposite strand 1
ENSG00000226040		glycine cleavage system protein H (aminomethyl carrier) (GCSH) pseudogene
ENSG00000226131	PPIAP66	peptidylprolyl isomerase A pseudogene 66
ENSG00000226137	BAIAP2-DT	BAIAP2 divergent transcript
ENSG00000226138		ATPase, H+ transporting, lysosomal 14kDa, V1 subunit F (ATP6V1F) pseudogene
ENSG00000226415	TPI1P1	triosephosphate isomerase 1 pseudogene 1
ENSG00000226510	UPK1A-AS1	UPK1A antisense RNA 1
ENSG00000226547	SSU72P1	SSU72 pseudogene 1
ENSG00000226686	LINC01535	long intergenic non-protein coding RNA 1535
ENSG00000226800	CACTIN-AS1	CACTIN antisense RNA 1
ENSG00000226836		ribosomal protein L32 (RPL32) pseudogene
ENSG00000226970	NEDD8P1	NEDD8 pseudogene 1
ENSG00000226986	PRELID1P5	PRELID1 pseudogene 5
ENSG00000227205	PFN1P9	profilin 1 pseudogene 9
ENSG00000227311		ribosomal protein L21 (RPL21)
ENSG00000227437	RPS8P4	ribosomal protein S8 pseudogene 4
ENSG00000227500	SCAMP4	secretory carrier membrane protein 4
ENSG00000227525	RPL7P6	ribosomal protein L7 pseudogene 6
ENSG00000227747		ribosomal protein S2 (RPS2) pseudogene
ENSG00000227802	DNAJB3	DnaJ heat shock protein family (Hsp40) member B3
ENSG00000227946		novel transcript

ENSG00000228056	CFL1P3	cofilin 1 pseudogene 3
ENSG00000228172		novel transcript
ENSG00000228285	LYPLA2P1	LYPLA2 pseudogene 1
ENSG00000228286		docking protein 1, 62kDa (downstream of tyrosine kinase 1) (DOK1) pseudogene
ENSG00000228300	FAM174C	family with sequence similarity 174 member C
ENSG00000228312	GAPDHP45	glyceraldehyde 3 phosphate dehydrogenase pseudogene 45
ENSG00000228384		novel transcript
ENSG00000228409	CCT6P1	chaperonin containing TCP1 subunit 6 pseudogene 1
ENSG00000228436		novel transcript
ENSG00000228929	RPS13P2	ribosomal protein S13 pseudogene 2
ENSG00000228974		S100 calcium binding protein A11 (S100A11) pseudogene
ENSG00000228981		ribosomal protein S2 (RPS2) pseudogene
ENSG00000229048	DUTP1	deoxyuridine triphosphatase pseudogene 1
ENSG00000229107	ABHD17AP4	ABHD17A pseudogene 4
ENSG00000229190		novel transcript
ENSG00000229248	WBP2P1	WW domain binding protein 2 pseudogene 1
ENSG00000229596	MYL8P	myosin light chain 8, pseudogene
ENSG00000229809	ZNF688	zinc finger protein 688
ENSG00000229985		ribosomal protein L18a (RPL18A) pseudogene
ENSG00000230358	SPDYE21	speedy/RINGO cell cycle regulator family member E21
ENSG00000230626		novel protein similar to mitogen-activated protein kinase kinase 2 MAP2K2
ENSG00000230712	GGTLC4P	gamma-glutamyltransferase light chain 4 pseudogene
ENSG00000230979		ribosomal protein L18a (RPL18A) pseudogene
ENSG00000231010		novel transcript
ENSG00000231233	CFAP58-DT	CFAP58 divergent transcript
ENSG00000231313	CLIC1P1	chloride intracellular channel 1 pseudogene 1
ENSG00000231389	HLA-DPA1	major histocompatibility complex, class II, DP alpha 1
ENSG00000231643	YWHAQP8	YWHAQ pseudogene 8
ENSG00000231684	EIF1P3	eukaryotic translation initiation factor 1 pseudogene 3
ENSG00000231852	CYP21A2	cytochrome P450 family 21 subfamily A member 2
ENSG00000231909	MAP1LC3BP1	microtubule associated protein 1 light chain 3 beta pseudogene 1
ENSG00000231925	TAPBP	TAP binding protein
ENSG00000231991	ANXA2P2	annexin A2 pseudogene 2
ENSG00000232173	OR2H5P	olfactory receptor family 2 subfamily H member 5 pseudogene
ENSG00000232203	SLC25A6P2	solute carrier family 25 member 6 pseudogene 2
ENSG00000232374	GPR79	G protein-coupled receptor 79, pseudogene
ENSG00000232493	RPL12P11	ribosomal protein L12 pseudogene 11
ENSG00000232605	HMGN2P11	high mobility group nucleosomal binding domain 2 pseudogene 11
ENSG00000232818	RPS2P32	ribosomal protein S2 pseudogene 32
ENSG00000232832	LMLN-AS1	LMLN antisense RNA 1
ENSG00000233193		novel transcript
ENSG00000233237	LINC00472	long intergenic non-protein coding RNA 472

ENSG00000233259	FABP3P2	fatty acid binding protein 3 pseudogene 2
ENSG00000233360	PDXP-DT	PDXP divergent transcript
ENSG00000233426	EIF3FP3	eukaryotic translation initiation factor 3 subunit F pseudogene 3
ENSG00000233558		RPS5 (40S Ribosomal Protein S5) pseudogene
ENSG00000233762	RPS15P4	ribosomal protein S15 pseudogene 4
ENSG00000233838	DPH3P1	diphthamide biosynthesis 3 pseudogene 1
ENSG00000234072		novel transcript, antisense to GTF3C2 and EIF2B4
ENSG00000234118	RPL13AP6	ribosomal protein L13a pseudogene 6
ENSG00000234155	LINC02535	long intergenic non-protein coding RNA 2535
ENSG00000234432		uncharacterized LOC100129484 [Source:NCBI gene (formerly Entrezgene);Acc:100129484]
ENSG00000234568	BIN2P1	bridging integrator 2 pseudogene 1
ENSG00000234589		ribosomal protein L13a (RPL13A) pseudogene
ENSG00000234608	MAPKAPK5-AS1	MAPKAPK5 antisense RNA 1
ENSG00000234745	HLA-B	major histocompatibility complex, class I, B
ENSG00000234838	PPIAP78	peptidylprolyl isomerase A pseudogene 78
ENSG00000234925	ATP5PDP4	ATP synthase peripheral stalk subunit d pseudogene 4
ENSG00000235028	HMGN1P30	high mobility group nucleosome binding domain 1 pseudogene 30
ENSG00000235036		pseudogene similar to DNAJ protein
ENSG00000235110		ribosomal protein S17 (RPS17) pseudogene
ENSG00000235173	HGH1	HGH1 homolog
ENSG00000235226		ribosomal protein L27 (RPL27) pseudogene
ENSG00000235244	DANT2	DXZ4 associated non-coding transcript 2, distal
ENSG00000235282	DYNLL1P3	dynein light chain LC8-type 1 pseudogene 3
ENSG00000235444	PSMB3P2	proteasome subunit beta 3 pseudogene 2
ENSG00000235501		novel transcript
ENSG00000235508	RPS2P7	ribosomal protein S2 pseudogene 7
ENSG00000235513	L3MBTL2-AS1	L3MBTL2 antisense RNA 1
ENSG00000235568	NFAM1	NFAT activating protein with ITAM motif 1
ENSG00000235581		ribosomal protein S28 (RPS28) pseudogene
ENSG00000235602	POU5F1P3	POU class 5 homeobox 1 pseudogene 3
ENSG00000235605		ribosomal protein S15 (RPS15) pseudogene
ENSG00000235649	MXRA5Y	matrix remodeling associated 5 Y-linked (pseudogene)
ENSG00000235710	TRIM67-AS1	TRIM67 antisense RNA 1
ENSG00000235718	MFRP	membrane frizzled-related protein
ENSG00000235821	IFITM4P	interferon induced transmembrane protein 4 pseudogene
ENSG00000235862		novel transcript
ENSG00000235865	GSN-AS1	GSN antisense RNA 1
ENSG00000236148	RPL23AP37	ribosomal protein L23a pseudogene 37
ENSG00000236216	PPP1R11P1	protein phosphatase 1 regulatory inhibitor subunit 11 pseudogene 1
ENSG00000236281	NDUFB9P2	NADH:ubiquinone oxidoreductase subunit B9 pseudogene 2
ENSG00000236333	TRHDE-AS1	TRHDE antisense RNA 1
ENSG00000236384	LINC00479	long intergenic non-protein coding RNA 479

ENSG00000236496	GPS2P1	G protein pathway suppressor 2 pseudogene 1
ENSG00000236499	LINC00896	long intergenic non-protein coding RNA 896
ENSG00000236616	BAK1P2	BCL2 antagonist/killer 1 pseudogene 2
ENSG00000236683	HMGA1P1	high mobility group AT-hook 1 pseudogene 1
ENSG00000236773		novel pseudogene
ENSG00000236852	SSBL2P	SSB like 2, pseudogene
ENSG00000237004	ZNRF2P1	zinc and ring finger 2 pseudogene 1
ENSG00000237424	FOXD2-AS1	FOXD2 adjacent opposite strand RNA 1
ENSG00000237687	LINC00686	long intergenic non-protein coding RNA 686
ENSG00000237988	OR2I1P	olfactory receptor family 2 subfamily I member 1 pseudogene
ENSG00000238003	RPL10P4	ribosomal protein L10 pseudogene 4
ENSG00000238168	IFITM3P5	IFITM3 pseudogene 5
ENSG00000238172	RPS2P35	ribosomal protein S2 pseudogene 35
ENSG00000238222	MKRN4P	makorin ring finger protein 4, pseudogene
ENSG00000238227	TMEM250	transmembrane protein 250
ENSG00000238244	GABARAPL3	GABA type A receptor associated protein like 3 pseudogene
ENSG00000238926		Y RNA [Source:RFAM;Acc:RF00019]
ENSG00000239035	SNORD13D	small nucleolar RNA, C/D box 13D
ENSG00000239211	RN7SL563P	RNA, 7SL, cytoplasmic 563, pseudogene
ENSG00000239228	RN7SL578P	RNA, 7SL, cytoplasmic 578, pseudogene
ENSG00000239250	RN7SL271P	RNA, 7SL, cytoplasmic 271, pseudogene
ENSG00000239356	RN7SL309P	RNA, 7SL, cytoplasmic 309, pseudogene
ENSG00000239445	ST3GAL6-AS1	ST3GAL6 antisense RNA 1
ENSG00000239470		ribosomal protein L21 (RPL21) pseudogene
ENSG00000239545	RN7SL822P	RNA, 7SL, cytoplasmic 822, pseudogene
ENSG00000239649	MYADML	myeloid associated differentiation marker like (pseudogene)
ENSG00000239742	RN7SL672P	RNA, 7SL, cytoplasmic 672, pseudogene
ENSG00000239744	RN7SL63P	RNA, 7SL, cytoplasmic 63, pseudogene
ENSG00000239883	PARGP1	poly(ADP-ribose) glycohydrolase pseudogene 1
ENSG00000239958	RN7SL51P	RNA, 7SL, cytoplasmic 51, pseudogene
ENSG00000239961	LILRA4	leukocyte immunoglobulin like receptor A4
ENSG00000240374	RN7SL503P	RNA, 7SL, cytoplasmic 503, pseudogene
ENSG00000240577	RN7SL445P	RNA, 7SL, cytoplasmic 445, pseudogene
ENSG00000240602	AADACP1	arylacetamide deacetylase pseudogene 1
ENSG00000240723	RN7SL382P	RNA, 7SL, cytoplasmic 382, pseudogene
ENSG00000240837	RN7SL77P	RNA, 7SL, cytoplasmic 77, pseudogene
ENSG00000240970	RPL23AP64	ribosomal protein L23a pseudogene 64
ENSG00000241174	RN7SL570P	RNA, 7SL, cytoplasmic 570, pseudogene
ENSG00000241207		psiSSX8 pseudogene (psiSSX8)
ENSG00000241553	ARPC4	actin related protein 2/3 complex subunit 4
ENSG00000241604	RN7SL340P	RNA, 7SL, cytoplasmic 340, pseudogene
ENSG00000241627	UBQLN4P1	ubiquilin 4 pseudogene 1
ENSG00000241651		ribosomal protein L15 (RPL15) pseudogene

ENSG00000241789	RN7SL504P	RNA, 7SL, cytoplasmic 504, pseudogene
ENSG00000242065	RN7SL291P	RNA, 7SL, cytoplasmic 291, pseudogene
ENSG00000242165	RN7SL222P	RNA, 7SL, cytoplasmic 222, pseudogene
ENSG00000242241	RN7SL306P	RNA, 7SL, cytoplasmic 306, pseudogene
ENSG00000242509	RN7SL156P	RNA, 7SL, cytoplasmic 156, pseudogene
ENSG00000242599	CSAG4	CSAG family member 4 (pseudogene)
ENSG00000242638	RN7SL294P	RNA, 7SL, cytoplasmic 294, pseudogene
ENSG00000242651	RN7SL862P	RNA, 7SL, cytoplasmic 862, pseudogene
ENSG00000242675	RPS16P9	ribosomal protein S16 pseudogene 9
ENSG00000242696	RN7SL40P	RNA, 7SL, cytoplasmic 40, pseudogene
ENSG00000242732	RTL5	retrotransposon Gag like 5
ENSG00000242752	NMTRQ-TTG12-1	nuclear-encoded mitochondrial tRNA-Gln (TTG) 12-1
ENSG00000242802	AP5Z1	adaptor related protein complex 5 subunit zeta 1
ENSG00000242992	FTH1P4	ferritin heavy chain 1 pseudogene 4
ENSG00000243048	FTHL18	ferritin heavy chain like 18
ENSG00000243136	RN7SL22P	RNA, 7SL, cytoplasmic 22, pseudogene
ENSG00000243365	RN7SL278P	RNA, 7SL, cytoplasmic 278, pseudogene
ENSG00000243439	RN7SL352P	RNA, 7SL, cytoplasmic 352, pseudogene
ENSG00000243541	RN7SL104P	RNA, 7SL, cytoplasmic 104, pseudogene
ENSG00000243633	RN7SL542P	RNA, 7SL, cytoplasmic 542, pseudogene
ENSG00000243856	RN7SL551P	RNA, 7SL, cytoplasmic 551, pseudogene
ENSG00000244057	LCE3C	late cornified envelope 3C
ENSG00000244104	RN7SL659P	RNA, 7SL, cytoplasmic 659, pseudogene
ENSG00000244171	PBX2P1	PBX homeobox 2 pseudogene 1
ENSG00000244337		novel transcript
ENSG00000244381	SDHDP3	succinate dehydrogenase complex subunit D pseudogene 3
ENSG00000244425	RN7SL268P	RNA, 7SL, cytoplasmic 268, pseudogene
ENSG00000244476	ERVFRD-1	endogenous retrovirus group FRD member 1, envelope
ENSG00000244486	SCARF2	scavenger receptor class F member 2
ENSG00000244532	RN7SL380P	RNA, 7SL, cytoplasmic 380, pseudogene
ENSG00000244537	KRTAP4-2	keratin associated protein 4-2
ENSG00000244694	PTCHD4	patched domain containing 4
ENSG00000245498		novel transcript, antisense to C11orf61
ENSG00000245571	FAM111A-DT	FAM111A divergent transcript
ENSG00000247315	ZCCHC3	zinc finger CCHC-type containing 3
ENSG00000247596	TWF2	twinfilin actin binding protein 2
ENSG00000248429	FAM198B-AS1	FAM198B antisense RNA 1
ENSG00000248485	PCP4L1	Purkinje cell protein 4 like 1
ENSG00000248578	NPM1P21	nucleophosmin 1 pseudogene 21
ENSG00000249936	RAC1P2	Rac family small GTPase 1 pseudogene 2
ENSG00000249992	TMEM158	transmembrane protein 158
ENSG00000250271		arylacetamide deacetylase (esterase) (AADAC) pseudogene
ENSG00000250479	CHCHD10	coiled-coil-helix-coiled-coil-helix domain containing 10

ENSG00000250510	GPR162	G protein-coupled receptor 162
ENSG00000250571	GLI4	GLI family zinc finger 4
ENSG00000251017		coiled-coil domain containing 90B (CCDC90B) pseudogene
ENSG00000251297	TUBB7P	tubulin beta 7 pseudogene
ENSG00000251333	RTN3P1	reticulon 3 pseudogene 1
ENSG00000251785	RNA5SP20	RNA, 5S ribosomal pseudogene 20
ENSG00000251837		Y RNA [Source:RFAM;Acc:RF00019]
ENSG00000251924	RNA5SP408	RNA, 5S ribosomal pseudogene 408
ENSG00000251941	RNA5SP116	RNA, 5S ribosomal pseudogene 116
ENSG00000252041	RNA5SP228	RNA, 5S ribosomal pseudogene 228
ENSG00000252064		Y RNA [Source:RFAM;Acc:RF00019]
ENSG00000252289	RNA5SP519	RNA, 5S ribosomal pseudogene 519
ENSG00000252336	RNA5SP148	RNA, 5S ribosomal pseudogene 148
ENSG00000252420		Y RNA [Source:RFAM;Acc:RF00019]
ENSG00000252424	RNA5SP384	RNA, 5S ribosomal pseudogene 384
ENSG00000252509	RNA5SP271	RNA, 5S ribosomal pseudogene 271
ENSG00000252526		Small nucleolar RNA SNORA70 [Source:RFAM;Acc:RF00156]
ENSG00000252660		Y RNA [Source:RFAM;Acc:RF00019]
ENSG00000252729	RNU6-971P	RNA, U6 small nuclear 971, pseudogene
ENSG00000252764	RNU6-1092P	RNA, U6 small nuclear 1092, pseudogene
ENSG00000252898	RNU6-1096P	RNA, U6 small nuclear 1096, pseudogene
ENSG00000252996	RNU6-1315P	RNA, U6 small nuclear 1315, pseudogene
ENSG00000253570	RNF5P1	ring finger protein 5 pseudogene 1
ENSG00000253738	OTUD6B-AS1	OTUD6B antisense RNA 1 (head to head)
ENSG00000253954	HMGN1P38	high mobility group nucleosome binding domain 1 pseudogene 38
ENSG00000253958	CLDN23	claudin 23
ENSG00000254402	LRRC24	leucine rich repeat containing 24
ENSG00000254618	TMED10P1	transmembrane p24 trafficking protein 10 pseudogene 1
ENSG00000254835	RNF185-AS1	RNF185 antisense RNA 1
ENSG00000254858	MPV17L2	MPV17 mitochondrial inner membrane protein like 2
ENSG00000254986	DPP3	dipeptidyl peptidase 3
ENSG00000254999	BRK1	BRICK1 subunit of SCAR/WAVE actin nucleating complex
ENSG00000255138	GLTPP1	glycolipid transfer protein pseudogene 1
ENSG00000255262	ELOBP2	elongin B pseudogene 2
ENSG00000255282	WTAPP1	Wilms tumor 1 associated protein pseudogene 1
ENSG00000255328		novel transcript
ENSG00000255363	LINC02757	long intergenic non-protein coding RNA 2757
ENSG00000255422		novel transcript
ENSG00000255629		novel transcript
ENSG00000255690	TRIL	TLR4 interactor with leucine rich repeats
ENSG00000255769	GOLGA2P10	GOLGA2 pseudogene 10
ENSG00000255815	KRT8P11	keratin 8 pseudogene 11
ENSG00000256103	ATP5MFP5	ATP synthase membrane subunit f pseudogene 5

ENSG00000256268	LINC02454	long intergenic non-protein coding RNA 2454
ENSG00000256463	SALL3	spalt like transcription factor 3
ENSG00000256713	PGA5	pepsinogen A5
ENSG00000257086		novel transcript
ENSG00000258071	ARL2BPP2	ADP ribosylation factor like GTPase 2 binding protein pseudogene 2
ENSG00000258738		novel transcript, antisense to BAZ1A
ENSG00000258897	EGLN3-AS1	EGLN3 antisense RNA 1
ENSG00000258986	TMEM179	transmembrane protein 179
ENSG00000259020		prothymosin, alpha (PTMA) pseudogene
ENSG00000259032	ENSAP2	endosulfine alpha pseudogene 2
ENSG00000259107	LINC00911	long intergenic non-protein coding RNA 911
ENSG00000259498	TPM1-AS	TPM1 antisense RNA
ENSG00000259557	HMGN1P26	high mobility group nucleosome binding domain 1 pseudogene 26
ENSG00000259607		novel transcript, antisense to KIF13B
ENSG00000259712		novel transcript, antisense to MAPK6
ENSG00000259838	ELOCP2	elongin C pseudogene 2
ENSG00000260082		chromosome 20 open reading frame 27 (C20orf27p) pseudogene
ENSG00000260121		novel transcript, antisense to FAM38A
ENSG00000260233	ZNRD2-AS1	ZNRD2 antisense RNA 1 (head to head)
ENSG00000260279		novel transcript, antisense to ANKRD11
ENSG00000260496		novel transcript
ENSG00000261192	RNF126P1	ring finger protein 126 pseudogene 1
ENSG00000261221	ZNF865	zinc finger protein 865
ENSG00000261282	SOD1P2	superoxide dismutase 1 pseudogene 2
ENSG00000261678	SCRT1	scratch family transcriptional repressor 1
ENSG00000261787	TCF24	transcription factor 24
ENSG00000261924		novel transcript, antisense to RPTOR
ENSG00000263426	RN7SL471P	RNA, 7SL, cytoplasmic 471, pseudogene
ENSG00000263479	RN7SL509P	RNA, 7SL, cytoplasmic 509, pseudogene
ENSG00000263905	RN7SL555P	RNA, 7SL, cytoplasmic 555, pseudogene
ENSG00000263988	RN7SL147P	RNA, 7SL, cytoplasmic 147, pseudogene
ENSG00000264215		novel transcript
ENSG00000264226	MIR3168	microRNA 3168
ENSG00000265243	IGLJCOR18	immunoglobulin lambda joining-constant/OR18 (pseudogene)
ENSG00000265813	RN7SL300P	RNA, 7SL, cytoplasmic 300, pseudogene
ENSG00000265892	RN7SL311P	RNA, 7SL, cytoplasmic 311, pseudogene
ENSG00000265894	RN7SL357P	RNA, 7SL, cytoplasmic 357, pseudogene
ENSG00000265972	TXNIP	thioredoxin interacting protein
ENSG00000266059	RN7SL140P	RNA, 7SL, cytoplasmic 140, pseudogene
ENSG00000266074	BAHCC1	BAH domain and coiled-coil containing 1
ENSG00000266274	RN7SL138P	RNA, 7SL, cytoplasmic 138, pseudogene
ENSG00000266302		novel transcript
ENSG00000266433	TBC1D3P5	TBC1 domain family member 3 pseudogene 5

ENSG00000266524	GDF10	growth differentiation factor 10
ENSG00000266751	MIR3661	microRNA 3661
ENSG00000266794	RN7SL7P	RNA, 7SL, cytoplasmic 7, pseudogene
ENSG00000266877		novel transcript
ENSG00000266980		novel transcript, antisense to UNK
ENSG00000267156	TPMTP1	thiopurine S-methyltransferase pseudogene 1
ENSG00000267200	MIR132	microRNA 132
ENSG00000267248		novel transcript
ENSG00000267258	PPIAP58	peptidylprolyl isomerase A pseudogene 58
ENSG00000267384	SMCO4P1	single-pass membrane protein with coiled-coil domains 4 pseudogene 1
ENSG00000267472	ARHGAP27P2	Rho GTPase activating protein 27 pseudogene 2
ENSG00000267629		novel transcript, readthrough between TM6SF2 and HAPLN4
ENSG00000267710	EDDM13	epididymal protein 13
ENSG00000268297	CLEC4GP1	C-type lectin domain family 4 member G pseudogene 1
ENSG00000268350	FAM156A	family with sequence similarity 156 member A
ENSG00000268509		novel transcript, antisense to EDEM1
ENSG00000268635		novel transcript, antisense to PAK1
ENSG00000268758	ADGRE4P	adhesion G protein-coupled receptor E4, pseudogene
ENSG00000268799	H3Y2	H3.Y histone 2
ENSG00000268983		novel transcript, antisense to KXD1
ENSG00000270194		novel transcript, antisense to GOLGA4
ENSG00000270607		novel transcript
ENSG00000270980		coiled-coil-helix-coiled-coil-helix domain containing 2 (CHCHD2) pseudogene
ENSG00000271360		novel transcript, antisense to USP6NL
ENSG00000271605	MILR1	mast cell immunoglobulin like receptor 1
ENSG00000271707	ATP1B3P1	ATPase Na+/K+ transporting subunit beta 3 pseudogene 1
ENSG00000271806		novel transcript, antisense PRKCZ
ENSG00000272031	ANKRD34A	ankyrin repeat domain 34A
ENSG00000272051		Y RNA [Source:RFAM;Acc:RF00019]
ENSG00000272075	RN7SL828P	RNA, 7SL, cytoplasmic 828, pseudogene
ENSG00000272160	RNU4-5P	RNA, U4 small nuclear 5, pseudogene
ENSG00000272333	KMT2B	lysine methyltransferase 2B
ENSG00000272391	POM121C	POM121 transmembrane nucleoporin C
ENSG00000272841	MAP3K4-AS1	MAP3K4 antisense RNA 1
ENSG00000272886	DCP1A	decapping mRNA 1A
ENSG00000273703	H2BC14	H2B clustered histone 14
ENSG00000273749	CYFIP1	cytoplasmic FMR1 interacting protein 1
ENSG00000274290	H2BC6	H2B clustered histone 6
ENSG00000274523	RCC1L	RCC1 like
ENSG00000274727		IQ motif and Sec7 domain 3 (IQSEC3) pseudogene
ENSG00000274756		ribosomal protein S2 (RPS2) pseudogene
ENSG00000274808	TBC1D3B	TBC1 domain family member 3B

ENSG00000275023	MLLT6	MLLT6, PHD finger containing
ENSG00000275074	NUDT18	nudix hydrolase 18
ENSG00000275139		novel transcript, antisense to PCBP3
ENSG00000275342	PRAG1	PEAK1 related, kinase-activating pseudokinase 1
ENSG00000275663	H4C7	H4 clustered histone 7
ENSG00000275713	H2BC9	H2B clustered histone 9
ENSG00000275878	RN7SL43P	RNA, 7SL, cytoplasmic 43, pseudogene
ENSG00000275880		novel transcript, antisense to CARKD
ENSG00000276014	RN7SL301P	RNA, 7SL, cytoplasmic 301, pseudogene
ENSG00000276047	RN7SL711P	RNA, 7SL, cytoplasmic 711, pseudogene
ENSG00000276291	FRG1HP	FSHD region gene 1 family member H, pseudogene
ENSG00000276600	RAB7B	RAB7B, member RAS oncogene family
ENSG00000276805		general transcription factor IIi (GTF2I) pseudogene
ENSG00000277224	H2BC7	H2B clustered histone 7
ENSG00000277265	RN7SL556P	RNA, 7SL, cytoplasmic 556, pseudogene
ENSG00000277586	NEFL	neurofilament light
ENSG00000277591	RN7SL575P	RNA, 7SL, cytoplasmic 575, pseudogene
ENSG00000277654		glioma tumor suppressor candidate region gene 2 (GLTSCR2) pseudogene
ENSG00000277775	H3C7	H3 clustered histone 7
ENSG00000278238		novel transcript
ENSG00000278523		Y RNA [Source:RFAM;Acc:RF00019]
ENSG00000278535	DHRS11	dehydrogenase/reductase 11
ENSG00000278611	ZNF426-DT	ZNF426 divergent transcript
ENSG00000279072		novel piRNA host transcript
ENSG00000279081		eukaryotic translation initiation factor 3 subunit F (EIF3F) pseudogene
ENSG00000279152		TEC
ENSG00000279155		TEC
ENSG00000279255		TEC
ENSG00000279392		ubiquitin-conjugating enzyme E2L 3 (UBE2L3) pseudogene
ENSG00000279484	KLHL30-AS1	KLHL30 antisense RNA 1
ENSG00000279523		novel transcript
ENSG00000279610	C20orf181	chromosome 20 open reading frame 181
ENSG00000279694		TEC
ENSG00000279725		TEC
ENSG00000279730	KMT5AP1	KMT5A pseudogene 1
ENSG00000280266		TEC
ENSG00000280409	LINC01101	long intergenic non-protein coding RNA 1101
ENSG00000280414		novel transcript
ENSG00000280486		novel transcript, antisense to NDUFS7
ENSG00000281005	LINC00921	long intergenic non-protein coding RNA 921
ENSG00000281832	LINC00602	long intergenic non-protein coding RNA 602
ENSG00000283297	TEX52	testis expressed 52

ENSG00000283540	MIR520F	microRNA 520f
ENSG00000283787	PRR33	proline rich 33
ENSG00000283907		novel transcript, antisense to ATP4A
ENSG00000284395	PERCC1	proline and glutamate rich with coiled coil 1
ENSG00000284638		novel protein
ENSG00000286975		novel transcript
ENSG00000287263		novel transcript
ENSG00000287276		contactin associated protein like 3B (CNTNAP3B) pseudogene
ENSG00000287415		novel transcript
ENSG00000287706		novel transcript
**Down-Regulated Genes ↓ (1579)**		
ENSG00000000460	C1orf112	chromosome 1 open reading frame 112
ENSG00000001167	NFYA	nuclear transcription factor Y subunit alpha
ENSG00000003393	ALS2	alsin Rho guanine nucleotide exchange factor ALS2
ENSG00000003509	NDUFAF7	NADH:ubiquinone oxidoreductase complex assembly factor 7
ENSG00000004455	AK2	adenylate kinase 2
ENSG00000004700	RECQL	RecQ like helicase
ENSG00000004766	VPS50	VPS50 subunit of EARP/GARPII complex
ENSG00000004864	SLC25A13	solute carrier family 25 member 13
ENSG00000004897	CDC27	cell division cycle 27
ENSG00000005469	CROT	carnitine O-octanoyltransferase
ENSG00000005810	MYCBP2	MYC binding protein 2
ENSG00000005812	FBXL3	F-box and leucine rich repeat protein 3
ENSG00000006576	PHTF2	putative homeodomain transcription factor 2
ENSG00000006715	VPS41	VPS41 subunit of HOPS complex
ENSG00000007168	PAFAH1B1	platelet activating factor acetylhydrolase 1b regulatory subunit 1
ENSG00000007202	KIAA0100	KIAA0100
ENSG00000007545	CRAMP1	cramped chromatin regulator homolog 1
ENSG00000008277	ADAM22	ADAM metallopeptidase domain 22
ENSG00000009335	UBE3C	ubiquitin protein ligase E3C
ENSG00000010072	SPRTN	SprT-like N-terminal domain
ENSG00000010292	NCAPD2	non-SMC condensin I complex subunit D2
ENSG00000011295	TTC19	tetratricopeptide repeat domain 19
ENSG00000011454	RABGAP1	RAB GTPase activating protein 1
ENSG00000012048	BRCA1	BRCA1 DNA repair associated
ENSG00000012983	MAP4K5	mitogen-activated protein kinase kinase kinase kinase 5
ENSG00000013503	POLR3B	RNA polymerase III subunit B
ENSG00000014824	SLC30A9	solute carrier family 30 member 9
ENSG00000015153	YAF2	YY1 associated factor 2
ENSG00000015171	ZMYND11	zinc finger MYND-type containing 11
ENSG00000017260	ATP2C1	ATPase secretory pathway Ca2+ transporting 1
ENSG00000018699	TTC27	tetratricopeptide repeat domain 27
ENSG00000019995	ZRANB1	zinc finger RANBP2-type containing 1

ENSG00000020426	MNAT1	MNAT1 component of CDK activating kinase
ENSG00000020922	MRE11	MRE11 homolog, double strand break repair nuclease
ENSG00000021488	SLC7A9	solute carrier family 7 member 9
ENSG00000023839	ABCC2	ATP binding cassette subfamily C member 2
ENSG00000023892	DEF6	DEF6 guanine nucleotide exchange factor
ENSG00000026652	AGPAT4	1-acylglycerol-3-phosphate O-acyltransferase 4
ENSG00000029153	ARNTL2	aryl hydrocarbon receptor nuclear translocator like 2
ENSG00000030419	IKZF2	IKAROS family zinc finger 2
ENSG00000032742	IFT88	intraflagellar transport 88
ENSG00000033170	FUT8	fucosyltransferase 8
ENSG00000033178	UBA6	ubiquitin like modifier activating enzyme 6
ENSG00000034677	RNF19A	ring finger protein 19A, RBR E3 ubiquitin protein ligase
ENSG00000035687	ADSS2	adenylosuccinate synthase 2
ENSG00000036257	CUL3	cullin 3
ENSG00000040341	STAU2	staufen double-stranded RNA binding protein 2
ENSG00000043514	TRIT1	tRNA isopentenyltransferase 1
ENSG00000046651	OFD1	OFD1 centriole and centriolar satellite protein
ENSG00000047188	YTHDC2	YTH domain containing 2
ENSG00000047230	CTPS2	CTP synthase 2
ENSG00000047249	ATP6V1H	ATPase H+ transporting V1 subunit H
ENSG00000047365	ARAP2	ArfGAP with RhoGAP domain, ankyrin repeat and PH domain 2
ENSG00000047579	DTNBP1	dystrobrevin binding protein 1
ENSG00000047621	C12orf4	chromosome 12 open reading frame 4
ENSG00000048544	MRPS10	mitochondrial ribosomal protein S10
ENSG00000048649	RSF1	remodeling and spacing factor 1
ENSG00000048707	VPS13D	vacuolar protein sorting 13 homolog D
ENSG00000049167	ERCC8	ERCC excision repair 8, CSA ubiquitin ligase complex subunit
ENSG00000049192	ADAMTS6	ADAM metallopeptidase with thrombospondin type 1 motif 6
ENSG00000050438	SLC4A8	solute carrier family 4 member 8
ENSG00000050730	TNIP3	TNFAIP3 interacting protein 3
ENSG00000051341	POLQ	DNA polymerase theta
ENSG00000051382	PIK3CB	phosphatidylinositol-4,5-bisphosphate 3-kinase catalytic subunit beta
ENSG00000051825	MPHOSPH9	M-phase phosphoprotein 9
ENSG00000053524	MCF2L2	MCF.2 cell line derived transforming sequence-like 2
ENSG00000054118	THRAP3	thyroid hormone receptor associated protein 3
ENSG00000054611	TBC1D22A	TBC1 domain family member 22A
ENSG00000056558	TRAF1	TNF receptor associated factor 1
ENSG00000056972	TRAF3IP2	TRAF3 interacting protein 2
ENSG00000058056	USP13	ubiquitin specific peptidase 13
ENSG00000058091	CDK14	cyclin dependent kinase 14
ENSG00000058804	NDC1	NDC1 transmembrane nucleoporin
ENSG00000060237	WNK1	WNK lysine deficient protein kinase 1
ENSG00000060982	BCAT1	branched chain amino acid transaminase 1

ENSG00000061987	MON2	MON2 homolog, regulator of endosome-to-Golgi trafficking
ENSG00000063601	MTMR1	myotubularin related protein 1
ENSG00000064313	TAF2	TATA-box binding protein associated factor 2
ENSG00000064419	TNPO3	transportin 3
ENSG00000064933	PMS1	PMS1 homolog 1, mismatch repair system component
ENSG00000065135	GNAI3	G protein subunit alpha i3
ENSG00000065613	SLK	STE20 like kinase
ENSG00000065802	ASB1	ankyrin repeat and SOCS box containing 1
ENSG00000066651	TRMT11	tRNA methyltransferase 11 homolog
ENSG00000066697	MSANTD3	Myb/SANT DNA binding domain containing 3
ENSG00000066739	ATG2B	autophagy related 2B
ENSG00000066827	ZFAT	zinc finger and AT-hook domain containing
ENSG00000067369	TP53BP1	tumor protein p53 binding protein 1
ENSG00000067900	ROCK1	Rho associated coiled-coil containing protein kinase 1
ENSG00000067955	CBFB	core-binding factor subunit beta
ENSG00000067992	PDK3	pyruvate dehydrogenase kinase 3
ENSG00000068878	PSME4	proteasome activator subunit 4
ENSG00000068976	PYGM	glycogen phosphorylase, muscle associated
ENSG00000069345	DNAJA2	DnaJ heat shock protein family (Hsp40) member A2
ENSG00000069667	RORA	RAR related orphan receptor A
ENSG00000069966	GNB5	G protein subunit beta 5
ENSG00000069974	RAB27A	RAB27A, member RAS oncogene family
ENSG00000070081	NUCB2	nucleobindin 2
ENSG00000070526	ST6GALNAC1	ST6 N-acetylgalactosaminide alpha-2,6-sialyltransferase 1
ENSG00000070831	CDC42	cell division cycle 42
ENSG00000070950	RAD18	RAD18 E3 ubiquitin protein ligase
ENSG00000071243	ING3	inhibitor of growth family member 3
ENSG00000071539	TRIP13	thyroid hormone receptor interactor 13
ENSG00000071575	TRIB2	tribbles pseudokinase 2
ENSG00000072364	AFF4	AF4/FMR2 family member 4
ENSG00000072571	HMMR	hyaluronan mediated motility receptor
ENSG00000072694	FCGR2B	Fc fragment of IgG receptor IIb
ENSG00000072858	SIDT1	SID1 transmembrane family member 1
ENSG00000073756	PTGS2	prostaglandin-endoperoxide synthase 2
ENSG00000074054	CLASP1	cytoplasmic linker associated protein 1
ENSG00000075213	SEMA3A	semaphorin 3A
ENSG00000075292	ZNF638	zinc finger protein 638
ENSG00000075413	MARK3	microtubule affinity regulating kinase 3
ENSG00000075539	FRYL	FRY like transcription coactivator
ENSG00000075618	FSCN1	fascin actin-bundling protein 1
ENSG00000075711	DLG1	discs large MAGUK scaffold protein 1
ENSG00000075884	ARHGAP15	Rho GTPase activating protein 15
ENSG00000075914	EXOSC7	exosome component 7

ENSG00000075945	KIFAP3	kinesin associated protein 3
ENSG00000075975	MKRN2	makorin ring finger protein 2
ENSG00000076003	MCM6	minichromosome maintenance complex component 6
ENSG00000077232	DNAJC10	DnaJ heat shock protein family (Hsp40) member C10
ENSG00000077380	DYNC1I2	dynein cytoplasmic 1 intermediate chain 2
ENSG00000078043	PIAS2	protein inhibitor of activated STAT 2
ENSG00000078177	N4BP2	NEDD4 binding protein 2
ENSG00000078269	SYNJ2	synaptojanin 2
ENSG00000078618	NRDC	nardilysin convertase
ENSG00000078674	PCM1	pericentriolar material 1
ENSG00000078747	ITCH	itchy E3 ubiquitin protein ligase
ENSG00000078967	UBE2D4	ubiquitin conjugating enzyme E2 D4 (putative)
ENSG00000079257	LXN	latexin
ENSG00000079263	SP140	SP140 nuclear body protein
ENSG00000079739	PGM1	phosphoglucomutase 1
ENSG00000080166	DCT	dopachrome tautomerase
ENSG00000080298	RFX3	regulatory factor X3
ENSG00000080802	CNOT4	CCR4-NOT transcription complex subunit 4
ENSG00000080839	RBL1	RB transcriptional corepressor like 1
ENSG00000080986	NDC80	NDC80 kinetochore complex component
ENSG00000081014	AP4E1	adaptor related protein complex 4 subunit epsilon 1
ENSG00000081019	RSBN1	round spermatid basic protein 1
ENSG00000081148	IMPG2	interphotoreceptor matrix proteoglycan 2
ENSG00000083097	DOP1A	DOP1 leucine zipper like protein A
ENSG00000083123	BCKDHB	branched chain keto acid dehydrogenase E1 subunit beta
ENSG00000083312	TNPO1	transportin 1
ENSG00000083520	DIS3	DIS3 homolog, exosome endoribonuclease and 3'-5' exoribonuclease
ENSG00000083535	PIBF1	progesterone immunomodulatory binding factor 1
ENSG00000083544	TDRD3	tudor domain containing 3
ENSG00000083642	PDS5B	PDS5 cohesin associated factor B
ENSG00000083750	RRAGB	Ras related GTP binding B
ENSG00000083828	ZNF586	zinc finger protein 586
ENSG00000084112	SSH1	slingshot protein phosphatase 1
ENSG00000084734	GCKR	glucokinase regulator
ENSG00000085117	CD82	CD82 molecule
ENSG00000085224	ATRX	ATRX chromatin remodeler
ENSG00000085511	MAP3K4	mitogen-activated protein kinase kinase kinase 4
ENSG00000085563	ABCB1	ATP binding cassette subfamily B member 1
ENSG00000086666	ZFAND6	zinc finger AN1-type containing 6
ENSG00000087053	MTMR2	myotubularin related protein 2
ENSG00000088035	ALG6	ALG6 alpha-1,3-glucosyltransferase
ENSG00000088970	KIZ	kizuna centrosomal protein
ENSG00000089091	DZANK1	double zinc ribbon and ankyrin repeat domains 1

ENSG00000089123	TASP1	taspase 1
ENSG00000089177	KIF16B	kinesin family member 16B
ENSG00000091140	DLD	dihydrolipoamide dehydrogenase
ENSG00000091157	WDR7	WD repeat domain 7
ENSG00000091409	ITGA6	integrin subunit alpha 6
ENSG00000091436	MAP3K20	mitogen-activated protein kinase kinase kinase 20
ENSG00000091482	SMPX	small muscle protein X-linked
ENSG00000092148	HECTD1	HECT domain E3 ubiquitin protein ligase 1
ENSG00000092199	HNRNPC	heterogeneous nuclear ribonucleoprotein C
ENSG00000092470	WDR76	WD repeat domain 76
ENSG00000092978	GPATCH2	G-patch domain containing 2
ENSG00000093167	LRRFIP2	LRR binding FLII interacting protein 2
ENSG00000094804	CDC6	cell division cycle 6
ENSG00000095002	MSH2	mutS homolog 2
ENSG00000096063	SRPK1	SRSF protein kinase 1
ENSG00000096746	HNRNPH3	heterogeneous nuclear ribonucleoprotein H3
ENSG00000097046	CDC7	cell division cycle 7
ENSG00000099219	ERMP1	endoplasmic reticulum metallopeptidase 1
ENSG00000099256	PRTFDC1	phosphoribosyl transferase domain containing 1
ENSG00000100003	SEC14L2	SEC14 like lipid binding 2
ENSG00000100024	UPB1	beta-ureidopropionase 1
ENSG00000100211	CBY1	chibby family member 1, beta catenin antagonist
ENSG00000100296	THOC5	THO complex 5
ENSG00000100473	COCH	cochlin
ENSG00000100478	AP4S1	adaptor related protein complex 4 subunit sigma 1
ENSG00000100483	VCPKMT	valosin containing protein lysine methyltransferase
ENSG00000100519	PSMC6	proteasome 26S subunit, ATPase 6
ENSG00000100522	GNPNAT1	glucosamine-phosphate N-acetyltransferase 1
ENSG00000100629	CEP128	centrosomal protein 128
ENSG00000100644	HIF1A	hypoxia inducible factor 1 subunit alpha
ENSG00000100650	SRSF5	serine and arginine rich splicing factor 5
ENSG00000100722	ZC3H14	zinc finger CCCH-type containing 14
ENSG00000100731	PCNX1	pecanex 1
ENSG00000100749	VRK1	VRK serine/threonine kinase 1
ENSG00000100784	RPS6KA5	ribosomal protein S6 kinase A5
ENSG00000100888	CHD8	chromodomain helicase DNA binding protein 8
ENSG00000100941	PNN	pinin, desmosome associated protein
ENSG00000101109	STK4	serine/threonine kinase 4
ENSG00000101161	PRPF6	pre-mRNA processing factor 6
ENSG00000101166	PRELID3B	PRELI domain containing 3B
ENSG00000101447	FAM83D	family with sequence similarity 83 member D
ENSG00000101460	MAP1LC3A	microtubule associated protein 1 light chain 3 alpha
ENSG00000101605	MYOM1	myomesin 1

ENSG00000101868	POLA1	DNA polymerase alpha 1, catalytic subunit
ENSG00000101901	ALG13	ALG13 UDP-N-acetylglucosaminyltransferase subunit
ENSG00000101966	XIAP	X-linked inhibitor of apoptosis
ENSG00000101974	ATP11C	ATPase phospholipid transporting 11C
ENSG00000102038	SMARCA1	SWI/SNF related, matrix associated, actin dependent regulator of chromatin, subfamily a, member 1
ENSG00000102043	MTMR8	myotubularin related protein 8
ENSG00000102053	ZC3H12B	zinc finger CCCH-type containing 12B
ENSG00000102078	SLC25A14	solute carrier family 25 member 14
ENSG00000102098	SCML2	Scm polycomb group protein like 2
ENSG00000102317	RBM3	RNA binding motif protein 3
ENSG00000102384	CENPI	centromere protein I
ENSG00000102390	PBDC1	polysaccharide biosynthesis domain containing 1
ENSG00000102445	RUBCNL	rubicon like autophagy enhancer
ENSG00000102580	DNAJC3	DnaJ heat shock protein family (Hsp40) member C3
ENSG00000102763	VWA8	von Willebrand factor A domain containing 8
ENSG00000102780	DGKH	diacylglycerol kinase eta
ENSG00000102781	KATNAL1	katanin catalytic subunit A1 like 1
ENSG00000102893	PHKB	phosphorylase kinase regulatory subunit beta
ENSG00000102908	NFAT5	nuclear factor of activated T cells 5
ENSG00000103494	RPGRIP1L	RPGRIP1 like
ENSG00000103657	HERC1	HECT and RLD domain containing E3 ubiquitin protein ligase family member 1
ENSG00000103978	TMEM87A	transmembrane protein 87A
ENSG00000103995	CEP152	centrosomal protein 152
ENSG00000104154	SLC30A4	solute carrier family 30 member 4
ENSG00000104177	MYEF2	myelin expression factor 2
ENSG00000104290	FZD3	frizzled class receptor 3
ENSG00000104321	TRPA1	transient receptor potential cation channel subfamily A member 1
ENSG00000104356	POP1	POP1 homolog, ribonuclease P/MRP subunit
ENSG00000104442	ARMC1	armadillo repeat containing 1
ENSG00000104517	UBR5	ubiquitin protein ligase E3 component n-recognin 5
ENSG00000104613	INTS10	integrator complex subunit 10
ENSG00000104626	ERI1	exoribonuclease 1
ENSG00000104714	ERICH1	glutamate rich 1
ENSG00000104974	LILRA1	leukocyte immunoglobulin like receptor A1
ENSG00000105127	AKAP8	A-kinase anchoring protein 8
ENSG00000105176	URI1	URI1 prefoldin like chaperone
ENSG00000105639	JAK3	Janus kinase 3
ENSG00000105793	GTPBP10	GTP binding protein 10
ENSG00000105835	NAMPT	nicotinamide phosphoribosyltransferase
ENSG00000105855	ITGB8	integrin subunit beta 8
ENSG00000105948	TTC26	tetratricopeptide repeat domain 26
ENSG00000105976	MET	MET proto-oncogene, receptor tyrosine kinase

ENSG00000106344	RBM28	RNA binding motif protein 28
ENSG00000106443	PHF14	PHD finger protein 14
ENSG00000106462	EZH2	enhancer of zeste 2 polycomb repressive complex 2 subunit
ENSG00000106524	ANKMY2	ankyrin repeat and MYND domain containing 2
ENSG00000106526	ACTR3C	actin related protein 3C
ENSG00000106537	TSPAN13	tetraspanin 13
ENSG00000106603	COA1	cytochrome c oxidase assembly factor 1 homolog
ENSG00000106605	BLVRA	biliverdin reductase A
ENSG00000106610	STAG3L4	stromal antigen 3-like 4 (pseudogene)
ENSG00000106692	FKTN	fukutin
ENSG00000106701	FSD1L	fibronectin type III and SPRY domain containing 1 like
ENSG00000106723	SPIN1	spindlin 1
ENSG00000106799	TGFBR1	transforming growth factor beta receptor 1
ENSG00000106804	C5	complement C5
ENSG00000106819	ASPN	asporin
ENSG00000106823	ECM2	extracellular matrix protein 2
ENSG00000107077	KDM4C	lysine demethylase 4C
ENSG00000107611	CUBN	cubilin
ENSG00000107614	TRDMT1	tRNA aspartic acid methyltransferase 1
ENSG00000107625	DDX50	DExD-box helicase 50
ENSG00000107758	PPP3CB	protein phosphatase 3 catalytic subunit beta
ENSG00000107938	EDRF1	erythroid differentiation regulatory factor 1
ENSG00000108021	TASOR2	transcription activation suppressor family member 2
ENSG00000108055	SMC3	structural maintenance of chromosomes 3
ENSG00000108395	TRIM37	tripartite motif containing 37
ENSG00000108443	RPS6KB1	ribosomal protein S6 kinase B1
ENSG00000108559	NUP88	nucleoporin 88
ENSG00000108651	UTP6	UTP6 small subunit processome component
ENSG00000108666	C17orf75	chromosome 17 open reading frame 75
ENSG00000108702	CCL1	C-C motif chemokine ligand 1
ENSG00000108848	LUC7L3	LUC7 like 3 pre-mRNA splicing factor
ENSG00000108958		succinate dehydrogenase complex, subunit C, integral membrane protein, 15kDa (SDHC) pseudogene
ENSG00000108984	MAP2K6	mitogen-activated protein kinase kinase 6
ENSG00000109436	TBC1D9	TBC1 domain family member 9
ENSG00000109452	INPP4B	inositol polyphosphate-4-phosphatase type II B
ENSG00000109685	NSD2	nuclear receptor binding SET domain protein 2
ENSG00000109771	LRP2BP	LRP2 binding protein
ENSG00000109805	NCAPG	non-SMC condensin I complex subunit G
ENSG00000110066	KMT5B	lysine methyltransferase 5B
ENSG00000110074	FOXRED1	FAD dependent oxidoreductase domain containing 1
ENSG00000110315	RNF141	ring finger protein 141
ENSG00000110318	CEP126	centrosomal protein 126

ENSG00000110367	DDX6	DEAD-box helicase 6
ENSG00000110436	SLC1A2	solute carrier family 1 member 2
ENSG00000110841	PPFIBP1	PPFIA binding protein 1
ENSG00000110848	CD69	CD69 molecule
ENSG00000110852	CLEC2B	C-type lectin domain family 2 member B
ENSG00000110888	CAPRIN2	caprin family member 2
ENSG00000111196	MAGOHB	mago homolog B, exon junction complex subunit
ENSG00000111224	PARP11	poly(ADP-ribose) polymerase family member 11
ENSG00000111247	RAD51AP1	RAD51 associated protein 1
ENSG00000111300	NAA25	N-alpha-acetyltransferase 25, NatB auxiliary subunit
ENSG00000111341	MGP	matrix Gla protein
ENSG00000111554	MDM1	Mdm1 nuclear protein
ENSG00000111666	CHPT1	choline phosphotransferase 1
ENSG00000111707	SUDS3	SDS3 homolog, SIN3A corepressor complex component
ENSG00000111731	C2CD5	C2 calcium dependent domain containing 5
ENSG00000111790	FGFR1OP2	FGFR1 oncogene partner 2
ENSG00000111834	RSPH4A	radial spoke head component 4A
ENSG00000111850	SMIM8	small integral membrane protein 8
ENSG00000111860	CEP85L	centrosomal protein 85 like
ENSG00000111877	MCM9	minichromosome maintenance 9 homologous recombination repair factor
ENSG00000111879	FAM184A	family with sequence similarity 184 member A
ENSG00000111880	RNGTT	RNA guanylyltransferase and 5'-phosphatase
ENSG00000112078	KCTD20	potassium channel tetramerization domain containing 20
ENSG00000112159	MDN1	midasin AAA ATPase 1
ENSG00000112210	RAB23	RAB23, member RAS oncogene family
ENSG00000112232	KHDRBS2	KH RNA binding domain containing, signal transduction associated 2
ENSG00000112234	FBXL4	F-box and leucine rich repeat protein 4
ENSG00000112297	CRYBG1	crystallin beta-gamma domain containing 1
ENSG00000112319	EYA4	EYA transcriptional coactivator and phosphatase 4
ENSG00000112414	ADGRG6	adhesion G protein-coupled receptor G6
ENSG00000112584	FAM120B	family with sequence similarity 120B
ENSG00000112624	BICRAL	BRD4 interacting chromatin remodeling complex associated protein like
ENSG00000112679	DUSP22	dual specificity phosphatase 22
ENSG00000112699	GMDS	GDP-mannose 4,6-dehydratase
ENSG00000112763	BTN2A1	butyrophilin subfamily 2 member A1
ENSG00000112799	LY86	lymphocyte antigen 86
ENSG00000113302	IL12B	interleukin 12B
ENSG00000113318	MSH3	mutS homolog 3
ENSG00000113360	DROSHA	drosha ribonuclease III
ENSG00000113448	PDE4D	phosphodiesterase 4D
ENSG00000113558	SKP1	S-phase kinase associated protein 1
ENSG00000113593	PPWD1	peptidylprolyl isomerase domain and WD repeat containing 1

ENSG00000113595	TRIM23	tripartite motif containing 23
ENSG00000113657	DPYSL3	dihydropyrimidinase like 3
ENSG00000114098	ARMC8	armadillo repeat containing 8
ENSG00000114107	CEP70	centrosomal protein 70
ENSG00000114166	KAT2B	lysine acetyltransferase 2B
ENSG00000114439	BBX	BBX high mobility group box domain containing
ENSG00000114446	IFT57	intraflagellar transport 57
ENSG00000114455	HHLA2	HERV-H LTR-associating 2
ENSG00000114650	SCAP	SREBF chaperone
ENSG00000114670	NEK11	NIMA related kinase 11
ENSG00000114933	INO80D	INO80 complex subunit D
ENSG00000114948	ADAM23	ADAM metallopeptidase domain 23
ENSG00000115008	IL1A	interleukin 1 alpha
ENSG00000115009	CCL20	C-C motif chemokine ligand 20
ENSG00000115109	EPB41L5	erythrocyte membrane protein band 4.1 like 5
ENSG00000115290	GRB14	growth factor receptor bound protein 14
ENSG00000115339	GALNT3	polypeptide N-acetylgalactosaminyltransferase 3
ENSG00000115364	MRPL19	mitochondrial ribosomal protein L19
ENSG00000115419	GLS	glutaminase
ENSG00000115464	USP34	ubiquitin specific peptidase 34
ENSG00000115486	GGCX	gamma-glutamyl carboxylase
ENSG00000115504	EHBP1	EH domain binding protein 1
ENSG00000115540	MOB4	MOB family member 4, phocein
ENSG00000115760	BIRC6	baculoviral IAP repeat containing 6
ENSG00000115828	QPCT	glutaminyl-peptide cyclotransferase
ENSG00000115839	RAB3GAP1	RAB3 GTPase activating protein catalytic subunit 1
ENSG00000115841	RMDN2	regulator of microtubule dynamics 2
ENSG00000115904	SOS1	SOS Ras/Rac guanine nucleotide exchange factor 1
ENSG00000115970	THADA	THADA armadillo repeat containing
ENSG00000116062	MSH6	mutS homolog 6
ENSG00000116095	PLEKHA3	pleckstrin homology domain containing A3
ENSG00000116106	EPHA4	EPH receptor A4
ENSG00000116127	ALMS1	ALMS1 centrosome and basal body associated protein
ENSG00000116191	RALGPS2	Ral GEF with PH domain and SH3 binding motif 2
ENSG00000116580	GON4L	gon-4 like
ENSG00000116667	C1orf21	chromosome 1 open reading frame 21
ENSG00000116690	PRG4	proteoglycan 4
ENSG00000116703	PDC	phosducin
ENSG00000116747	RO60	Ro60, Y RNA binding protein
ENSG00000116750	UCHL5	ubiquitin C-terminal hydrolase L5
ENSG00000116833	NR5A2	nuclear receptor subfamily 5 group A member 2
ENSG00000116874	WARS2	tryptophanyl tRNA synthetase 2, mitochondrial
ENSG00000116906	GNPAT	glyceronephosphate O-acyltransferase

ENSG00000116954	RRAGC	Ras related GTP binding C
ENSG00000117010	ZNF684	zinc finger protein 684
ENSG00000117020	AKT3	AKT serine/threonine kinase 3
ENSG00000117143	UAP1	UDP-N-acetylglucosamine pyrophosphorylase 1
ENSG00000117155	SSX2IP	SSX family member 2 interacting protein
ENSG00000117528	ABCD3	ATP binding cassette subfamily D member 3
ENSG00000117569	PTBP2	polypyrimidine tract binding protein 2
ENSG00000117697	NSL1	NSL1 component of MIS12 kinetochore complex
ENSG00000117724	CENPF	centromere protein F
ENSG00000117859	OSBPL9	oxysterol binding protein like 9
ENSG00000118007	STAG1	stromal antigen 1
ENSG00000118420	UBE3D	ubiquitin protein ligase E3D
ENSG00000118496	FBXO30	F-box protein 30
ENSG00000118507	AKAP7	A-kinase anchoring protein 7
ENSG00000118564	FBXL5	F-box and leucine rich repeat protein 5
ENSG00000118596	SLC16A7	solute carrier family 16 member 7
ENSG00000118690	ARMC2	armadillo repeat containing 2
ENSG00000118922	KLF12	Kruppel like factor 12
ENSG00000119041	GTF3C3	general transcription factor IIIC subunit 3
ENSG00000119048	UBE2B	ubiquitin conjugating enzyme E2 B
ENSG00000119231	SENP5	SUMO specific peptidase 5
ENSG00000119285	HEATR1	HEAT repeat containing 1
ENSG00000119403	PHF19	PHD finger protein 19
ENSG00000119509	INVS	inversin
ENSG00000119599	DCAF4	DDB1 and CUL4 associated factor 4
ENSG00000119636	BBOF1	basal body orientation factor 1
ENSG00000119685	TTLL5	tubulin tyrosine ligase like 5
ENSG00000119707	RBM25	RNA binding motif protein 25
ENSG00000119711	ALDH6A1	aldehyde dehydrogenase 6 family member A1
ENSG00000119720	NRDE2	NRDE-2, necessary for RNA interference, domain containing
ENSG00000119778	ATAD2B	ATPase family AAA domain containing 2B
ENSG00000119906	SLF2	SMC5-SMC6 complex localization factor 2
ENSG00000119969	HELLS	helicase, lymphoid specific
ENSG00000120519	SLC10A7	solute carrier family 10 member 7
ENSG00000120733	KDM3B	lysine demethylase 3B
ENSG00000120802	TMPO	thymopoietin
ENSG00000120963	ZNF706	zinc finger protein 706
ENSG00000121022	COPS5	COP9 signalosome subunit 5
ENSG00000121152	NCAPH	non-SMC condensin I complex subunit H
ENSG00000121210	TMEM131L	transmembrane 131 like
ENSG00000121289	CEP89	centrosomal protein 89
ENSG00000121390	PSPC1	paraspeckle component 1
ENSG00000121621	KIF18A	kinesin family member 18A

ENSG00000121851	POLR3GL	RNA polymerase III subunit GL
ENSG00000121895	TMEM156	transmembrane protein 156
ENSG00000121957	GPSM2	G protein signaling modulator 2
ENSG00000121988	ZRANB3	zinc finger RANBP2-type containing 3
ENSG00000122257	RBBP6	RB binding protein 6, ubiquitin ligase
ENSG00000122417	ODF2L	outer dense fiber of sperm tails 2 like
ENSG00000122482	ZNF644	zinc finger protein 644
ENSG00000122483	CCDC18	coiled-coil domain containing 18
ENSG00000122507	BBS9	Bardet-Biedl syndrome 9
ENSG00000122512	PMS2	PMS1 homolog 2, mismatch repair system component
ENSG00000122566	HNRNPA2B1	heterogeneous nuclear ribonucleoprotein A2/B1
ENSG00000122872	ARL4AP1	ADP ribosylation factor like GTPase 4A pseudogene 1
ENSG00000122970	IFT81	intraflagellar transport 81
ENSG00000123106	CCDC91	coiled-coil domain containing 91
ENSG00000123219	CENPK	centromere protein K
ENSG00000123607	TTC21B	tetratricopeptide repeat domain 21B
ENSG00000123636	BAZ2B	bromodomain adjacent to zinc finger domain 2B
ENSG00000123684	LPGAT1	lysophosphatidylglycerol acyltransferase 1
ENSG00000123689	G0S2	G0/G1 switch 2
ENSG00000123870	ZNF137P	zinc finger protein 137, pseudogene
ENSG00000124215	CDH26	cadherin 26
ENSG00000124479	NDP	norrin cystine knot growth factor NDP
ENSG00000124486	USP9X	ubiquitin specific peptidase 9 X-linked
ENSG00000124532	MRS2	magnesium transporter MRS2
ENSG00000124564	SLC17A3	solute carrier family 17 member 3
ENSG00000124813	RUNX2	RUNX family transcription factor 2
ENSG00000125257	ABCC4	ATP binding cassette subfamily C member 4
ENSG00000125351	UPF3B	UPF3B regulator of nonsense mediated mRNA decay
ENSG00000125356	NDUFA1	NADH:ubiquinone oxidoreductase subunit A1
ENSG00000125482	TTF1	transcription termination factor 1
ENSG00000125629	INSIG2	insulin induced gene 2
ENSG00000125633	CCDC93	coiled-coil domain containing 93
ENSG00000125652	ALKBH7	alkB homolog 7
ENSG00000125691	RPL23	ribosomal protein L23
ENSG00000125772	GPCPD1	glycerophosphocholine phosphodiesterase 1
ENSG00000125810	CD93	CD93 molecule
ENSG00000125823	CSTL1	cystatin like 1
ENSG00000125834	STK35	serine/threonine kinase 35
ENSG00000125903	DEFB129	defensin beta 129
ENSG00000125931	CITED1	Cbp/p300 interacting transactivator with Glu/Asp rich carboxy-terminal domain 1
ENSG00000126070	AGO3	argonaute RISC catalytic component 3
ENSG00000126653	NSRP1	nuclear speckle splicing regulatory protein 1

ENSG00000126787	DLGAP5	DLG associated protein 5
ENSG00000126790	L3HYPDH	trans-L-3-hydroxyproline dehydratase
ENSG00000126945	HNRNPH2	heterogeneous nuclear ribonucleoprotein H2
ENSG00000127081	ZNF484	zinc finger protein 484
ENSG00000127314	RAP1B	RAP1B, member of RAS oncogene family
ENSG00000127366	TAS2R5	taste 2 receptor member 5
ENSG00000127603	MACF1	microtubule actin crosslinking factor 1
ENSG00000127720	METTL25	methyltransferase like 25
ENSG00000127914	AKAP9	A-kinase anchoring protein 9
ENSG00000127922	SEM1	SEM1 26S proteasome complex subunit
ENSG00000127990	SGCE	sarcoglycan epsilon
ENSG00000127995	CASD1	CAS1 domain containing 1
ENSG00000128059	PPAT	phosphoribosyl pyrophosphate amidotransferase
ENSG00000128534	LSM8	LSM8 homolog, U6 small nuclear RNA associated
ENSG00000128536	CDHR3	cadherin related family member 3
ENSG00000128585	MKLN1	muskelin 1
ENSG00000128609	NDUFA5	NADH:ubiquinone oxidoreductase subunit A5
ENSG00000128641	MYO1B	myosin IB
ENSG00000128654	MTX2	metaxin 2
ENSG00000128731	HERC2	HECT and RLD domain containing E3 ubiquitin protein ligase 2
ENSG00000129003	VPS13C	vacuolar protein sorting 13 homolog C
ENSG00000129167	TPH1	tryptophan hydroxylase 1
ENSG00000129235	TXNDC17	thioredoxin domain containing 17
ENSG00000129292	PHF20L1	PHD finger protein 20 like 1
ENSG00000129675	ARHGEF6	Rac/Cdc42 guanine nucleotide exchange factor 6
ENSG00000129810	SGO1	shugoshin 1
ENSG00000130023	ERMARD	ER membrane associated RNA degradation
ENSG00000130347	RTN4IP1	reticulon 4 interacting protein 1
ENSG00000130826	DKC1	dyskerin pseudouridine synthase 1
ENSG00000130856	ZNF236	zinc finger protein 236
ENSG00000130939	UBE4B	ubiquitination factor E4B
ENSG00000130997	POLN	DNA polymerase nu
ENSG00000131127	ZNF141	zinc finger protein 141
ENSG00000131269	ABCB7	ATP binding cassette subfamily B member 7
ENSG00000131373	HACL1	2-hydroxyacyl-CoA lyase 1
ENSG00000131374	TBC1D5	TBC1 domain family member 5
ENSG00000131375	CAPN7	calpain 7
ENSG00000131437	KIF3A	kinesin family member 3A
ENSG00000131558	EXOC4	exocyst complex component 4
ENSG00000131626	PPFIA1	PTPRF interacting protein alpha 1
ENSG00000131871	SELENOS	selenoprotein S
ENSG00000132141	CCT6B	chaperonin containing TCP1 subunit 6B
ENSG00000132394	EEFSEC	eukaryotic elongation factor, selenocysteine-tRNA specific

ENSG00000132424	PNISR	PNN interacting serine and arginine rich protein
ENSG00000132432	SEC61G	SEC61 translocon subunit gamma
ENSG00000132436	FIGNL1	fidgetin like 1
ENSG00000132549	VPS13B	vacuolar protein sorting 13 homolog B
ENSG00000132704	FCRL2	Fc receptor like 2
ENSG00000132780	NASP	nuclear autoantigenic sperm protein
ENSG00000132842	AP3B1	adaptor related protein complex 3 subunit beta 1
ENSG00000132849	PATJ	PATJ crumbs cell polarity complex component
ENSG00000133030	MPRIP	myosin phosphatase Rho interacting protein
ENSG00000133059	DSTYK	dual serine/threonine and tyrosine protein kinase
ENSG00000133103	COG6	component of oligomeric golgi complex 6
ENSG00000133119	RFC3	replication factor C subunit 3
ENSG00000133302	SLF1	SMC5-SMC6 complex localization factor 1
ENSG00000133398	MED10	mediator complex subunit 10
ENSG00000133703	KRAS	KRAS proto-oncogene, GTPase
ENSG00000133704	IPO8	importin 8
ENSG00000133812	SBF2	SET binding factor 2
ENSG00000133858	ZFC3H1	zinc finger C3H1-type containing
ENSG00000133962	CATSPERB	cation channel sperm associated auxiliary subunit beta
ENSG00000133997	MED6	mediator complex subunit 6
ENSG00000134028	ADAMDEC1	ADAM like decysin 1
ENSG00000134057	CCNB1	cyclin B1
ENSG00000134152	KATNBL1	katanin regulatory subunit B1 like 1
ENSG00000134200	TSHB	thyroid stimulating hormone subunit beta
ENSG00000134283	PPHLN1	periphilin 1
ENSG00000134297	PLEKHA8P1	pleckstrin homology domain containing A8 pseudogene 1
ENSG00000134313	KIDINS220	kinase D interacting substrate 220
ENSG00000134318	ROCK2	Rho associated coiled-coil containing protein kinase 2
ENSG00000134330	IAH1	isoamyl acetate hydrolyzing esterase 1 (putative)
ENSG00000134371	CDC73	cell division cycle 73
ENSG00000134453	RBM17	RNA binding motif protein 17
ENSG00000134480	CCNH	cyclin H
ENSG00000134490	TMEM241	transmembrane protein 241
ENSG00000134508	CABLES1	Cdk5 and Abl enzyme substrate 1
ENSG00000134532	SOX5	SRY-box transcription factor 5
ENSG00000134602	STK26	serine/threonine kinase 26
ENSG00000134709	HOOK1	hook microtubule tethering protein 1
ENSG00000134744	TUT4	terminal uridylyl transferase 4
ENSG00000134905	CARS2	cysteinyl-tRNA synthetase 2, mitochondrial
ENSG00000134909	ARHGAP32	Rho GTPase activating protein 32
ENSG00000135040	NAA35	N-alpha-acetyltransferase 35, NatC auxiliary subunit
ENSG00000135074	ADAM19	ADAM metallopeptidase domain 19
ENSG00000135090	TAOK3	TAO kinase 3

ENSG00000135164	DMTF1	cyclin D binding myb like transcription factor 1
ENSG00000135249	RINT1	RAD50 interactor 1
ENSG00000135315	CEP162	centrosomal protein 162
ENSG00000135318	NT5E	5'-nucleotidase ecto
ENSG00000135341	MAP3K7	mitogen-activated protein kinase kinase kinase 7
ENSG00000135436	FAM186B	family with sequence similarity 186 member B
ENSG00000135457	TFCP2	transcription factor CP2
ENSG00000135537	AFG1L	AFG1 like ATPase
ENSG00000135597	REPS1	RALBP1 associated Eps domain containing 1
ENSG00000135622	SEMA4F	ssemaphorin 4F
ENSG00000135678	CPM	carboxypeptidase M
ENSG00000135720	DYNC1LI2	dynein cytoplasmic 1 light intermediate chain 2
ENSG00000135837	CEP350	centrosomal protein 350
ENSG00000135842	NIBAN1	niban apoptosis regulator 1
ENSG00000135945	REV1	REV1 DNA directed polymerase
ENSG00000135951	TSGA10	testis specific 10
ENSG00000135972	MRPS9	mitochondrial ribosomal protein S9
ENSG00000135976	ANKRD36	ankyrin repeat domain 36
ENSG00000136010	ALDH1L2	aldehyde dehydrogenase 1 family member L2
ENSG00000136045	PWP1	PWP1 homolog, endonuclein
ENSG00000136051	WASHC4	WASH complex subunit 4
ENSG00000136100	VPS36	vacuolar protein sorting 36 homolog
ENSG00000136152	COG3	component of oligomeric golgi complex 3
ENSG00000136231	IGF2BP3	insulin like growth factor 2 mRNA binding protein 3
ENSG00000136244	IL6	interleukin 6
ENSG00000136250	AOAH	acyloxyacyl hydrolase
ENSG00000136286	MYO1G	myosin IG
ENSG00000136319	TTC5	tetratricopeptide repeat domain 5
ENSG00000136425	CIB2	calcium and integrin binding family member 2
ENSG00000136492	BRIP1	BRCA1 interacting protein C-terminal helicase 1
ENSG00000136504	KAT7	lysine acetyltransferase 7
ENSG00000136541	ERMN	ermin
ENSG00000136643	RPS6KC1	ribosomal protein S6 kinase C1
ENSG00000136688	IL36G	interleukin 36 gamma
ENSG00000136731	UGGT1	UDP-glucose glycoprotein glucosyltransferase 1
ENSG00000136824	SMC2	structural maintenance of chromosomes 2
ENSG00000136861	CDK5RAP2	CDK5 regulatory subunit associated protein 2
ENSG00000136895	GARNL3	GTPase activating Rap/RanGAP domain like 3
ENSG00000136933	RABEPK	Rab9 effector protein with kelch motifs
ENSG00000137193	PIM1	Pim-1 proto-oncogene, serine/threonine kinase
ENSG00000137331	IER3	immediate early response 3
ENSG00000137563	GGH	gamma-glutamyl hydrolase
ENSG00000137601	NEK1	NIMA related kinase 1

ENSG00000137673	MMP7	matrix metallopeptidase 7
ENSG00000137713	PPP2R1B	protein phosphatase 2 scaffold subunit Abeta
ENSG00000137757	CASP5	caspase 5
ENSG00000137760	ALKBH8	alkB homolog 8, tRNA methyltransferase
ENSG00000137764	MAP2K5	mitogen-activated protein kinase kinase 5
ENSG00000137807	KIF23	kinesin family member 23
ENSG00000137812	KNL1	kinetochore scaffold 1
ENSG00000137822	TUBGCP4	tubulin gamma complex associated protein 4
ENSG00000137876	RSL24D1	ribosomal L24 domain containing 1
ENSG00000138092	CENPO	centromere protein O
ENSG00000138115	CYP2C8	cytochrome P450 family 2 subfamily C member 8
ENSG00000138134	STAMBPL1	STAM binding protein like 1
ENSG00000138160	KIF11	kinesin family member 11
ENSG00000138182	KIF20B	kinesin family member 20B
ENSG00000138190	EXOC6	exocyst complex component 6
ENSG00000138336	TET1	tet methylcytosine dioxygenase 1
ENSG00000138346	DNA2	DNA replication helicase/nuclease 2
ENSG00000138376	BARD1	BRCA1 associated RING domain 1
ENSG00000138398	PPIG	peptidylprolyl isomerase G
ENSG00000138468	SENP7	SUMO specific peptidase 7
ENSG00000138658	ZGRF1	zinc finger GRF-type containing 1
ENSG00000138709	LARP1B	La ribonucleoprotein 1B
ENSG00000138780	GSTCD	glutathione S-transferase C-terminal domain containing
ENSG00000138814	PPP3CA	protein phosphatase 3 catalytic subunit alpha
ENSG00000138821	SLC39A8	solute carrier family 39 member 8
ENSG00000139154	AEBP2	AE binding protein 2
ENSG00000139324	TMTC3	transmembrane O-mannosyltransferase targeting cadherins 3
ENSG00000139350	NEDD1	NEDD1 gamma-tubulin ring complex targeting factor
ENSG00000139351	SYCP3	synaptonemal complex protein 3
ENSG00000139372	TDG	thymine DNA glycosylase
ENSG00000139697	SBNO1	strawberry notch homolog 1
ENSG00000139726	DENR	density regulated re-initiation and release factor
ENSG00000139734	DIAPH3	diaphanous related formin 3
ENSG00000140386	SCAPER	S-phase cyclin A associated protein in the ER
ENSG00000140525	FANCI	FA complementation group I
ENSG00000140598	EFL1	elongation factor like GTPase 1
ENSG00000140718	FTO	FTO alpha-ketoglutarate dependent dioxygenase
ENSG00000140993	TIGD7	tigger transposable element derived 7
ENSG00000141198	TOM1L1	target of myb1 like 1 membrane trafficking protein
ENSG00000141376	BCAS3	BCAS3 microtubule associated cell migration factor
ENSG00000141425	RPRD1A	regulation of nuclear pre-mRNA domain containing 1A
ENSG00000141446	ESCO1	establishment of sister chromatid cohesion N-acetyltransferase 1
ENSG00000141646	SMAD4	SMAD family member 4

ENSG00000141665	FBXO15	F-box protein 15
ENSG00000141968	VAV1	vav guanine nucleotide exchange factor 1
ENSG00000142168	SOD1	superoxide dismutase 1
ENSG00000142224	IL19	interleukin 19
ENSG00000142864	SERBP1	SERPINE1 mRNA binding protein 1
ENSG00000142875	PRKACB	protein kinase cAMP-activated catalytic subunit beta
ENSG00000142892	PIGK	phosphatidylinositol glycan anchor biosynthesis class K
ENSG00000143033	MTF2	metal response element binding transcription factor 2
ENSG00000143149	ALDH9A1	aldehyde dehydrogenase 9 family member A1
ENSG00000143155	TIPRL	TOR signaling pathway regulator
ENSG00000143156	NME7	NME/NM23 family member 7
ENSG00000143164	DCAF6	DDB1 and CUL4 associated factor 6
ENSG00000143228	NUF2	NUF2 component of NDC80 kinetochore complex
ENSG00000143248	RGS5	regulator of G protein signaling 5
ENSG00000143324	XPR1	xenotropic and polytropic retrovirus receptor 1
ENSG00000143353	LYPLAL1	lysophospholipase like 1
ENSG00000143401	ANP32E	acidic nuclear phosphoprotein 32 family member E
ENSG00000143458	GABPB2	GA binding protein transcription factor subunit beta 2
ENSG00000143476	DTL	denticleless E3 ubiquitin protein ligase homolog
ENSG00000143493	INTS7	integrator complex subunit 7
ENSG00000143499	SMYD2	SET and MYND domain containing 2
ENSG00000143815	LBR	lamin B receptor
ENSG00000143947	RPS27A	ribosomal protein S27a
ENSG00000143951	WDPCP	WD repeat containing planar cell polarity effector
ENSG00000143954	REG3G	regenerating family member 3 gamma
ENSG00000144029	MRPS5	mitochondrial ribosomal protein S5
ENSG00000144036	EXOC6B	exocyst complex component 6B
ENSG00000144061	NPHP1	nephrocystin 1
ENSG00000144228	SPOPL	speckle type BTB/POZ protein like
ENSG00000144357	UBR3	ubiquitin protein ligase E3 component n-recognin 3
ENSG00000144366	GULP1	GULP PTB domain containing engulfment adaptor 1
ENSG00000144426	NBEAL1	neurobeachin like 1
ENSG00000144535	DIS3L2	DIS3 like 3'-5' exoribonuclease 2
ENSG00000144554	FANCD2	FA complementation group D2
ENSG00000144741	SLC25A26	solute carrier family 25 member 26
ENSG00000144815	NXPE3	neurexophilin and PC-esterase domain family member 3
ENSG00000145012	LPP	LIM domain containing preferred translocation partner in lipoma
ENSG00000145216	FIP1L1	factor interacting with PAPOLA and CPSF1
ENSG00000145241	CENPC	centromere protein C
ENSG00000145331	TRMT10A	tRNA methyltransferase 10A
ENSG00000145332	KLHL8	kelch like family member 8
ENSG00000145375	SPATA5	spermatogenesis associated 5
ENSG00000145386	CCNA2	cyclin A2

ENSG00000145390	USP53	ubiquitin specific peptidase 53
ENSG00000145494	NDUFS6	NADH:ubiquinone oxidoreductase subunit S6
ENSG00000145604	SKP2	S-phase kinase associated protein 2
ENSG00000145700	ANKRD31	ankyrin repeat domain 31
ENSG00000145725	PPIP5K2	diphosphoinositol pentakisphosphate kinase 2
ENSG00000145734	BDP1	B double prime 1, subunit of RNA polymerase III transcription initiation factor IIIB
ENSG00000145779	TNFAIP8	TNF alpha induced protein 8
ENSG00000145781	COMMD10	COMM domain containing 10
ENSG00000145982	FARS2	phenylalanyl-tRNA synthetase 2, mitochondrial
ENSG00000145990	GFOD1	glucose-fructose oxidoreductase domain containing 1
ENSG00000145996	CDKAL1	CDK5 regulatory subunit associated protein 1 like 1
ENSG00000146143	PRIM2	DNA primase subunit 2
ENSG00000146247	PHIP	pleckstrin homology domain interacting protein
ENSG00000146263	MMS22L	MMS22 like, DNA repair protein
ENSG00000146282	RARS2	arginyl-tRNA synthetase 2, mitochondrial
ENSG00000146350	TBC1D32	TBC1 domain family member 32
ENSG00000146383	TAAR6	trace amine associated receptor 6
ENSG00000146410	MTFR2	mitochondrial fission regulator 2
ENSG00000146414	SHPRH	SNF2 histone linker PHD RING helicase
ENSG00000146676	PURB	purine rich element binding protein B
ENSG00000146731	CCT6A	chaperonin containing TCP1 subunit 6A
ENSG00000146802	TMEM168	transmembrane protein 168
ENSG00000146856	AGBL3	ATP/GTP binding protein like 3
ENSG00000146872	TLK2	tousled like kinase 2
ENSG00000147117	ZNF157	zinc finger protein 157
ENSG00000147118	ZNF182	zinc finger protein 182
ENSG00000147124	ZNF41	zinc finger protein 41
ENSG00000147133	TAF1	TATA-box binding protein associated factor 1
ENSG00000147145	LPAR4	lysophosphatidic acid receptor 4
ENSG00000147166	ITGB1BP2	integrin subunit beta 1 binding protein 2
ENSG00000147174	GCNA	germ cell nuclear acidic peptidase
ENSG00000147202	DIAPH2	diaphanous related formin 2
ENSG00000147231	RADX	RPA1 related single stranded DNA binding protein, X-linked
ENSG00000147274	RBMX	RNA binding motif protein X-linked
ENSG00000147316	MCPH1	microcephalin 1
ENSG00000147434	CHRNA6	cholinergic receptor nicotinic alpha 6 subunit
ENSG00000147437	GNRH1	gonadotropin releasing hormone 1
ENSG00000147459	DOCK5	dedicator of cytokinesis 5
ENSG00000147548	NSD3	nuclear receptor binding SET domain protein 3
ENSG00000147873	IFNA5	interferon alpha 5
ENSG00000147874	HAUS6	HAUS augmin like complex subunit 6
ENSG00000147905	ZCCHC7	zinc finger CCHC-type containing 7

ENSG00000148120	AOPEP	aminopeptidase O (putative)
ENSG00000148606	POLR3A	RNA polymerase III subunit A
ENSG00000148634	HERC4	HECT and RLD domain containing E3 ubiquitin protein ligase 4
ENSG00000148660	CAMK2G	calcium/calmodulin dependent protein kinase II gamma
ENSG00000148935	GAS2	growth arrest specific 2
ENSG00000149133	OR5F1	olfactory receptor family 5 subfamily F member 1
ENSG00000149262	INTS4	integrator complex subunit 4
ENSG00000149346	SLX4IP	SLX4 interacting protein
ENSG00000149554	CHEK1	checkpoint kinase 1
ENSG00000149635	OCSTAMP	osteoclast stimulatory transmembrane protein
ENSG00000150054	MPP7	membrane palmitoylated protein 7
ENSG00000150401	DCUN1D2	defective in cullin neddylation 1 domain containing 2
ENSG00000150433	TMEM218	transmembrane protein 218
ENSG00000150510	FAM124A	family with sequence similarity 124 member A
ENSG00000150593	PDCD4	programmed cell death 4
ENSG00000150627	WDR17	WD repeat domain 17
ENSG00000150667	FSIP1	fibrous sheath interacting protein 1
ENSG00000150756	ATPSCKMT	ATP synthase c subunit lysine N-methyltransferase
ENSG00000150776	NKAPD1	NKAP domain containing 1
ENSG00000150782	IL18	interleukin 18
ENSG00000150787	PTS	6-pyruvoyltetrahydropterin synthase
ENSG00000150938	CRIM1	cysteine rich transmembrane BMP regulator 1
ENSG00000150995	ITPR1	inositol 1,4,5-trisphosphate receptor type 1
ENSG00000151092	NGLY1	N-glycanase 1
ENSG00000151148	UBE3B	ubiquitin protein ligase E3B
ENSG00000151150	ANK3	ankyrin 3
ENSG00000151320	AKAP6	A-kinase anchoring protein 6
ENSG00000151327	FAM177A1	family with sequence similarity 177 member A1
ENSG00000151413	NUBPL	nucleotide binding protein like
ENSG00000151418	ATP6V1G3	ATPase H+ transporting V1 subunit G3
ENSG00000151422	FER	FER tyrosine kinase
ENSG00000151466	SCLT1	sodium channel and clathrin linker 1
ENSG00000151503	NCAPD3	non-SMC condensin II complex subunit D3
ENSG00000151532	VTI1A	vesicle transport through interaction with t-SNAREs 1A
ENSG00000151552	QDPR	quinoid dihydropteridine reductase
ENSG00000151657	KIN	Kin17 DNA and RNA binding protein
ENSG00000151687	ANKAR	ankyrin and armadillo repeat containing
ENSG00000151693	ASAP2	ArfGAP with SH3 domain, ankyrin repeat and PH domain 2
ENSG00000151725	CENPU	centromere protein U
ENSG00000151743	AMN1	antagonist of mitotic exit network 1 homolog
ENSG00000151746	BICD1	BICD cargo adaptor 1
ENSG00000151779	NBAS	NBAS subunit of NRZ tethering complex
ENSG00000151789	ZNF385D	zinc finger protein 385D

ENSG00000151835	SACS	sacsin molecular chaperone
ENSG00000151849	CENPJ	centromere protein J
ENSG00000151881	TMEM267	transmembrane protein 267
ENSG00000152061	RABGAP1L	RAB GTPase activating protein 1 like
ENSG00000152133	GPATCH11	G-patch domain containing 11
ENSG00000152193	OBI1	ORC ubiquitin ligase 1
ENSG00000152223	EPG5	ectopic P-granules autophagy protein 5 homolog
ENSG00000152234	ATP5F1A	ATP synthase F1 subunit alpha
ENSG00000152256	PDK1	pyruvate dehydrogenase kinase 1
ENSG00000152332	UHMK1	U2AF homology motif kinase 1
ENSG00000152348	ATG10	autophagy related 10
ENSG00000152404	CWF19L2	CWF19 like cell cycle control factor 2
ENSG00000152582	SPEF2	sperm flagellar 2
ENSG00000152620	NADK2	NAD kinase 2, mitochondrial
ENSG00000152661	GJA1	gap junction protein alpha 1
ENSG00000152818	UTRN	utrophin
ENSG00000152944	MED21	mediator complex subunit 21
ENSG00000152952	PLOD2	procollagen-lysine,2-oxoglutarate 5-dioxygenase 2
ENSG00000153015	CWC27	CWC27 spliceosome associated cyclophilin
ENSG00000153044	CENPH	centromere protein H
ENSG00000153064	BANK1	B cell scaffold protein with ankyrin repeats 1
ENSG00000153107	ANAPC1	anaphase promoting complex subunit 1
ENSG00000153132	CLGN	calmegin
ENSG00000153187	HNRNPU	heterogeneous nuclear ribonucleoprotein U
ENSG00000153253	SCN3A	sodium voltage-gated channel alpha subunit 3
ENSG00000153339	TRAPPC8	trafficking protein particle complex 8
ENSG00000153347	FAM81B	family with sequence similarity 81 member B
ENSG00000153363	LINC00467	long intergenic non-protein coding RNA 467
ENSG00000153561	RMND5A	required for meiotic nuclear division 5 homolog A
ENSG00000153774	CFDP1	craniofacial development protein 1
ENSG00000153898	MCOLN2	mucolipin 2
ENSG00000153914	SREK1	splicing regulatory glutamic acid and lysine rich protein 1
ENSG00000153944	MSI2	musashi RNA binding protein 2
ENSG00000154153	RETREG1	reticulophagy regulator 1
ENSG00000154188	ANGPT1	angiopoietin 1
ENSG00000154227	CERS3	ceramide synthase 3
ENSG00000154240	CEP112	centrosomal protein 112
ENSG00000154258	ABCA9	ATP binding cassette subfamily A member 9
ENSG00000154262	ABCA6	ATP binding cassette subfamily A member 6
ENSG00000154263	ABCA10	ATP binding cassette subfamily A member 10
ENSG00000154265	ABCA5	ATP binding cassette subfamily A member 5
ENSG00000154310	TNIK	TRAF2 and NCK interacting kinase
ENSG00000154473	BUB3	BUB3 mitotic checkpoint protein

ENSG00000154511	DIPK1A	divergent protein kinase domain 1A
ENSG00000154556	SORBS2	sorbin and SH3 domain containing 2
ENSG00000154655	L3MBTL4	L3MBTL histone methyl-lysine binding protein 4
ENSG00000154781	CCDC174	coiled-coil domain containing 174
ENSG00000154822	PLCL2	phospholipase C like 2
ENSG00000154845	PPP4R1	protein phosphatase 4 regulatory subunit 1
ENSG00000155011	DKK2	dickkopf WNT signaling pathway inhibitor 2
ENSG00000155085	AK9	adenylate kinase 9
ENSG00000155100	OTUD6B	OTU deubiquitinase 6B
ENSG00000155158	TTC39B	tetratricopeptide repeat domain 39B
ENSG00000155380	SLC16A1	solute carrier family 16 member 1
ENSG00000155465	SLC7A7	solute carrier family 7 member 7
ENSG00000155657	TTN	titin
ENSG00000155749	FLACC1	flagellum associated containing coiled-coil domains 1
ENSG00000155903	RASA2	RAS p21 protein activator 2
ENSG00000155970	MICU3	mitochondrial calcium uptake family member 3
ENSG00000156011	PSD3	pleckstrin and Sec7 domain containing 3
ENSG00000156042	CFAP70	cilia and flagella associated protein 70
ENSG00000156110	ADK	adenosine kinase
ENSG00000156172	C8orf37	chromosome 8 open reading frame 37
ENSG00000156256	USP16	ubiquitin specific peptidase 16
ENSG00000156313	RPGR	retinitis pigmentosa GTPase regulator
ENSG00000156467	UQCRB	ubiquinol-cytochrome c reductase binding protein
ENSG00000156469	MTERF3	mitochondrial transcription termination factor 3
ENSG00000156795	NTAQ1	N-terminal glutamine amidase 1
ENSG00000156802	ATAD2	ATPase family AAA domain containing 2
ENSG00000156876	SASS6	SAS-6 centriolar assembly protein
ENSG00000156958	GALK2	galactokinase 2
ENSG00000157036	EXOG	exo/endonuclease G
ENSG00000157106	SMG1	SMG1 nonsense mediated mRNA decay associated PI3K related kinase
ENSG00000157227	MMP14	matrix metallopeptidase 14
ENSG00000157653	C9orf43	chromosome 9 open reading frame 43
ENSG00000158019	BABAM2	BRISC and BRCA1 A complex member 2
ENSG00000158623	COPG2	COPI coat complex subunit gamma 2
ENSG00000158669	GPAT4	glycerol-3-phosphate acyltransferase 4
ENSG00000158683	PKD1L1	polycystin 1 like 1, transient receptor potential channel interacting
ENSG00000159055	MIS18A	MIS18 kinetochore protein A
ENSG00000159216	RUNX1	RUNX family transcription factor 1
ENSG00000159459	UBR1	ubiquitin protein ligase E3 component n-recognin 1
ENSG00000159579	RSPRY1	ring finger and SPRY domain containing 1
ENSG00000160124	CCDC58	coiled-coil domain containing 58
ENSG00000160179	ABCG1	ATP binding cassette subfamily G member 1

ENSG00000160285	LSS	lanosterol synthase
ENSG00000160326	SLC2A6	solute carrier family 2 member 6
ENSG00000160551	TAOK1	TAO kinase 1
ENSG00000161048	NAPEPLD	N-acyl phosphatidylethanolamine phospholipase D
ENSG00000161405	IKZF3	IKAROS family zinc finger 3
ENSG00000161912	ADCY10P1	ADCY10 pseudogene 1
ENSG00000162402	USP24	ubiquitin specific peptidase 24
ENSG00000162601	MYSM1	Myb like, SWIRM and MPN domains 1
ENSG00000162614	NEXN	nexilin F-actin binding protein
ENSG00000162688	AGL	amylo-alpha-1, 6-glucosidase, 4-alpha-glucanotransferase
ENSG00000162775	RBM15	RNA binding motif protein 15
ENSG00000162779	AXDND1	axonemal dynein light chain domain containing 1
ENSG00000162813	BPNT1	3'(2'), 5'-bisphosphate nucleotidase 1
ENSG00000162852	CNST	consortin, connexin sorting protein
ENSG00000162891	IL20	interleukin 20
ENSG00000162927	PUS10	pseudouridine synthase 10
ENSG00000162961	DPY30	dpy-30 histone methyltransferase complex regulatory subunit
ENSG00000162994	CLHC1	clathrin heavy chain linker domain containing 1
ENSG00000163002	NUP35	nucleoporin 35
ENSG00000163006	CCDC138	coiled-coil domain containing 138
ENSG00000163082	SGPP2	sphingosine-1-phosphate phosphatase 2
ENSG00000163106	HPGDS	hematopoietic prostaglandin D synthase
ENSG00000163138	PACRGL	parkin coregulated like
ENSG00000163161	ERCC3	ERCC excision repair 3, TFIIH core complex helicase subunit
ENSG00000163166	IWS1	interacts with SUPT6H, CTD assembly factor 1
ENSG00000163288	GABRB1	gamma-aminobutyric acid type A receptor subunit beta1
ENSG00000163297	ANTXR2	ANTXR cell adhesion molecule 2
ENSG00000163406	SLC15A2	solute carrier family 15 member 2
ENSG00000163430	FSTL1	follistatin like 1
ENSG00000163492	CCDC141	coiled-coil domain containing 141
ENSG00000163507	CIP2A	cellular inhibitor of PP2A
ENSG00000163534	FCRL1	Fc receptor like 1
ENSG00000163539	CLASP2	cytoplasmic linker associated protein 2
ENSG00000163576	EFHB	EF-hand domain family member B
ENSG00000163577	EIF5A2	eukaryotic translation initiation factor 5A2
ENSG00000163605	PPP4R2	protein phosphatase 4 regulatory subunit 2
ENSG00000163629	PTPN13	protein tyrosine phosphatase non-receptor type 13
ENSG00000163644	PPM1K	protein phosphatase, Mg2+/Mn2+ dependent 1K
ENSG00000163661	PTX3	pentraxin 3
ENSG00000163714	U2SURP	U2 snRNP associated SURP domain containing
ENSG00000163736	PPBP	pro-platelet basic protein
ENSG00000163738	MTHFD2L	methylenetetrahydrofolate dehydrogenase (NADP+ dependent) 2 like

ENSG00000163739	CXCL1	C-X-C motif chemokine ligand 1
ENSG00000163743	RCHY1	ring finger and CHY zinc finger domain containing 1
ENSG00000163874	ZC3H12A	zinc finger CCCH-type containing 12A
ENSG00000163923	RPL39L	ribosomal protein L39 like
ENSG00000163933	RFT1	RFT1 homolog
ENSG00000163964	PIGX	phosphatidylinositol glycan anchor biosynthesis class X
ENSG00000164037	SLC9B1	solute carrier family 9 member B1
ENSG00000164038	SLC9B2	solute carrier family 9 member B2
ENSG00000164091	WDR82	WD repeat domain 82
ENSG00000164118	CEP44	centrosomal protein 44
ENSG00000164120	HPGD	15-hydroxyprostaglandin dehydrogenase
ENSG00000164134	NAA15	N-alpha-acetyltransferase 15, NatA auxiliary subunit
ENSG00000164180	TMEM161B	transmembrane protein 161B
ENSG00000164187	LMBRD2	LMBR1 domain containing 2
ENSG00000164209	SLC25A46	solute carrier family 25 member 46
ENSG00000164219	PGGT1B	protein geranylgeranyltransferase type I subunit beta
ENSG00000164241	C5orf63	chromosome 5 open reading frame 63
ENSG00000164330	EBF1	EBF transcription factor 1
ENSG00000164346	NSA2	NSA2 ribosome biogenesis factor
ENSG00000164404	GDF9	growth differentiation factor 9
ENSG00000164405	UQCRQ	ubiquinol-cytochrome c reductase complex III subunit VII
ENSG00000164414	SLC35A1	solute carrier family 35 member A1
ENSG00000164434	FABP7	fatty acid binding protein 7
ENSG00000164494	PDSS2	decaprenyl diphosphate synthase subunit 2
ENSG00000164532	TBX20	T-box transcription factor 20
ENSG00000164543	STK17A	serine/threonine kinase 17a
ENSG00000164548	TRA2A	transformer 2 alpha homolog
ENSG00000164649	CDCA7L	cell division cycle associated 7 like
ENSG00000164761	TNFRSF11B	TNF receptor superfamily member 11b
ENSG00000164823	OSGIN2	oxidative stress induced growth inhibitor family member 2
ENSG00000164929	BAALC	BAALC binder of MAP3K1 and KLF4
ENSG00000164944	VIRMA	vir like m6A methyltransferase associated
ENSG00000164953	TMEM67	transmembrane protein 67
ENSG00000164983	TMEM65	transmembrane protein 65
ENSG00000164989	CCDC171	coiled-coil domain containing 171
ENSG00000165113	GKAP1	G kinase anchoring protein 1
ENSG00000165115	KIF27	kinesin family member 27
ENSG00000165209	STRBP	spermatid perinuclear RNA binding protein
ENSG00000165219	GAPVD1	GTPase activating protein and VPS9 domains 1
ENSG00000165259	HDX	highly divergent homeobox
ENSG00000165264	NDUFB6	NADH:ubiquinone oxidoreductase subunit B6
ENSG00000165309	ARMC3	armadillo repeat containing 3
ENSG00000165322	ARHGAP12	Rho GTPase activating protein 12

ENSG00000165338	HECTD2	HECT domain E3 ubiquitin protein ligase 2
ENSG00000165392	WRN	WRN RecQ like helicase
ENSG00000165416	SUGT1	SGT1 homolog, MIS12 kinetochore complex assembly cochaperone
ENSG00000165474	GJB2	gap junction protein beta 2
ENSG00000165494	PCF11	PCF11 cleavage and polyadenylation factor subunit
ENSG00000165521	EML5	EMAP like 5
ENSG00000165591	FAAH2	fatty acid amide hydrolase 2
ENSG00000165630	PRPF18	pre-mRNA processing factor 18
ENSG00000165661	QSOX2	quiescin sulfhydryl oxidase 2
ENSG00000165669	FAM204A	family with sequence similarity 204 member A
ENSG00000165813	CCDC186	coiled-coil domain containing 186
ENSG00000165923	AGBL2	ATP/GTP binding protein like 2
ENSG00000165929	TC2N	tandem C2 domains, nuclear
ENSG00000166024	R3HCC1L	R3H domain and coiled-coil containing 1 like
ENSG00000166135	HIF1AN	hypoxia inducible factor 1 subunit alpha inhibitor
ENSG00000166147	FBN1	fibrillin 1
ENSG00000166153	DEPDC4	DEP domain containing 4
ENSG00000166167	BTRC	beta-transducin repeat containing E3 ubiquitin protein ligase
ENSG00000166225	FRS2	fibroblast growth factor receptor substrate 2
ENSG00000166262	FAM227B	family with sequence similarity 227 member B
ENSG00000166263	STXBP4	syntaxin binding protein 4
ENSG00000166323	C11orf65	chromosome 11 open reading frame 65
ENSG00000166377	ATP9B	ATPase phospholipid transporting 9B (putative)
ENSG00000166396	SERPINB7	serpin family B member 7
ENSG00000166435	XRRA1	X-ray radiation resistance associated 1
ENSG00000166439	RNF169	ring finger protein 169
ENSG00000166479	TMX3	thioredoxin related transmembrane protein 3
ENSG00000166575	TMEM135	transmembrane protein 135
ENSG00000166582	CENPV	centromere protein V
ENSG00000166603	MC4R	melanocortin 4 receptor
ENSG00000166750	SLFN5	schlafen family member 5
ENSG00000166788	SAAL1	serum amyloid A like 1
ENSG00000166845	C18orf54	chromosome 18 open reading frame 54
ENSG00000166920	C15orf48	chromosome 15 open reading frame 48
ENSG00000167202	TBC1D2B	TBC1 domain family member 2B
ENSG00000167216	KATNAL2	katanin catalytic subunit A1 like 2
ENSG00000167220	HDHD2	haloacid dehalogenase like hydrolase domain containing 2
ENSG00000167232	ZNF91	zinc finger protein 91
ENSG00000167325	RRM1	ribonucleotide reductase catalytic subunit M1
ENSG00000168038	ULK4	unc-51 like kinase 4
ENSG00000168234	TTC39C	tetratricopeptide repeat domain 39C
ENSG00000168333	PPDPFL	pancreatic progenitor cell differentiation and proliferation factor like
ENSG00000168389	MFSD2A	major facilitator superfamily domain containing 2A

ENSG00000168438	CDC40	cell division cycle 40
ENSG00000168939	SPRY3	sprouty RTK signaling antagonist 3
ENSG00000169020	ATP5ME	ATP synthase membrane subunit e
ENSG00000169045	HNRNPH1	heterogeneous nuclear ribonucleoprotein H1
ENSG00000169062	UPF3A	UPF3A regulator of nonsense mediated mRNA decay
ENSG00000169139	UBE2V2	ubiquitin conjugating enzyme E2 V2
ENSG00000169288	MRPL1	mitochondrial ribosomal protein L1
ENSG00000169306	IL1RAPL1	interleukin 1 receptor accessory protein like 1
ENSG00000169372	CRADD	CASP2 and RIPK1 domain containing adaptor with death domain
ENSG00000169429	CXCL8	C-X-C motif chemokine ligand 8
ENSG00000169507	SLC38A11	solute carrier family 38 member 11
ENSG00000169519	METTL15	methyltransferase like 15
ENSG00000169621	APLF	aprataxin and PNKP like factor
ENSG00000169679	BUB1	BUB1 mitotic checkpoint serine/threonine kinase
ENSG00000169764	UGP2	UDP-glucose pyrophosphorylase 2
ENSG00000169914	OTUD3	OTU deubiquitinase 3
ENSG00000169976	SF3B5	splicing factor 3b subunit 5
ENSG00000170085	SIMC1	SUMO interacting motifs containing 1
ENSG00000170144	HNRNPA3	heterogeneous nuclear ribonucleoprotein A3
ENSG00000170242	USP47	ubiquitin specific peptidase 47
ENSG00000170264	FAM161A	FAM161 centrosomal protein A
ENSG00000170312	CDK1	cyclin dependent kinase 1
ENSG00000170340	B3GNT2	UDP-GlcNAc:betaGal beta-1,3-N-acetylglucosaminyltransferase 2
ENSG00000170379	TCAF2	TRPM8 channel associated factor 2
ENSG00000170417	TMEM182	transmembrane protein 182
ENSG00000170537	TMC7	transmembrane channel like 7
ENSG00000170542	SERPINB9	serpin family B member 9
ENSG00000170571	EMB	embigin
ENSG00000170632	ARMC10	armadillo repeat containing 10
ENSG00000170681	CAVIN4	caveolae associated protein 4
ENSG00000170832	USP32	ubiquitin specific peptidase 32
ENSG00000170852	KBTBD2	kelch repeat and BTB domain containing 2
ENSG00000170899	GSTA4	glutathione S-transferase alpha 4
ENSG00000170903	MSANTD4	Myb/SANT DNA binding domain containing 4 with coiled-coils
ENSG00000170946	DNAJC24	DnaJ heat shock protein family (Hsp40) member C24
ENSG00000171044	XKR6	XK related 6
ENSG00000171049	FPR2	formyl peptide receptor 2
ENSG00000171241	SHCBP1	SHC binding and spindle associated 1
ENSG00000171316	CHD7	chromodomain helicase DNA binding protein 7
ENSG00000171503	ETFDH	electron transfer flavoprotein dehydrogenase
ENSG00000171681	ATF7IP	activating transcription factor 7 interacting protein
ENSG00000171931	FBXW10	F-box and WD repeat domain containing 10
ENSG00000171960	PPIH	peptidylprolyl isomerase H

ENSG00000172167	MTBP	MDM2 binding protein
ENSG00000172171	TEFM	transcription elongation factor, mitochondrial
ENSG00000172244	C5orf34	chromosome 5 open reading frame 34
ENSG00000172339	ALG14	ALG14 UDP-N-acetylglucosaminyltransferase subunit
ENSG00000172404	DNAJB7	DnaJ heat shock protein family (Hsp40) member B7
ENSG00000172456	FGGY	FGGY carbohydrate kinase domain containing
ENSG00000172586	CHCHD1	coiled-coil-helix-coiled-coil-helix domain containing 1
ENSG00000172594	SMPDL3A	sphingomyelin phosphodiesterase acid like 3A
ENSG00000172728	FUT10	fucosyltransferase 10
ENSG00000172748	ZNF596	zinc finger protein 596
ENSG00000172817	CYP7B1	cytochrome P450 family 7 subfamily B member 1
ENSG00000172915	NBEA	neurobeachin
ENSG00000173041	ZNF680	zinc finger protein 680
ENSG00000173064	HECTD4	HECT domain E3 ubiquitin protein ligase 4
ENSG00000173085	COQ2	coenzyme Q2, polyprenyltransferase
ENSG00000173226	IQCB1	IQ motif containing B1
ENSG00000173230	GOLGB1	golgin B1
ENSG00000173258	ZNF483	zinc finger protein 483
ENSG00000173273	TNKS	tankyrase
ENSG00000173418	NAA20	N-alpha-acetyltransferase 20, NatB catalytic subunit
ENSG00000173473	SMARCC1	SWI/SNF related, matrix associated, actin dependent regulator of chromatin subfamily c member 1
ENSG00000173597	SULT1B1	sulfotransferase family 1B member 1
ENSG00000173611	SCAI	suppressor of cancer cell invasion
ENSG00000173681	BCLAF3	BCLAF1 and THRAP3 family member 3
ENSG00000173715	C11orf80	chromosome 11 open reading frame 80
ENSG00000173821	RNF213	ring finger protein 213
ENSG00000173862		novel transcript
ENSG00000173890	GPR160	G protein-coupled receptor 160
ENSG00000174007	CEP19	centrosomal protein 19
ENSG00000174132	FAM174A	family with sequence similarity 174 member A
ENSG00000174373	RALGAPA1	Ral GTPase activating protein catalytic subunit alpha 1
ENSG00000174485	DENND4A	DENN domain containing 4A
ENSG00000174501	ANKRD36C	ankyrin repeat domain 36C
ENSG00000174529	TMEM81	transmembrane protein 81
ENSG00000174579	MSL2	MSL complex subunit 2
ENSG00000174720	LARP7	La ribonucleoprotein 7, transcriptional regulator
ENSG00000174796	THAP6	THAP domain containing 6
ENSG00000174799	CEP135	centrosomal protein 135
ENSG00000174837	ADGRE1	adhesion G protein-coupled receptor E1
ENSG00000174842	GLMN	glomulin, FKBP associated protein
ENSG00000174953	DHX36	DEAH-box helicase 36
ENSG00000174989	FBXW8	F-box and WD repeat domain containing 8

ENSG00000175003	SLC22A1	solute carrier family 22 member 1
ENSG00000175054	ATR	ATR serine/threonine kinase
ENSG00000175216	CKAP5	cytoskeleton associated protein 5
ENSG00000175322	ZNF519	zinc finger protein 519
ENSG00000175354	PTPN2	protein tyrosine phosphatase non-receptor type 2
ENSG00000175455	CCDC14	coiled-coil domain containing 14
ENSG00000175600	SUGCT	succinyl-CoA:glutarate-CoA transferase
ENSG00000175611	LINC00476	long intergenic non-protein coding RNA 476
ENSG00000175746	C15orf54	chromosome 15 putative open reading frame 54
ENSG00000175841	FAM172BP	family with sequence similarity 172 member B, pseudogene
ENSG00000175893	ZDHHC21	zinc finger DHHC-type palmitoyltransferase 21
ENSG00000175984	DENND2C	DENN domain containing 2C
ENSG00000176208	ATAD5	ATPase family AAA domain containing 5
ENSG00000176222	ZNF404	zinc finger protein 404
ENSG00000176225	RTTN	rotatin
ENSG00000176293	ZNF135	zinc finger protein 135
ENSG00000176294	OR4N2	olfactory receptor family 4 subfamily N member 2
ENSG00000176542	USF3	upstream transcription factor family member 3
ENSG00000176658	MYO1D	myosin ID
ENSG00000176809	LRRC37A3	leucine rich repeat containing 37 member A3
ENSG00000176896	TCEANC	transcription elongation factor A N-terminal and central domain containing
ENSG00000176907	TCIM	transcriptional and immune response regulator
ENSG00000176970	RPL7L1P11	ribosomal protein L7 like 1 pseudogene 11
ENSG00000177200	CHD9	chromodomain helicase DNA binding protein 9
ENSG00000177272	KCNA3	potassium voltage-gated channel subfamily A member 3
ENSG00000177565	TBL1XR1	TBL1X receptor 1
ENSG00000177570	SAMD12	sterile alpha motif domain containing 12
ENSG00000177602	HASPIN	histone H3 associated protein kinase
ENSG00000177738		novel transcript, sense overlapping to ANXA2R
ENSG00000177842	ZNF620	zinc finger protein 620
ENSG00000177853	ZNF518A	zinc finger protein 518A
ENSG00000178028	DMAP1	DNA methyltransferase 1 associated protein 1
ENSG00000178033	CALHM5	calcium homeostasis modulator family member 5
ENSG00000178104	PDE4DIP	phosphodiesterase 4D interacting protein
ENSG00000178163	ZNF518B	zinc finger protein 518B
ENSG00000178172	SPINK6	serine peptidase inhibitor Kazal type 6
ENSG00000178202	POGLUT3	protein O-glucosyltransferase 3
ENSG00000178295	GEN1	GEN1 Holliday junction 5' flap endonuclease
ENSG00000178449	COX14	cytochrome c oxidase assembly factor COX14
ENSG00000178502	KLHL11	kelch like family member 11
ENSG00000178852	EFCAB13	EF-hand calcium binding domain 13
ENSG00000178904	DPY19L3	dpy-19 like C-mannosyltransferase 3

ENSG00000178974	FBXO34	F-box protein 34
ENSG00000179071	CCDC89	coiled-coil domain containing 89
ENSG00000179104	TMTC2	transmembrane O-mannosyltransferase targeting cadherins 2
ENSG00000179362	HMGN2P46	high mobility group nucleosomal binding domain 2 pseudogene 46
ENSG00000179363	TMEM31	transmembrane protein 31
ENSG00000179387	ELMOD2	ELMO domain containing 2
ENSG00000179397	CATSPERE	catsper channel auxiliary subunit epsilon
ENSG00000179428	IL6-AS1	IL6 antisense RNA 1
ENSG00000179468	OR9A2	olfactory receptor family 9 subfamily A member 2
ENSG00000179676	LINC00305	long intergenic non-protein coding RNA 305
ENSG00000180116	C12orf40	chromosome 12 open reading frame 40
ENSG00000180245	RRH	retinal pigment epithelium-derived rhodopsin homolog
ENSG00000180316	PNPLA1	patatin like phospholipase domain containing 1
ENSG00000180385	EMC3-AS1	EMC3 antisense RNA 1
ENSG00000180481	GLIPR1L2	GLIPR1 like 2
ENSG00000180488	MIGA1	mitoguardin 1
ENSG00000180770	OR7E129P	olfactory receptor family 7 subfamily E member 129 pseudogene
ENSG00000180801	ARSJ	arylsulfatase family member J
ENSG00000180881	CAPS2	calcyphosine 2
ENSG00000180957	PITPNB	phosphatidylinositol transfer protein beta
ENSG00000181090	EHMT1	euchromatic histone lysine methyltransferase 1
ENSG00000181544	FANCB	FA complementation group B
ENSG00000181619	GPR135	G protein-coupled receptor 135
ENSG00000181722	ZBTB20	zinc finger and BTB domain containing 20
ENSG00000181744	DIPK2A	divergent protein kinase domain 2A
ENSG00000181751	MACIR	macrophage immunometabolism regulator
ENSG00000181785	OR5AS1	olfactory receptor family 5 subfamily AS member 1
ENSG00000181804	SLC9A9	solute carrier family 9 member A9
ENSG00000181938	GINS3	GINS complex subunit 3
ENSG00000182004	SNRPE	small nuclear ribonucleoprotein polypeptide E
ENSG00000182010	RTKN2	rhotekin 2
ENSG00000182185	RAD51B	RAD51 paralog B
ENSG00000182359	KBTBD3	kelch repeat and BTB domain containing 3
ENSG00000182365	OR5F2P	olfactory receptor family 5 subfamily F member 2 pseudogene
ENSG00000182504	CEP97	centrosomal protein 97
ENSG00000182541	LIMK2	LIM domain kinase 2
ENSG00000182628	SKA2	spindle and kinetochore associated complex subunit 2
ENSG00000182648	LINC01006	long intergenic non-protein coding RNA 1006
ENSG00000182670	TTC3	tetratricopeptide repeat domain 3
ENSG00000182782	HCAR2	hydroxycarboxylic acid receptor 2
ENSG00000182993	C12orf60	chromosome 12 open reading frame 60
ENSG00000183066	WBP2NL	WBP2 N-terminal like
ENSG00000183150	GPR19	G protein-coupled receptor 19

ENSG00000183340	JRKL	JRK like
ENSG00000183432	ZBTB8OSP1	zinc finger and BTB domain containing 8 opposite strand pseudogene 1
ENSG00000183718	TRIM52	tripartite motif containing 52
ENSG00000183742	MACC1	MET transcriptional regulator MACC1
ENSG00000183814	LIN9	lin-9 DREAM MuvB core complex component
ENSG00000183826	BTBD9	BTB domain containing 9
ENSG00000183891	TTC32	tetratricopeptide repeat domain 32
ENSG00000184005	ST6GALNAC3	ST6 N-acetylgalactosaminide alpha-2,6-sialyltransferase 3
ENSG00000184014	DENND5A	DENN domain containing 5A
ENSG00000184022	OR2T10	olfactory receptor family 2 subfamily T member 10
ENSG00000184055	OR7E87P	olfactory receptor family 7 subfamily E member 87 pseudogene
ENSG00000184178	SCFD2	sec1 family domain containing 2
ENSG00000184208	C22orf46	chromosome 22 open reading frame 46
ENSG00000184209	SNRNP35	small nuclear ribonucleoprotein U11/U12 subunit 35
ENSG00000184277	TM2D3	TM2 domain containing 3
ENSG00000184321	OR51J1	olfactory receptor family 51 subfamily J member 1
ENSG00000184394	OR4N5	olfactory receptor family 4 subfamily N member 5
ENSG00000184428	TOP1MT	DNA topoisomerase I mitochondrial
ENSG00000184432	COPB2	COPI coat complex subunit beta 2
ENSG00000184445	KNTC1	kinetochore associated 1
ENSG00000184557	SOCS3	suppressor of cytokine signaling 3
ENSG00000184661	CDCA2	cell division cycle associated 2
ENSG00000184787	UBE2G2	ubiquitin conjugating enzyme E2 G2
ENSG00000184838	PRR16	proline rich 16
ENSG00000184860	SDR42E1	short chain dehydrogenase/reductase family 42E, member 1
ENSG00000184903	IMMP2L	inner mitochondrial membrane peptidase subunit 2
ENSG00000185015	CA13	carbonic anhydrase 13
ENSG00000185104	FAF1	Fas associated factor 1
ENSG00000185246	PRPF39	pre-mRNA processing factor 39
ENSG00000185261	KIAA0825	KIAA0825
ENSG00000185404	SP140L	SP140 nuclear body protein like
ENSG00000185418	TARS3	threonyl-tRNA synthetase 3
ENSG00000185420	SMYD3	SET and MYND domain containing 3
ENSG00000185480	PARPBP	PARP1 binding protein
ENSG00000185658	BRWD1	bromodomain and WD repeat domain containing 1
ENSG00000185716	MOSMO	modulator of smoothened
ENSG00000185760	KCNQ5	potassium voltage-gated channel subfamily Q member 5
ENSG00000185917	SETD4	SET domain containing 4
ENSG00000186020	ZNF529	zinc finger protein 529
ENSG00000186119	OR5D18	olfactory receptor family 5 subfamily D member 18
ENSG00000186146	DEFB131A	defensin beta 131A
ENSG00000186187	ZNRF1	zinc and ring finger 1

ENSG00000186409	CCDC30	coiled-coil domain containing 30
ENSG00000186625	KATNA1	katanin catalytic subunit A1
ENSG00000186803	IFNA10	interferon alpha 10
ENSG00000186812	ZNF397	zinc finger protein 397
ENSG00000186908	ZDHHC17	zinc finger DHHC-type palmitoyltransferase 17
ENSG00000186952	TMEM232	transmembrane protein 232
ENSG00000187118	CMC1	C-X9-C motif containing 1
ENSG00000187210	GCNT1	glucosaminyl (N-acetyl) transferase 1
ENSG00000187240	DYNC2H1	dynein cytoplasmic 2 heavy chain 1
ENSG00000187555	USP7	ubiquitin specific peptidase 7
ENSG00000187559	FOXD4L3	forkhead box D4 like 3
ENSG00000187753	C9orf153	chromosome 9 open reading frame 153
ENSG00000187790	FANCM	FA complementation group M
ENSG00000187801	ZFP69B	ZFP69 zinc finger protein B
ENSG00000188107	EYS	eyes shut homolog
ENSG00000188175	HEPACAM2	HEPACAM family member 2
ENSG00000188177	ZC3H6	zinc finger CCCH-type containing 6
ENSG00000188227	ZNF793	zinc finger protein 793
ENSG00000188342	GTF2F2	general transcription factor IIF subunit 2
ENSG00000188352	FOCAD	focadhesin
ENSG00000188529	SRSF10	serine and arginine rich splicing factor 10
ENSG00000188558	OR2G6	olfactory receptor family 2 subfamily G member 6
ENSG00000188612	SUMO2	small ubiquitin like modifier 2
ENSG00000188641	DPYD	dihydropyrimidine dehydrogenase
ENSG00000188647	PTAR1	protein prenyltransferase alpha subunit repeat containing 1
ENSG00000188725	SMIM15	small integral membrane protein 15
ENSG00000188800	TMCO2	transmembrane and coiled-coil domains 2
ENSG00000188993	LRRC66	leucine rich repeat containing 66
ENSG00000188994	ZNF292	zinc finger protein 292
ENSG00000189079	ARID2	AT-rich interaction domain 2
ENSG00000189144	ZNF573	zinc finger protein 573
ENSG00000189269	DRICH1	aspartate rich 1
ENSG00000196074	SYCP2	synaptonemal complex protein 2
ENSG00000196083	IL1RAP	interleukin 1 receptor accessory protein
ENSG00000196151	WDSUB1	WD repeat, sterile alpha motif and U-box domain containing 1
ENSG00000196177	ACADSB	acyl-CoA dehydrogenase short/branched chain
ENSG00000196199	MPHOSPH8	M-phase phosphoprotein 8
ENSG00000196233	LCOR	ligand dependent nuclear receptor corepressor
ENSG00000196263	ZNF471	zinc finger protein 471
ENSG00000196284	SUPT3H	SPT3 homolog, SAGA and STAGA complex component
ENSG00000196383	OR4Q2	olfactory receptor family 4 subfamily Q member 2 (gene/pseudogene)
ENSG00000196418	ZNF124	zinc finger protein 124

ENSG00000196505	GDAP2	ganglioside induced differentiation associated protein 2
ENSG00000196511	TPK1	thiamin pyrophosphokinase 1
ENSG00000196584	XRCC2	X-ray repair cross complementing 2
ENSG00000196597	ZNF782	zinc finger protein 782
ENSG00000196628	TCF4	transcription factor 4
ENSG00000196693	ZNF33B	zinc finger protein 33B
ENSG00000196730	DAPK1	death associated protein kinase 1
ENSG00000196792	STRN3	striatin 3
ENSG00000196865	NHLRC2	NHL repeat containing 2
ENSG00000196867	ZFP28	ZFP28 zinc finger protein
ENSG00000196922	ZNF252P	zinc finger protein 252, pseudogene
ENSG00000196932	TMEM26	transmembrane protein 26
ENSG00000196935	SRGAP1	SLIT-ROBO Rho GTPase activating protein 1
ENSG00000196950	SLC39A10	solute carrier family 39 member 10
ENSG00000197008	ZNF138	zinc finger protein 138
ENSG00000197021	EOLA2	endothelium and lymphocyte associated ASCH domain 2
ENSG00000197037	ZSCAN25	zinc finger and SCAN domain containing 25
ENSG00000197043	ANXA6	annexin A6
ENSG00000197102	DYNC1H1	dynein cytoplasmic 1 heavy chain 1
ENSG00000197124	ZNF682	zinc finger protein 682
ENSG00000197275	RAD54B	RAD54 homolog B
ENSG00000197299	BLM	BLM RecQ like helicase
ENSG00000197302	ZNF720	zinc finger protein 720
ENSG00000197415	VEPH1	ventricular zone expressed PH domain containing 1
ENSG00000197497	ZNF665	zinc finger protein 665
ENSG00000197548	ATG7	autophagy related 7
ENSG00000197635	DPP4	dipeptidyl peptidase 4
ENSG00000197694	SPTAN1	spectrin alpha, non-erythrocytic 1
ENSG00000197713	RPE	ribulose-5-phosphate-3-epimerase
ENSG00000197714	ZNF460	zinc finger protein 460
ENSG00000197779	ZNF81	zinc finger protein 81
ENSG00000197808	ZNF461	zinc finger protein 461
ENSG00000197892	KIF13B	kinesin family member 13B
ENSG00000197937	ZNF347	zinc finger protein 347
ENSG00000197959	DNM3	dynamin 3
ENSG00000197965	MPZL1	myelin protein zero like 1
ENSG00000197969	VPS13A	vacuolar protein sorting 13 homolog A
ENSG00000197980	LEKR1	leucine, glutamate and lysine rich 1
ENSG00000198046	ZNF667	zinc finger protein 667
ENSG00000198060	MARCHF5	membrane associated ring-CH-type finger 5
ENSG00000198088	NUP62CL	nucleoporin 62 C-terminal like
ENSG00000198105	ZNF248	zinc finger protein 248
ENSG00000198128	OR2L3	olfactory receptor family 2 subfamily L member 3

ENSG00000198130	HIBCH	3-hydroxyisobutyryl-CoA hydrolase
ENSG00000198146	ZNF770	zinc finger protein 770
ENSG00000198160	MIER1	MIER1 transcriptional regulator
ENSG00000198169	ZNF251	zinc finger protein 251
ENSG00000198258	UBL5	ubiquitin like 5
ENSG00000198283	OR5B21	olfactory receptor family 5 subfamily B member 21
ENSG00000198393	ZNF26	zinc finger protein 26
ENSG00000198399	ITSN2	intersectin 2
ENSG00000198416	ZNF658B	zinc finger protein 658B (pseudogene)
ENSG00000198453	ZNF568	zinc finger protein 568
ENSG00000198498	TMA16	translation machinery associated 16 homolog
ENSG00000198521	ZNF43	zinc finger protein 43
ENSG00000198554	WDHD1	WD repeat and HMG-box DNA binding protein 1
ENSG00000198589	LRBA	LPS responsive beige-like anchor protein
ENSG00000198590	C3orf35	chromosome 3 open reading frame 35
ENSG00000198624	CCDC69	coiled-coil domain containing 69
ENSG00000198625	MDM4	MDM4 regulator of p53
ENSG00000198707	CEP290	centrosomal protein 290
ENSG00000198734	F5	coagulation factor V
ENSG00000198826	ARHGAP11A	Rho GTPase activating protein 11A
ENSG00000198836	OPA1	OPA1 mitochondrial dynamin like GTPase
ENSG00000198887	SMC5	structural maintenance of chromosomes 5
ENSG00000198890	PRMT6	protein arginine methyltransferase 6
ENSG00000198920	KIAA0753	KIAA0753
ENSG00000198924	DCLRE1A	DNA cross-link repair 1A
ENSG00000198947	DMD	dystrophin
ENSG00000199135	MIR101-1	microRNA 101-1
ENSG00000199153	MIR30D	microRNA 30d
ENSG00000199172	MIR331	microRNA 331
ENSG00000199212	RNU105C	RNA, U105C small nucleolar
ENSG00000199246	RNU6-896P	RNA, U6 small nuclear 896, pseudogene
ENSG00000199283	RNU1-58P	RNA, U1 small nuclear 58, pseudogene
ENSG00000199289	RNU6-502P	RNA, U6 small nuclear 502, pseudogene
ENSG00000199332		Y RNA [Source:RFAM;Acc:RF00019]
ENSG00000199405	SNORA1B	small nucleolar RNA, H/ACA box 1B
ENSG00000199483	RNU4-15P	RNA, U4 small nuclear 15, pseudogene
ENSG00000199550		Y RNA [Source:RFAM;Acc:RF00019]
ENSG00000199567		Y RNA [Source:RFAM;Acc:RF00019]
ENSG00000199698		Y RNA [Source:RFAM;Acc:RF00019]
ENSG00000199728	RNU6-655P	RNA, U6 small nuclear 655, pseudogene
ENSG00000199740		Y RNA [Source:RFAM;Acc:RF00019]
ENSG00000199840		Y RNA [Source:RFAM;Acc:RF00019]
ENSG00000199990	VTRNA1-1	vault RNA 1-1

ENSG00000200060		Y RNA [Source:RFAM;Acc:RF00019]
ENSG00000200325		Y RNA [Source:RFAM;Acc:RF00019]
ENSG00000200327	RNA5SP62	RNA, 5S ribosomal pseudogene 62
ENSG00000200485		Y RNA [Source:RFAM;Acc:RF00019]
ENSG00000200719	RNA5SP260	RNA, 5S ribosomal pseudogene 260
ENSG00000200885	RNU1-146P	RNA, U1 small nuclear 146, pseudogene
ENSG00000200890	RNA5SP99	RNA, 5S ribosomal pseudogene 99
ENSG00000200898	RNU6-1243P	RNA, U6 small nuclear 1243, pseudogene
ENSG00000200991		
ENSG00000201041	RNA5SP242	RNA, 5S ribosomal pseudogene 242
ENSG00000201088		Y RNA [Source:RFAM;Acc:RF00019]
ENSG00000201113	RNU6-647P	RNA, U6 small nuclear 647, pseudogene
ENSG00000201133		
ENSG00000201136	RNU6-353P	RNA, U6 small nuclear 353, pseudogene
ENSG00000201179	RNU6-1322P	RNA, U6 small nuclear 1322, pseudogene
ENSG00000201243	RNU6-654P	RNA, U6 small nuclear 654, pseudogene
ENSG00000201370		Y RNA [Source:RFAM;Acc:RF00019]
ENSG00000201371		Y RNA [Source:RFAM;Acc:RF00019]
ENSG00000201431	RNU6-1277P	RNA, U6 small nuclear 1277, pseudogene
ENSG00000201545	RNU4-85P	RNA, U4 small nuclear 85, pseudogene
ENSG00000201628	RNU4-7P	RNA, U4 small nuclear 7, pseudogene
ENSG00000201701		
ENSG00000201786		Y RNA [Source:RFAM;Acc:RF00019]
ENSG00000201852	RNU6-702P	RNA, U6 small nuclear 702, pseudogene
ENSG00000201881		Y RNA [Source:RFAM;Acc:RF00019]
ENSG00000201939	RNA5SP224	RNA, 5S ribosomal pseudogene 224
ENSG00000202175	RNA5SP128	RNA, 5S ribosomal pseudogene 128
ENSG00000202251		Y RNA [Source:RFAM;Acc:RF00019]
ENSG00000202272		Y RNA [Source:RFAM;Acc:RF00019]
ENSG00000202399		Y RNA [Source:RFAM;Acc:RF00019]
ENSG00000202515	VTRNA1-3	vault RNA 1-3
ENSG00000203721	LINC00862	long intergenic non-protein coding RNA 862
ENSG00000203739	PRDX6-AS1	PRDX6 antisense RNA 1
ENSG00000203804	ADAMTSL4-AS1	ADAMTSL4 antisense RNA 1
ENSG00000203965	EFCAB7	EF-hand calcium binding domain 7
ENSG00000204130	RUFY2	RUN and FYVE domain containing 2
ENSG00000204246	OR13C3	olfactory receptor family 13 subfamily C member 3
ENSG00000204406	MBD5	methyl-CpG binding domain protein 5
ENSG00000204524	ZNF805	zinc finger protein 805
ENSG00000205213	LGR4	leucine rich repeat containing G protein-coupled receptor 4
ENSG00000205268	PDE7A	phosphodiesterase 7A
ENSG00000205364	MT1M	metallothionein 1M

ENSG00000205531	NAP1L4	nucleosome assembly protein 1 like 4
ENSG00000205930	C21orf62-AS1	C21orf62 antisense RNA 1
ENSG00000206530	CFAP44	cilia and flagella associated protein 44
ENSG00000206573	THUMPD3-AS1	THUMPD3 antisense RNA 1
ENSG00000206679		Y RNA [Source:RFAM;Acc:RF00019]
ENSG00000206708	RNU6-1227P	RNA, U6 small nuclear 1227, pseudogene
ENSG00000206731		
ENSG00000206738		Y RNA [Source:RFAM;Acc:RF00019]
ENSG00000206808		Y RNA [Source:RFAM;Acc:RF00019]
ENSG00000206815	RNU6-483P	RNA, U6 small nuclear 483, pseudogene
ENSG00000206822		Y RNA [Source:RFAM;Acc:RF00019]
ENSG00000206841	RNU6-409P	RNA, U6 small nuclear 409, pseudogene
ENSG00000206871	RNU6-533P	RNA, U6 small nuclear 533, pseudogene
ENSG00000206878		
ENSG00000206907	RNU6-1013P	RNA, U6 small nuclear 1013, pseudogene
ENSG00000206935	RNU6-514P	RNA, U6 small nuclear 514, pseudogene
ENSG00000207033	RNU6-154P	RNA, U6 small nuclear 154, pseudogene
ENSG00000207090	RNU6-517P	RNA, U6 small nuclear 517, pseudogene
ENSG00000207153	RNU6-933P	RNA, U6 small nuclear 933, pseudogene
ENSG00000207203	RNU6-71P	RNA, U6 small nuclear 71, pseudogene
ENSG00000207252		Y RNA [Source:RFAM;Acc:RF00019]
ENSG00000207292		Y RNA [Source:RFAM;Acc:RF00019]
ENSG00000207312	RNU6-429P	RNA, U6 small nuclear 429, pseudogene
ENSG00000207329		Y RNA [Source:RFAM;Acc:RF00019]
ENSG00000207356		Y RNA [Source:RFAM;Acc:RF00019]
ENSG00000207359	RNU6-925P	RNA, U6 small nuclear 925, pseudogene
ENSG00000207452	RNU6-606P	RNA, U6 small nuclear 606, pseudogene
ENSG00000207502		
ENSG00000207946	MIR516B1	microRNA 516b-1
ENSG00000207948	MIR328	microRNA 328
ENSG00000210082	MT-RNR2	mitochondrially encoded 16S rRNA
ENSG00000210181	RNU6ATAC4P	RNA, U6atac small nuclear 4, pseudogene
ENSG00000211456	SACM1L	SAC1 like phosphatidylinositide phosphatase
ENSG00000211460	TSN	translin
ENSG00000211699	TRGV3	T cell receptor gamma variable 3
ENSG00000212289	RNA5SP339	RNA, 5S ribosomal pseudogene 339
ENSG00000212319		Y RNA [Source:RFAM;Acc:RF00019]
ENSG00000212425	RNA5SP105	RNA, 5S ribosomal pseudogene 105
ENSG00000212518	RNU11-5P	RNA, U11 small nuclear 5, pseudogene
ENSG00000212527	RNA5SP63	RNA, 5S ribosomal pseudogene 63
ENSG00000212589		
ENSG00000212628	RNA5SP241	RNA, 5S ribosomal pseudogene 241
ENSG00000213079	SCAF8	SR-related CTD associated factor 8

ENSG00000213096	ZNF254	zinc finger protein 254
ENSG00000213139	CRYGS	crystallin gamma S
ENSG00000213160	KLHL23	kelch like family member 23
ENSG00000213203	GIMAP1	GTPase, IMAP family member 1
ENSG00000213707	HMGB1P10	high mobility group box 1 pseudogene 10
ENSG00000213801	ZNF321P	zinc finger protein 321, pseudogene
ENSG00000213949	ITGA1	integrin subunit alpha 1
ENSG00000213967	ZNF726	zinc finger protein 726
ENSG00000214013	GANC	glucosidase alpha, neutral C
ENSG00000214198	TTC41P	tetratricopeptide repeat domain 41, pseudogene
ENSG00000214425	LRRC37A4P	leucine rich repeat containing 37 member A4, pseudogene
ENSG00000215068		novel transcript, antisense to ANXA2R
ENSG00000215421	ZNF407	zinc finger protein 407
ENSG00000215483	LINC00598	long intergenic non-protein coding RNA 598
ENSG00000216937	CCDC7	coiled-coil domain containing 7
ENSG00000218175		ribosomal protein, large, P1 (RPLP1) pseudogene
ENSG00000219507	FTH1P8	ferritin heavy chain 1 pseudogene 8
ENSG00000220506		novel pseudogene
ENSG00000222114	RNU6-985P	RNA, U6 small nuclear 985, pseudogene
ENSG00000222378	RNA5SP44	RNA, 5S ribosomal pseudogene 44
ENSG00000222394		Y RNA [Source:RFAM;Acc:RF00019]
ENSG00000222465	RNU2-5P	RNA, U2 small nuclear 5, pseudogene
ENSG00000222610	RNU6-402P	RNA, U6 small nuclear 402, pseudogene
ENSG00000223062	RNU6-1245P	RNA, U6 small nuclear 1245, pseudogene
ENSG00000223126	RN7SKP263	RN7SK pseudogene 263
ENSG00000223392	CLDN10-AS1	CLDN10 antisense RNA 1
ENSG00000223813		novel transcript, antisense to CHN2
ENSG00000224078	SNHG14	small nucleolar RNA host gene 14
ENSG00000224510	POLR2KP2	RNA polymerase II subunit K pseudogene 2
ENSG00000224897	POT1-AS1	POT1 antisense RNA 1
ENSG00000225830	ERCC6	ERCC excision repair 6, chromatin remodeling factor
ENSG00000225914	TSBP1-AS1	TSBP1 and BTNL2 antisense RNA 1
ENSG00000226084		ribosomal protein L17 (RPL17) pseudogene
ENSG00000226439		ribosomal protein S12 (RPS12) pseudogene
ENSG00000226650	KIF4B	kinesin family member 4B
ENSG00000226777	FAM30A	family with sequence similarity 30 member A
ENSG00000226935	LINC00161	long intergenic non-protein coding RNA 161
ENSG00000227589	TP73-AS3	TP73 antisense RNA 3
ENSG00000227615		ribosomal protein S12 (RPS12) pseudogene
ENSG00000228567	VN1R4	vomeronasal 1 receptor 4
ENSG00000228668	TRGV5P	T cell receptor gamma variable 5P (pseudogene)
ENSG00000228775	WEE2-AS1	WEE2 antisense RNA 1
ENSG00000229308		novel transcript

ENSG00000229585	RPL21P44	ribosomal protein L21 pseudogene 44
ENSG00000229721		ribosomal protein S8 (RPS8) pseudogene
ENSG00000229894	GK3P	glycerol kinase 3 pseudogene
ENSG00000230124	ACBD6	acyl-CoA binding domain containing 6
ENSG00000230438	SERPINB9P1	serpin family B member 9 pseudogene 1
ENSG00000230593	PPIAP40	peptidylprolyl isomerase A pseudogene 40
ENSG00000231544	RSL24D1P11	ribosomal L24 domain containing 1 pseudogene 11
ENSG00000231607	DLEU2	deleted in lymphocytic leukemia 2
ENSG00000231691		novel transcript
ENSG00000231892		novel transcript
ENSG00000232830		family with sequence similarity 136, member A (FAM136A) pseudogene
ENSG00000233230		novel transcript
ENSG00000233382	NKAPP1	NFKB activating protein pseudogene 1
ENSG00000233757		novel C2H2 type zinc finger protein
ENSG00000234043	NUDT9P1	nudix hydrolase 9 pseudogene 1
ENSG00000234062		transmembrane 9 superfamily member 2 (TM9SF2) pseudogene
ENSG00000234093	RPS15AP11	ribosomal protein S15a pseudogene 11
ENSG00000234810		novel transcript
ENSG00000234817	ECI2-DT	ECI2 divergent transcript
ENSG00000235082	SUMO1P3	SUMO1 pseudogene 3
ENSG00000235376	RPEL1	ribulose-5-phosphate-3-epimerase like 1
ENSG00000235387	SPAAR	small regulatory polypeptide of amino acid response
ENSG00000235770	LINC00607	long intergenic non-protein coding RNA 607
ENSG00000235989	MORC2-AS1	MORC2 antisense RNA 1
ENSG00000236533	RPS12P4	ribosomal protein S12 pseudogene 4
ENSG00000236859	NIFK-AS1	NIFK antisense RNA 1
ENSG00000237440	ZNF737	zinc finger protein 737
ENSG00000237506	RPSAP15	ribosomal protein SA pseudogene 15
ENSG00000238113	LINC01410	long intergenic non-protein coding RNA 1410
ENSG00000238269	PAGE2B	PAGE family member 2B
ENSG00000238324	RN7SKP198	RN7SK pseudogene 198
ENSG00000238444	RNU6-893P	RNA, U6 small nuclear 893, pseudogene
ENSG00000238561	RNU6ATAC28P	RNA, U6atac small nuclear 28, pseudogene
ENSG00000238943		Y RNA [Source:RFAM;Acc:RF00019]
ENSG00000239142		U8 small nucleolar RNA [Source:RFAM;Acc:RF00096]
ENSG00000239246		ribosomal protein S10 (RPS10) pseudogene
ENSG00000239388	ASB14	ankyrin repeat and SOCS box containing 14
ENSG00000239468	RN7SL569P	RNA, 7SL, cytoplasmic 569, pseudogene
ENSG00000240038	AMY2B	amylase alpha 2B
ENSG00000240457	RN7SL472P	RNA, 7SL, cytoplasmic 472, pseudogene
ENSG00000240625	RN7SL403P	RNA, 7SL, cytoplasmic 403, pseudogene
ENSG00000241058	NSUN6	NOP2/Sun RNA methyltransferase 6

ENSG00000241128	OR14A2	olfactory receptor family 14 subfamily A member 2
ENSG00000241243	RN7SL629P	RNA, 7SL, cytoplasmic 629, pseudogene
ENSG00000241352		ribosomal protein S6 (RPS6) pseudogene
ENSG00000241549	GUSBP2	GUSB pseudogene 2
ENSG00000242221	PSG2	pregnancy specific beta-1-glycoprotein 2
ENSG00000242989	RN7SL332P	RNA, 7SL, cytoplasmic 332, pseudogene
ENSG00000242999	RN7SL239P	RNA, 7SL, cytoplasmic 239, pseudogene
ENSG00000243156	MICAL3	microtubule associated monooxygenase, calponin and LIM domain containing 3
ENSG00000243641	OR13C7	olfactory receptor family 13 subfamily C member 7 (gene/pseudogene)
ENSG00000243779		ribosomal protein L36a-like (RPL36AL) pseudogene
ENSG00000243991	RN7SL447P	RNA, 7SL, cytoplasmic 447, pseudogene
ENSG00000244222	OR7E121P	olfactory receptor family 7 subfamily E member 121 pseudogene
ENSG00000244405	ETV5	ETS variant transcription factor 5
ENSG00000244754	N4BP2L2	NEDD4 binding protein 2 like 2
ENSG00000248905	FMN1	formin 1
ENSG00000250312	ZNF718	zinc finger protein 718
ENSG00000250490	LINC02145	long intergenic non-protein coding RNA 2145
ENSG00000250878	METTL21EP	methyltransferase like 21E, pseudogene
ENSG00000251192	ZNF674	zinc finger protein 674
ENSG00000251391		novel transcript
ENSG00000251562	MALAT1	metastasis associated lung adenocarcinoma transcript 1
ENSG00000251819	RNU6-322P	RNA, U6 small nuclear 322, pseudogene
ENSG00000251982	RN7SKP43	RN7SK pseudogene 43
ENSG00000252013	RNU4ATAC14P	RNA, U4atac small nuclear 14, pseudogene
ENSG00000252070	RNA5SP341	RNA, 5S ribosomal pseudogene 341
ENSG00000252083		
ENSG00000252128		Small nucleolar RNA SNORD27 [Source:RFAM;Acc:RF00086]
ENSG00000252238		
ENSG00000252322	RN7SKP244	RN7SK pseudogene 244
ENSG00000252377	RNU6-504P	RNA, U6 small nuclear 504, pseudogene
ENSG00000252421	RNU6-1069P	RNA, U6 small nuclear 1069, pseudogene
ENSG00000252428	RNA5SP285	RNA, 5S ribosomal pseudogene 285
ENSG00000252555	RNU6-567P	RNA, U6 small nuclear 567, pseudogene
ENSG00000252607		Y RNA [Source:RFAM;Acc:RF00019]
ENSG00000252642	RNA5SP137	RNA, 5S ribosomal pseudogene 137
ENSG00000252659	RNU6-1088P	RNA, U6 small nuclear 1088, pseudogene
ENSG00000252696	RNA5SP34	RNA, 5S ribosomal pseudogene 34
ENSG00000252718	RNU6-612P	RNA, U6 small nuclear 612, pseudogene
ENSG00000252719		
ENSG00000252747	RNA5SP364	RNA, 5S ribosomal pseudogene 364
ENSG00000252937	RNU6-1270P	RNA, U6 small nuclear 1270, pseudogene
ENSG00000252995	RNU6-667P	RNA, U6 small nuclear 667, pseudogene

ENSG00000253032	RNU6-299P	RNA, U6 small nuclear 299, pseudogene
ENSG00000253038	RNU6-706P	RNA, U6 small nuclear 706, pseudogene
ENSG00000253352	TUG1	taurine up-regulated 1
ENSG00000253661	ZFHX4-AS1	ZFHX4 antisense RNA 1
ENSG00000253926		novel transcript
ENSG00000254469		XRCC1 N-terminal domain containing 1-like [Source:NCBI gene (formerly Entrezgene);Acc:100133315]
ENSG00000254838	GVINP1	GTPase, very large interferon inducible pseudogene 1
ENSG00000255302	EID1	EP300 interacting inhibitor of differentiation 1
ENSG00000255647		novel transcript
ENSG00000256040	PAPPA-AS1	PAPPA antisense RNA 1
ENSG00000256209		novel transcript
ENSG00000257103	LSM14A	LSM14A mRNA processing body assembly factor
ENSG00000257261		novel transcript
ENSG00000257267	ZNF271P	zinc finger protein 271, pseudogene
ENSG00000258101		novel transcript, antisense to TUBA1C
ENSG00000258334		novel transcript, antisense to PRPH
ENSG00000258729		novel transcript
ENSG00000258817	OR4C13	olfactory receptor family 4 subfamily C member 13
ENSG00000259429	UBE2Q2P2	ubiquitin conjugating enzyme E2 Q2 pseudogene 2
ENSG00000260549	MT1L	metallothionein 1L, pseudogene
ENSG00000260804	LINC01963	long intergenic non-protein coding RNA 1963
ENSG00000261423	TMEM202-AS1	TMEM202 antisense RNA 1
ENSG00000261609	GAN	gigaxonin
ENSG00000261845		novel transcript, antisense FOXK2
ENSG00000262587		leucine carboxyl methyltransferase 1 (LCMT1) pseudogene
ENSG00000263327	TAPT1-AS1	TAPT1 antisense RNA 1 (head to head)
ENSG00000263353	PPIAL4A	peptidylprolyl isomerase A like 4A
ENSG00000264006	AKR1C8P	aldo-keto reductase family 1 member C8, pseudogene
ENSG00000264278	ZNF236-DT	ZNF236 divergent transcript
ENSG00000264554	RN7SL793P	RNA, 7SL, cytoplasmic 793, pseudogene
ENSG00000265107	GJA5	gap junction protein alpha 5
ENSG00000267041	ZNF850	zinc finger protein 850
ENSG00000267132	HMGB3P27	high mobility group box 3 pseudogene 27
ENSG00000267517	LINC01855	long intergenic non-protein coding RNA 1855
ENSG00000268364	SMC5-AS1	SMC5 antisense RNA 1 (head to head)
ENSG00000269514		novel transcript, antisense to OR10AD1
ENSG00000269929	MIRLET7A1HG	miRlet-7a-1/let-7f-1/let-7d cluster host gene
ENSG00000270647	TAF15	TATA-box binding protein associated factor 15
ENSG00000272599		novel transcript to NUDT13
ENSG00000272906		novel transcript, antisense to TOR1AIP1
ENSG00000274386	TMEM269	transmembrane protein 269
ENSG00000274736	CCL23	C-C motif chemokine ligand 23

ENSG00000274750	H3C6	H3 clustered histone 6
ENSG00000275385	CCL18	C-C motif chemokine ligand 18
ENSG00000276075		novel transcript, antisense to FAM65A
ENSG00000277157	H4C4	H4 clustered histone 4
ENSG00000277506	RN7SL802P	RNA, 7SL, cytoplasmic 802, pseudogene
ENSG00000278196	IGLV2-8	immunoglobulin lambda variable 2-8
ENSG00000278374		U4 spliceosomal RNA [Source:RFAM;Acc:RF00015]
ENSG00000278525	RN7SL607P	RNA, 7SL, cytoplasmic 607, pseudogene
ENSG00000279301	OR2T11	olfactory receptor family 2 subfamily T member 11
ENSG00000279364		TEC
ENSG00000279608		TEC
ENSG00000279719		novel transcript
ENSG00000282885		novel transcript
ENSG00000283564		U6 spliceosomal RNA [Source:RFAM;Acc:RF00026]

Table S2. Enriched biological processes and pathways for up- and down-regulated genes after exposure to high doses of γ-rays, collected 1-2hrs post irradiation.

**Gene Set**	**Description**	**FDR**
Up-Regulated Genes↑		
GO:0022613	ribonucleoprotein complex biogenesis	1.75E-11
GO:0016072	rRNA metabolic process	3.01E-07
GO:0034470	ncRNA processing	1.88E-06
GO:0072331	signal transduction by p53 class mediator	1.28E-05
GO:0042770	signal transduction in response to DNA damage	2.08E-05
GO:0006403	RNA localization	7.38E-05
GO:0010608	posttranscriptional regulation of gene expression	4.60E-04
GO:0034248	regulation of cellular amide metabolic process	0.001327868
GO:2001233	regulation of apoptotic signaling pathway	0.001434038
GO:0045862	positive regulation of proteolysis	0.001564745
GO:1903829	positive regulation of cellular protein localization	0.001953242
GO:0006457	protein folding	0.002298225
GO:0097193	intrinsic apoptotic signaling pathway	0.002302183
GO:0070661	leukocyte proliferation	0.003108069
GO:1902532	negative regulation of intracellular signal transduction	0.003924678
GO:0009615	response to virus	0.004562085
GO:0071166	ribonucleoprotein complex localization	0.005150308
GO:0006413	translational initiation	0.005219382
GO:0071900	regulation of protein serine/threonine kinase activity	0.005804333
GO:0045930	negative regulation of mitotic cell cycle	0.00809352
GO:0051348	negative regulation of transferase activity	0.009045194
GO:0009314	response to radiation	0.009151245
GO:0015931	nucleobase-containing compound transport	0.009151245
GO:0006354	DNA-templated transcription, elongation	0.009933599
R-HSA-8953854	Metabolism of RNA	1.95E-10
WP4286	Genotoxicity pathway	9.49E-08
hsa05169	Epstein-Barr virus infection	6.05E-07
R-HSA-3700989	Transcriptional Regulation by TP53	1.20E-06
R-HSA-6791226	Major pathway of rRNA processing in the nucleolus and cytosol	1.20E-06
R-HSA-72312	rRNA processing	1.20E-06
R-HSA-8868773	rRNA processing in the nucleus and cytosol	1.20E-06
hsa04115	p53 signaling pathway	4.12E-06
hsa03008	Ribosome biogenesis in eukaryotes	6.27E-06
R-HSA-389960	Formation of tubulin folding intermediates by CCT/TriC	1.28E-05

R-HSA-392499	Metabolism of proteins	1.44E-05
R-HSA-389958	Cooperation of Prefoldin and TriC/CCT in actin and tubulin folding	1.73E-05
WP1530	miRNA Regulation of DNA Damage Response	3.74E-05
R-HSA-389957	Prefoldin mediated transfer of substrate to CCT/TriC	4.37E-05
R-HSA-74160	Gene expression (Transcription)	4.37E-05
R-HSA-73857	RNA Polymerase II Transcription	1.12E-04
R-HSA-1280215	Cytokine Signaling in Immune system	3.87E-04
R-HSA-6790901	rRNA modification in the nucleus and cytosol	4.73E-04
WP707	DNA Damage Response	4.73E-04
R-HSA-212436	Generic Transcription Pathway	6.88E-04
R-HSA-390450	Folding of actin by CCT/TriC	6.88E-04
hsa05168	Herpes simplex infection	7.40E-04
R-HSA-190236	Signaling by FGFR	8.02E-04
hsa05166	Human T-cell leukemia virus 1 infection	0.001199481
R-HSA-5668541	TNFR2 non-canonical NF-kB pathway	0.001199481
R-HSA-68882	Mitotic Anaphase	0.001199481
R-HSA-2555396	Mitotic Metaphase and Anaphase	0.001231855
WP3941	Oxidative Damage	0.001313853
R-HSA-6803529	FGFR2 alternative splicing	0.001327868
hsa05330	Allograft rejection	0.001496383
WP3617	Photodynamic therapy-induced NF-kB survival signaling	0.001496383
WP1742	TP53 Network	0.001564745
WP1984	Integrated Breast Cancer Pathway	0.001564745
WP254	Apoptosis	0.001564745
WP3982	miRNA regulation of p53 pathway in prostate cancer	0.001583679
R-HSA-5654738	Signaling by FGFR2	0.001630981
R-HSA-8852276	The role of GTSE1 in G2/M progression after G2 checkpoint	0.001630981
P00006	Apoptosis signaling pathway	0.001978219
R-HSA-381042	PERK regulates gene expression	0.001980577
R-HSA-2467813	Separation of Sister Chromatids	0.002209751
hsa04940	Type I diabetes mellitus	0.00261137
hsa05164	Influenza A	0.00279012
R-HSA-69620	Cell Cycle Checkpoints	0.002800275
hsa03013	RNA transport	0.002920388
WP3596	miR-517 relationship with ARCN1 and USP1	0.002920388
WP231	TNF alpha Signaling Pathway	0.003514333
R-HSA-72203	Processing of Capped Intron-Containing Pre-mRNA	0.003554963
hsa05310	Asthma	0.003638335
WP4754	IL-18 signaling pathway	0.004141241
R-HSA-5663205	Infectious disease	0.004247697
R-HSA-390466	Chaperonin-mediated protein folding	0.004562085
R-HSA-5357801	Programmed Cell Death	0.004562085
R-HSA-202424	Downstream TCR signaling	0.004973236
R-HSA-72163	mRNA Splicing - Major Pathway	0.004973236
hsa05321	Inflammatory bowel disease (IBD)	0.005425333
R-HSA-72766	Translation	0.005804333
R-HSA-391251	Protein folding	0.005893592
WP1772	Apoptosis Modulation and Signaling	0.005893592
hsa04612	Antigen processing and presentation	0.007296195
R-HSA-2262752	Cellular responses to stress	0.007296195
R-HSA-381119	Unfolded Protein Response (UPR)	0.007296195
R-HSA-5675221	Negative regulation of MAPK pathway	0.007296195
R-HSA-109581	Apoptosis	0.008192405
R-HSA-5683057	MAPK family signaling cascades	0.008192405
R-HSA-72172	mRNA Splicing	0.008192405
hsa05332	Graft-versus-host disease	0.008350239
R-HSA-168256	Immune System	0.008350239
R-HSA-449147	Signaling by Interleukins	0.008902687
hsa05416	Viral myocarditis	0.009034727
R-HSA-202403	TCR signaling	0.009045194

hsa04640	Hematopoietic cell lineage	0.009151245
R-HSA-109606	Intrinsic Pathway for Apoptosis	0.009151245
R-HSA-162906	HIV Infection	0.009151245
Down-Regulated Genes↓		
hsa04068	FoxO signaling pathway	5.80E-04
WP710	DNA Damage Response (only ATM dependent)	0.002169335
P00047	PDGF signaling pathway	0.002446475
hsa04140	Autophagy	0.004449471
P00018	EGF receptor signaling pathway	0.007375195
hsa04012	ErbB signaling pathway	0.009145883

Table S3. Enriched biological processes and pathways for up- and down-regulated genes after exposure to high doses of HZE, collected 6-24hrs post irradiation.

Gene Set	Description	FDR
Up-Regulated Genes↑		
GO:0002446	neutrophil mediated immunity	0.001203968
GO:0036230	granulocyte activation	0.001389131
hsa04142	Lysosome	1.70E-07
R-HSA-6798695	Neutrophil degranulation	3.99E-04
WP4286	Genotoxicity pathway	0.001389131
hsa00600	Sphingolipid metabolism	0.009444878
Down-Regulated Genes↓		
GO:0007059	chromosome segregation	1.32E-11
GO:0071103	DNA conformation change	1.69E-10
GO:0048285	organelle fission	4.22E-10
GO:0031023	microtubule organizing center organization	1.21E-09
GO:1901987	regulation of cell cycle phase transition	3.35E-08
GO:0044772	mitotic cell cycle phase transition	8.30E-08
GO:0044839	cell cycle G2/M phase transition	8.30E-08
GO:0033044	regulation of chromosome organization	1.35E-06
GO:0051052	regulation of DNA metabolic process	2.22E-06
GO:0006302	double-strand break repair	3.85E-06
GO:0006260	DNA replication	4.57E-06
GO:0044782	cilium organization	5.62E-06
GO:0006310	DNA recombination	7.58E-06
GO:0000075	cell cycle checkpoint	1.35E-05
GO:1902850	microtubule cytoskeleton organization involved in mitosis	2.52E-05
GO:0045930	negative regulation of mitotic cell cycle	7.70E-05
GO:0007051	spindle organization	9.23E-05
GO:0006338	chromatin remodeling	1.48E-04
GO:0007018	microtubule-based movement	1.89E-04
GO:0010948	negative regulation of cell cycle process	2.67E-04
GO:0051321	meiotic cell cycle	3.65E-04
GO:0000209	protein polyubiquitination	3.78E-04
GO:0022406	membrane docking	4.06E-04
GO:0071824	protein-DNA complex subunit organization	0.001080171
GO:2001020	regulation of response to DNA damage stimulus	0.001548817
GO:0030705	cytoskeleton-dependent intracellular transport	0.001573559
GO:0031122	cytoplasmic microtubule organization	0.001987014
GO:0045787	positive regulation of cell cycle	0.002571887
GO:0061641	CENP-A containing chromatin organization	0.002849705
GO:0070646	protein modification by small protein removal	0.0028695
GO:0006397	mRNA processing	0.003924006
GO:0032886	regulation of microtubule-based process	0.006855785
GO:0010498	proteasomal protein catabolic process	0.007504612
GO:0034502	protein localization to chromosome	0.008148733
GO:0008380	RNA splicing	0.009392214

R-HSA-1640170	Cell Cycle	0
R-HSA-68877	Mitotic Prometaphase	0
R-HSA-69278	Cell Cycle, Mitotic	5.78E-11
R-HSA-68886	M Phase	1.21E-09
R-HSA-69620	Cell Cycle Checkpoints	1.21E-09
R-HSA-141424	Amplification of signal from the kinetochores	2.35E-08
R-HSA-141444	Amplification of signal from unattached kinetochores via a MAD2 inhibitory signal	2.35E-08
R-HSA-2500257	Resolution of Sister Chromatid Cohesion	2.35E-08
R-HSA-69618	Mitotic Spindle Checkpoint	7.10E-08
R-HSA-5620912	Anchoring of the basal body to the plasma membrane	9.32E-07
R-HSA-5663220	RHO GTPases Activate Formins	1.69E-06
R-HSA-5617833	Cilium Assembly	3.71E-06
R-HSA-68882	Mitotic Anaphase	3.99E-06
R-HSA-2565942	Regulation of PLK1 Activity at G2/M Transition	4.01E-06
R-HSA-2555396	Mitotic Metaphase and Anaphase	4.32E-06
R-HSA-380270	Recruitment of mitotic centrosome proteins and complexes	9.78E-06
R-HSA-380287	Centrosome maturation	9.78E-06
R-HSA-2467813	Separation of Sister Chromatids	1.32E-05
R-HSA-73894	DNA Repair	1.40E-05
WP4536	Genes related to primary cilium development (based on CRISPR)	1.67E-05
R-HSA-8854518	AURKA Activation by TPX2	1.83E-05
R-HSA-380259	Loss of Nlp from mitotic centrosomes	3.31E-05
R-HSA-380284	Loss of proteins required for interphase microtubule organization from the centrosome	3.31E-05
R-HSA-69275	G2/M Transition	5.85E-05
R-HSA-70895	Branched-chain amino acid catabolism	5.85E-05
R-HSA-453274	Mitotic G2-G2/M phases	7.64E-05
R-HSA-380320	Recruitment of NuMA to mitotic centrosomes	7.70E-05
R-HSA-5693579	Homologous DNA Pairing and Strand Exchange	1.06E-04
WP4016	DNA IR-damage and cellular response via ATR	1.11E-04
R-HSA-5693554	Resolution of D-loop Structures through Synthesis-Dependent Strand Annealing (SDSA)	1.57E-04
R-HSA-74160	Gene expression (Transcription)	1.69E-04
R-HSA-5693616	Presynaptic phase of homologous DNA pairing and strand exchange	1.89E-04
R-HSA-1852241	Organelle biogenesis and maintenance	2.36E-04
R-HSA-5693537	Resolution of D-Loop Structures	3.10E-04
R-HSA-5693568	Resolution of D-loop Structures through Holliday Junction Intermediates	3.10E-04
hsa04120	Ubiquitin mediated proteolysis	4.52E-04
WP2446	Retinoblastoma Gene in Cancer	6.15E-04
hsa03440	Homologous recombination	9.46E-04
R-HSA-5693607	Processing of DNA double-strand break ends	0.001067765
R-HSA-5693538	Homology Directed Repair	0.001548817
R-HSA-5693567	HDR through Homologous Recombination (HRR) or Single Strand Annealing (SSA)	0.001665003
R-HSA-5685942	HDR through Homologous Recombination (HRR)	0.001722405
R-HSA-73857	RNA Polymerase II Transcription	0.00188002
WP179	Cell Cycle	0.002252422
R-HSA-983168	Antigen processing: Ubiquitination & Proteasome degradation	0.003072282
hsa04110	Cell cycle	0.003268196
R-HSA-69473	G2/M DNA damage checkpoint	0.004395125
WP1971	Integrated Cancer Pathway	0.004835263
R-HSA-5693532	DNA Double-Strand Break Repair	0.005140536
R-HSA-212436	Generic Transcription Pathway	0.006281083
R-HSA-2514853	Condensation of Prometaphase Chromosomes	0.007521245

Table S4. Enriched biological processes and pathways for up-regulated genes after exposure to high doses of X-rays, collected 8-24hrs post irradiation.

**Gene Set**	**Description**	**FDR**
Up-Regulated Genes↑		
GO:0072331	signal transduction by p53 class mediator	2.45E-10
GO:0042770	signal transduction in response to DNA damage	2.07E-06
GO:0104004	cellular response to environmental stimulus	1.23E-04
GO:0000075	cell cycle checkpoint	5.09E-04
GO:0009314	response to radiation	6.23E-04
GO:0045787	positive regulation of cell cycle	8.89E-04
GO:0045862	positive regulation of proteolysis	0.003880987
GO:0007050	cell cycle arrest	0.00594055
GO:0097193	intrinsic apoptotic signaling pathway	0.006625582
GO:0045930	negative regulation of mitotic cell cycle	0.008514906
WP4286	Genotoxicity pathway	0
R-HSA-212436	Generic Transcription Pathway	1.98E-11
R-HSA-73857	RNA Polymerase II Transcription	4.55E-11
R-HSA-3700989	Transcriptional Regulation by TP53	1.24E-10
hsa04115	p53 signaling pathway	5.73E-10
R-HSA-74160	Gene expression (Transcription)	1.41E-09
WP1530	miRNA Regulation of DNA Damage Response	2.07E-06
P00059	p53 pathway	3.43E-06
WP707	DNA Damage Response	1.09E-05
WP3982	miRNA regulation of p53 pathway in prostate cancer	2.95E-05
R-HSA-6791312	TP53 Regulates Transcription of Cell Cycle Genes	1.37E-04
R-HSA-5633007	Regulation of TP53 Activity	6.23E-04
R-HSA-5633008	TP53 Regulates Transcription of Cell Death Genes	6.91E-04
R-HSA-6804756	Regulation of TP53 Activity through Phosphorylation	0.001237722
WP1742	TP53 Network	0.001237722
hsa04068	FoxO signaling pathway	0.002554446
hsa01524	Platinum drug resistance	0.006339389
